# Supplementary material for: Substrate multiplexed protein engineering facilitates promiscuous biocatalytic synthesis
Source: Nat Commun. 2022 Sep 6;13:5242. doi: 10.1038/s41467-022-32789-w (PMC9448781; doi:10.1038/s41467-022-32789-w)
Supplement: Supplementary file 1 — Supplementary Information [file 41467_2022_32789_MOESM1_ESM.pdf]

Supplementary Information

Substrate multiplexed protein engineering facilitates promiscuous biocatalytic synthesis

Allwin D. McDonald[1,2], Peyton M. Higgins[1,2], Andrew R. Buller[1,]*

[1]*Department of Chemistry, University of Wisconsin−Madison, 1101 University Avenue, Madison, Wisconsin 53706, United States*

[2]*These authors contributed equally to this work*

* To whom correspondence should be addressed: arbuller@wisc.edu

Table of Contents

Supplementary Figures
Supplementary Tables
Supplementary Discussion
- **Introduction to substrate competition kinetics behind substrate multiplexed screening (SUMS)**
- **Guide to optimization of SUMS method**

Supplementary Methods
- **General experimental methods**
- **Cloning and expression of *Rgn*TDC site-saturation libraries**
- **Screening of *Rgn*TDC site-saturation libraries**
- **Cloning of *Pf*TrpB globally random mutagenesis libraries**
- **Cloning of *Pf*TrpB H275X site-saturation library**
- **Screening of *Pf*TrpB libraries**
- **Expression of *Rgn*TDC and *Pf*TrpB variants**
- **Turnover analysis of *Rgn*TDC variants**
- **[PLP] vs. activity for *Rgn*TDC variants**
- ***Rgn*TDC substrate multiplexed timecourse reaction**
- **Michaelis-Menten analysis of *Rgn*TDC variants**
- **Single substrate activity analysis of *Pf*TrpB variants**
- **Michaelis-Menten analysis of *Pf*TrpB variants**
- **UV-Vis spectroscopy of *Pf*TrpB variants**
- **Protein crystallography of *Pf*TrpB-H275E**
- **Tryptophan analog biosynthesis and purification**
- **Cascade synthesis and isolation of tryptamines**
- **NMR spectra**

Supplementary References

Supplementary Figures

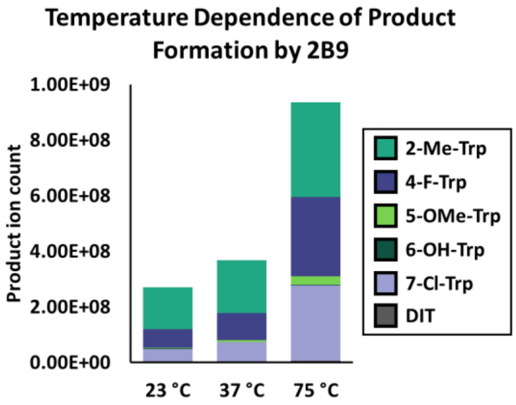

Supplementary Figure 1. The activity of *Pf*2B9 is temperature dependent. Reactions were run with a multiplexed substrate panel, 5 mM Ser, and 6.8 µM 2B9 in 100 mM KP_i buffer (pH = 8.0) and quenched after 10 minutes. Substrate panel: 5 mM each of 2-Me-indole, 5-OMe-indole, 6-OH-indole, and indoline and 2.5 mM each of 4-F-indole and 7-Cl-indole.

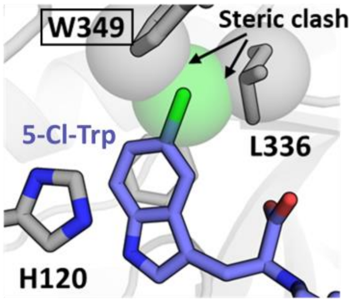

Supplementary Figure 2. *Rgn*TDC active-site model of 5-chlorotryptophan, modelled from PDB ID: 4OBV.[1] The potential steric interaction of the 5-chloro-substituent with the W349 and L336 residues is highlighted. Figure adapted from McDonald et al. 2019, with permission.[2]

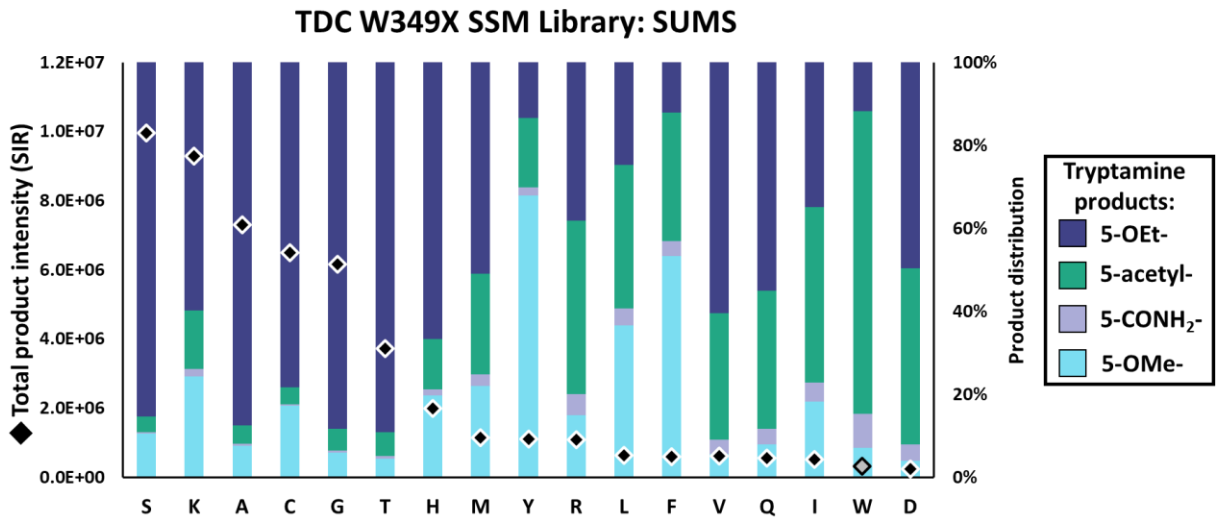

Supplementary Figure 3. Results from substrate multiplexed screening (SUMS) of the *Rgn*TDC W349 site-saturation mutagenesis (SSM) library with various 5-substituted tryptophan analogs. Colored bars represent relative amounts of each product formed, and black diamonds indicate total intensity of single ion retention (SIR) from each product's unique m/z. The wild-type sequence is denoted by a grey diamond. Relative product amounts and SIR intensity were averaged from all wells with the given sequence. 2-methyl-5-methoxytryptophan was also included as a potential substrate, but no product was observed. The sampling rate of each mutation is detailed in Supplementary Table 4.

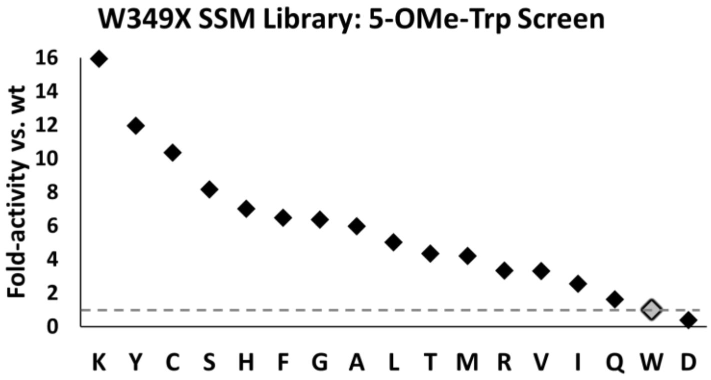

Supplementary Figure 4. Single-substrate screen of the *Rgn*TDC W349 site-saturation mutagenesis library with 5-methoxytryptophan. Fold-activities were determined relative to wild-type (wt) *Rgn*TDC wells from absorbance at 280 nm. Depicted fold-activities represent averaged fold-activities relative to wt activity for all wells with the above given sequence.

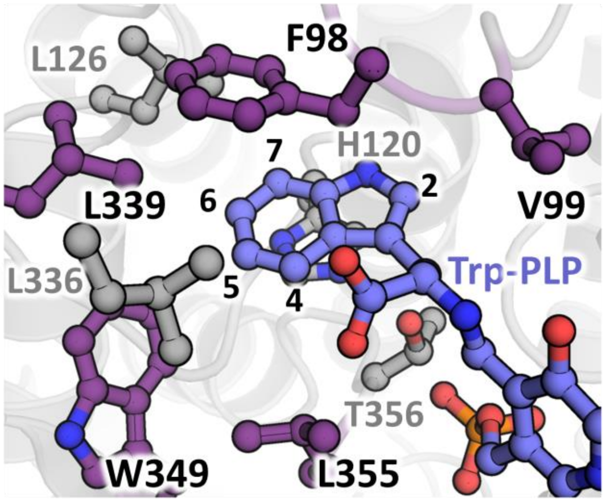

Supplementary Figure 5. Active-site model of *Rgn*TDC (built from PDB ID: 4OBV).[1] Residues highlighted in purple are sites at which mutations were found that significantly altered promiscuity or improved activity. Mutations at residues highlighted in gray were found to not significantly alter promiscuity (H120, L126) or resulted in catalytically feeble enzymes (L336, T356).

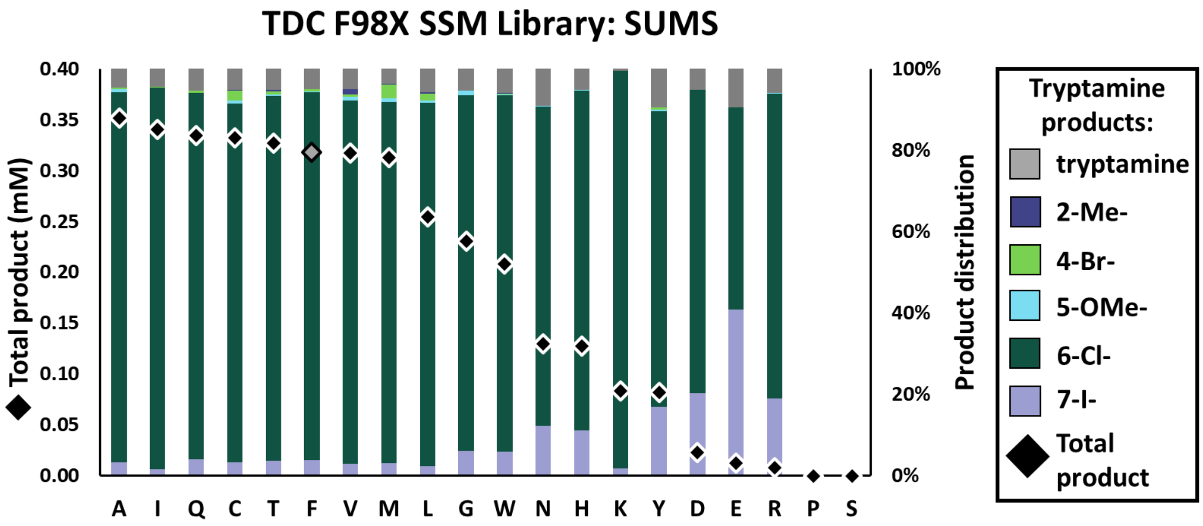

Supplementary Figure 6. Retention of function (ROF) curve from SUMS of the *Rgn*TDC F98 site-saturation mutagenesis library. Colored bars represent relative amounts of each product formed, and diamonds represent mM total product produced, as determined by single-ion retention standard curves. The wild-type sequence is denoted by a grey diamond. Relative product amounts and mM total product were averaged from all wells with the given sequence. (No proline or serine mutations were sequenced from this library). The sampling rate of each mutation is detailed in Supplementary Table 4.

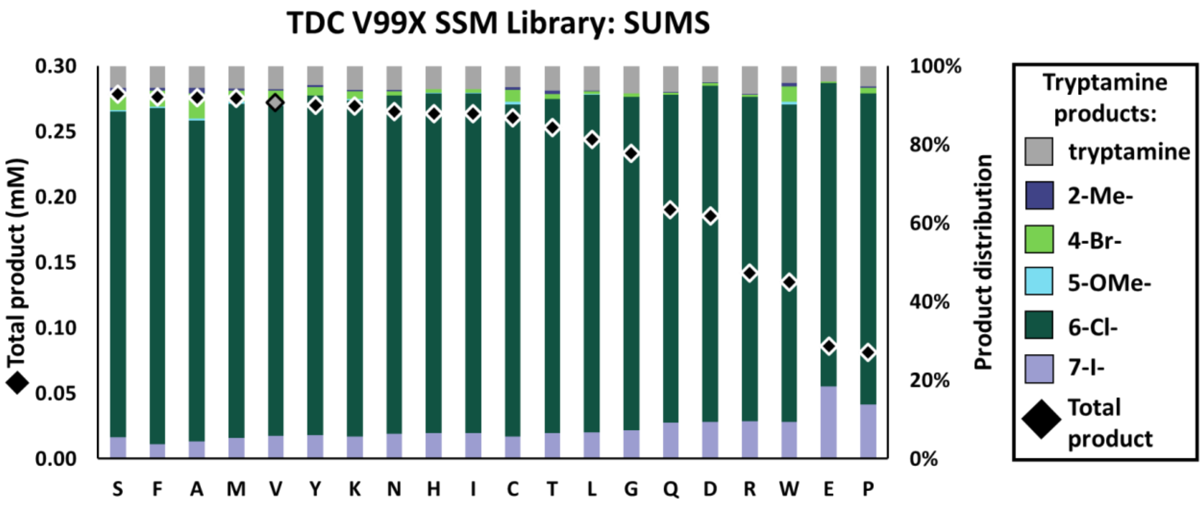

Supplementary Figure 7. Retention of function (ROF) curve from SUMS of the *Rgn*TDC V99 site-saturation mutagenesis library. Colored bars represent relative amounts of each product formed, and diamonds represent mM total product produced, as determined by single-ion retention standard curves. The wild-type sequence is denoted by a grey diamond. Relative product amounts and mM total product were averaged from all wells with the given sequence. The sampling rate of each mutation is detailed in Supplementary Table 4.

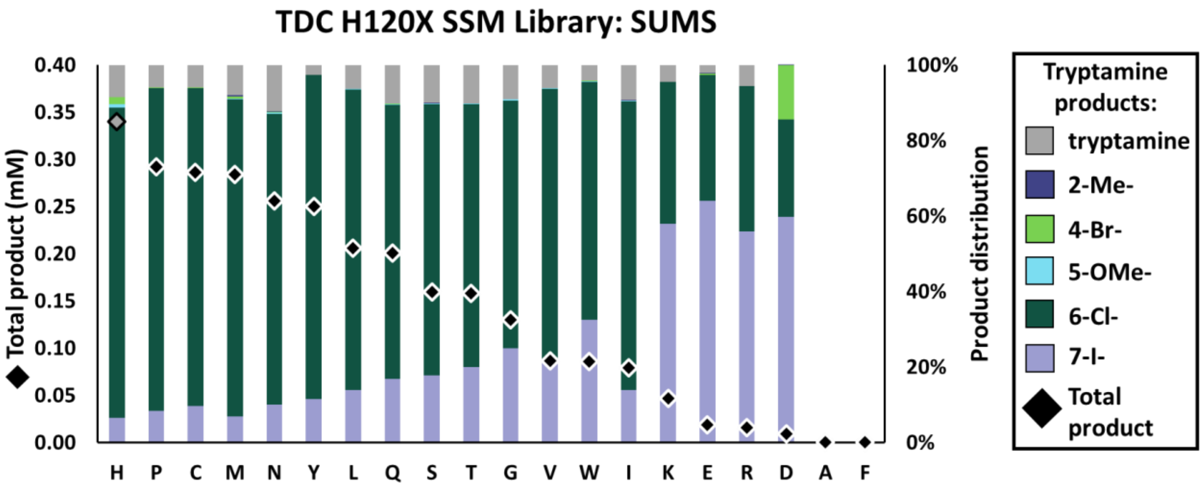

Supplementary Figure 8. Retention of function (ROF) curve from SUMS of the *Rgn*TDC H120 site-saturation mutagenesis library. Colored bars represent relative amounts of each product formed, and diamonds represent mM total product produced, as determined by single-ion retention standard curves. The wild-type sequence is denoted by a grey diamond. Relative product amounts and mM total product were averaged from all wells with the given sequence. (No alanine or phenylalanine mutations were sequenced from the library). The sampling rate of each mutation is detailed in Supplementary Table 4.

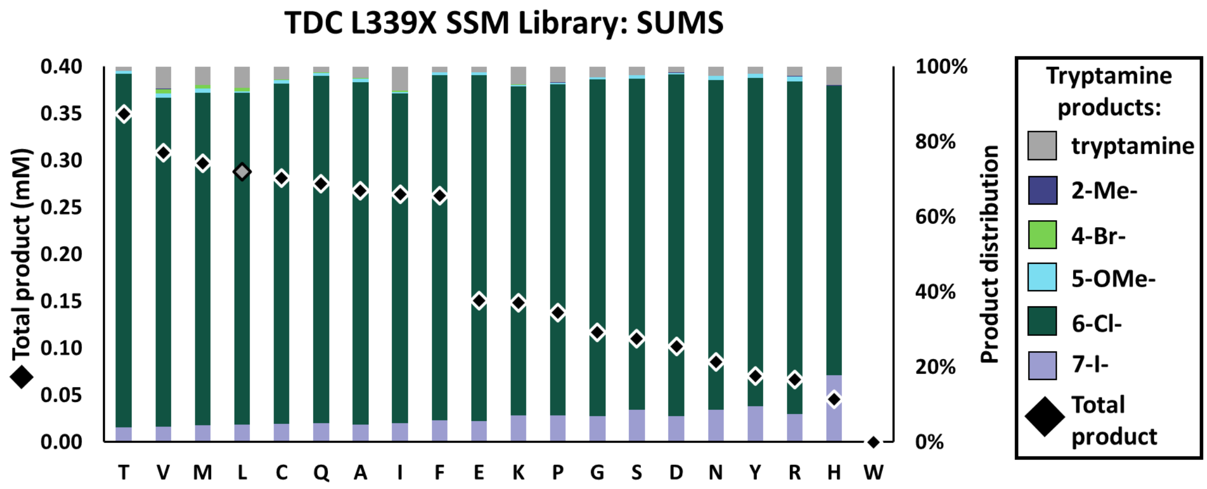

Supplementary Figure 9. Retention of function (ROF) curve from SUMS of the *Rgn*TDC L339 site-saturation mutagenesis library. Colored bars represent relative amounts of each product formed, and diamonds represent mM total product produced, as determined by single-ion retention standard curves. The wild-type sequence is denoted by a grey diamond. Relative product amounts and mM total product were averaged from all wells with the given sequence. (No tryptophan mutations were sequenced from the library). The sampling rate of each mutation is detailed in Supplementary Table 4.

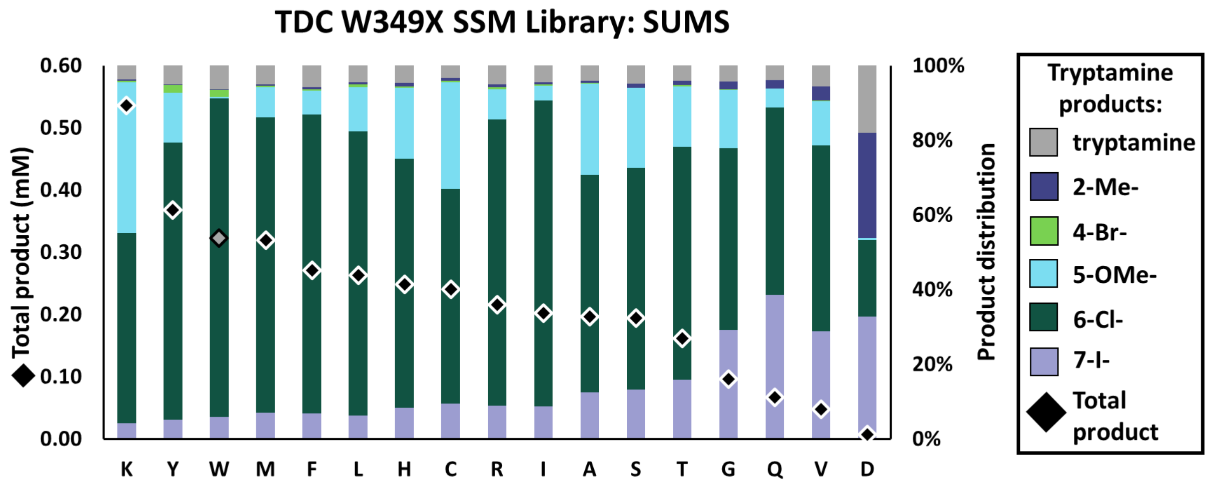

Supplementary Figure 10. Retention of function (ROF) curve from SUMS of the *Rgn*TDC W349 site-saturation mutagenesis library. Colored bars represent relative amounts of each product formed, and diamonds represent mM total product produced, as determined by single-ion retention standard curves. The wild-type sequence is denoted by a grey diamond. Relative product amounts and mM total product were averaged from all wells with the given sequence. The sampling rate of each mutation is detailed in Supplementary Table 4.

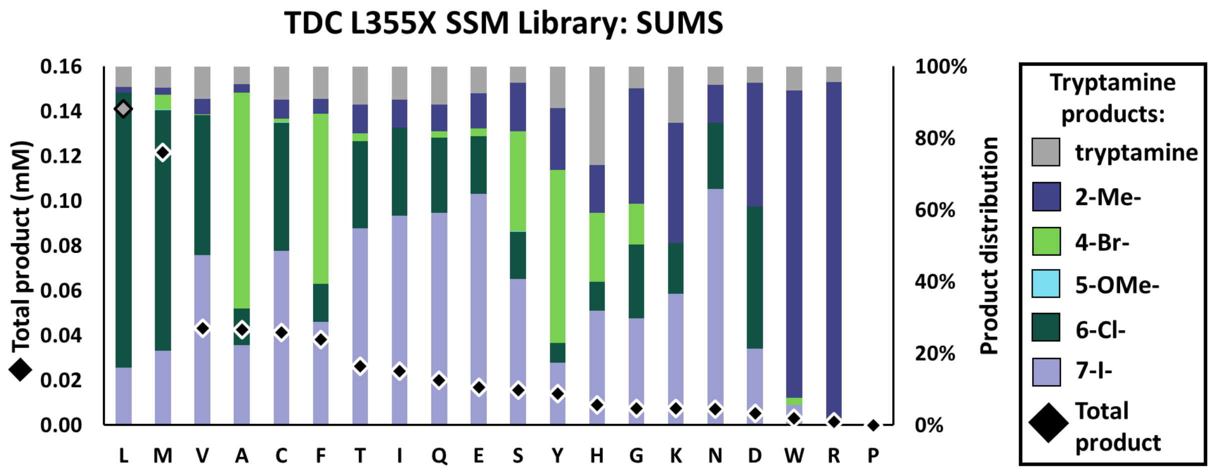

Supplementary Figure 11. Retention of function (ROF) curve from SUMS of the *Rgn*TDC L355 site-saturation mutagenesis library. Colored bars represent relative amounts of each product formed, and diamonds represent mM total product produced, as determined by single-ion retention standard curves. The wild-type sequence is denoted by a grey diamond. Relative product amounts and mM total product were averaged from all wells with the given sequence. (No proline mutations were sequenced from the library). The sampling rate of each mutation is detailed in Supplementary Table 4.

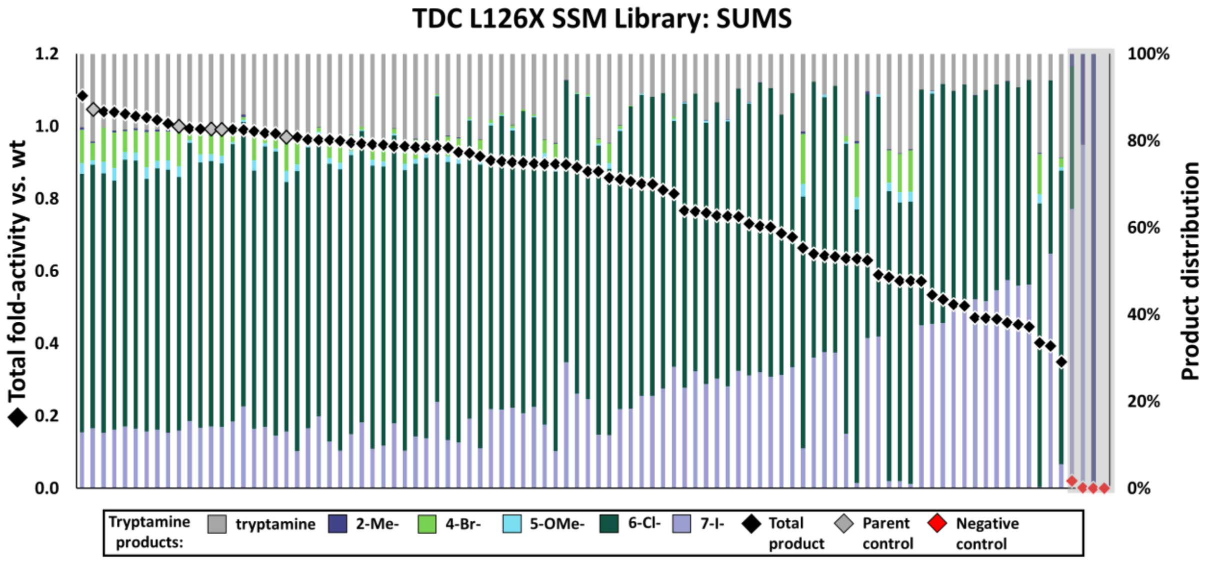

Supplementary Figure 12. Retention of function (ROF) curve from SUMS of the *Rgn*TDC L126 site-saturation mutagenesis library. Colored bars represent relative amounts of each product formed, and diamonds represent mM total product produced, as determined by single-ion retention standard curves. Wild-type sequences are denoted by grey diamonds, while negative controls are shown as red diamonds. Greyed-out section indicates wells where the quality of signal-to-noise was too low and the relative product distributions are dominated by noise and therefore no longer indicative of enzyme promiscuity.

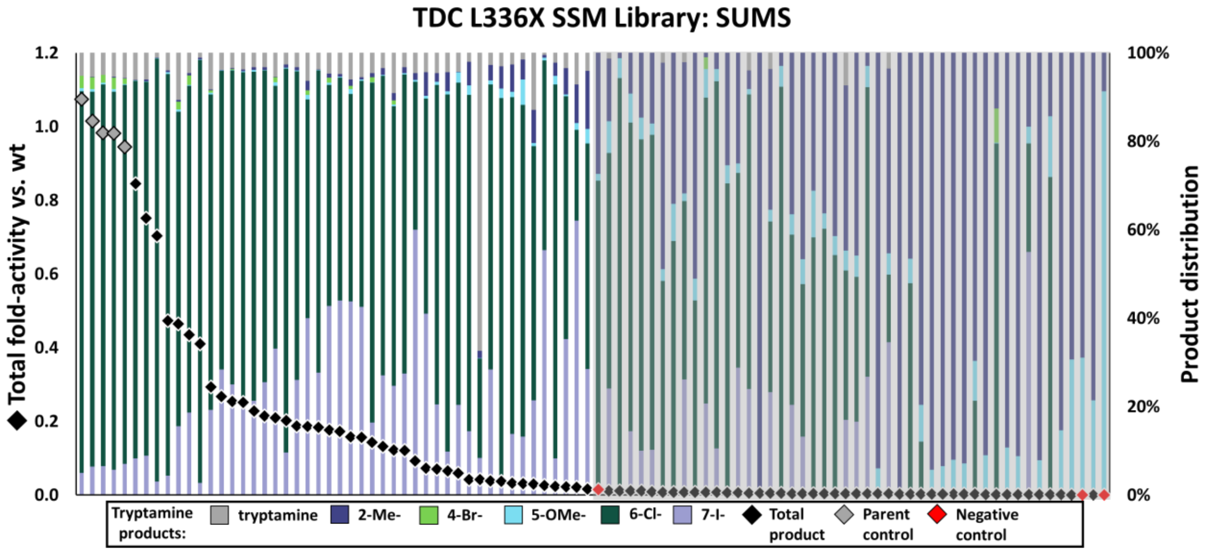

Supplementary Figure 13. Retention of function (ROF) curve from SUMS of the *Rgn*TDC L336 site-saturation mutagenesis library. Colored bars represent relative amounts of each product formed, and diamonds represent mM total product produced, as determined by single-ion retention standard curves. Wild-type sequences are denoted by grey diamonds, while negative controls are shown as red diamonds. Greyed-out section indicates wells where the quality of signal-to-noise was too low and the relative product distributions are dominated by noise and therefore no longer indicative of enzyme promiscuity.

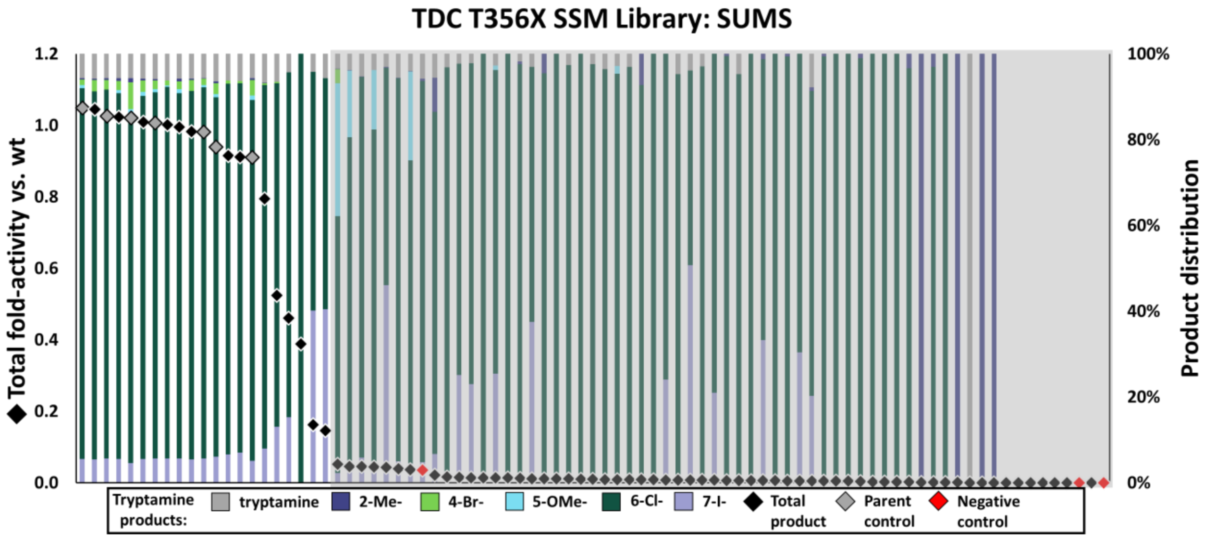

Supplementary Figure 14. Retention of function (ROF) curve from SUMS of the *Rgn*TDC T356 site-saturation mutagenesis library. Colored bars represent relative amounts of each product formed, and diamonds represent mM total product produced, as determined by single-ion retention standard curves. Wild-type sequences are denoted by grey diamonds, while negative controls are shown as red diamonds. Greyed-out section indicates wells where the quality of signal-to-noise was too low and the relative product distributions are dominated by noise and therefore no longer indicative of enzyme promiscuity.

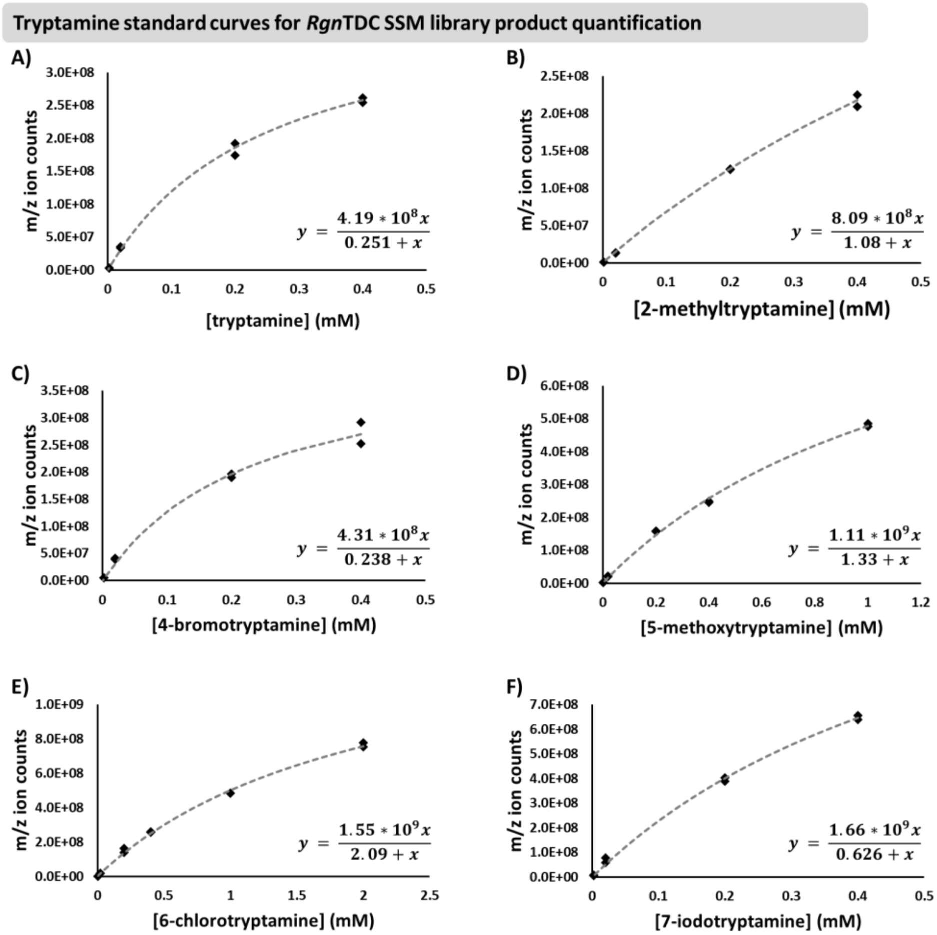

Supplementary Figure 15. Tryptamine standard curves for analysis of product abundance in *Rgn*TDC SSM libraries. A) Standard curve for tryptamine. **B)** Standard curve for 2-methyltryptamine. **C)** Standard curve for 4-bromotryptamine. **D)** Standard curve for 5-methoxytryptamine. **E)** Standard curve for 6-chlorotryptamine. **F)** Standard curve for 7-iodotryptamine. *Data were fit to hyperbolic equations.

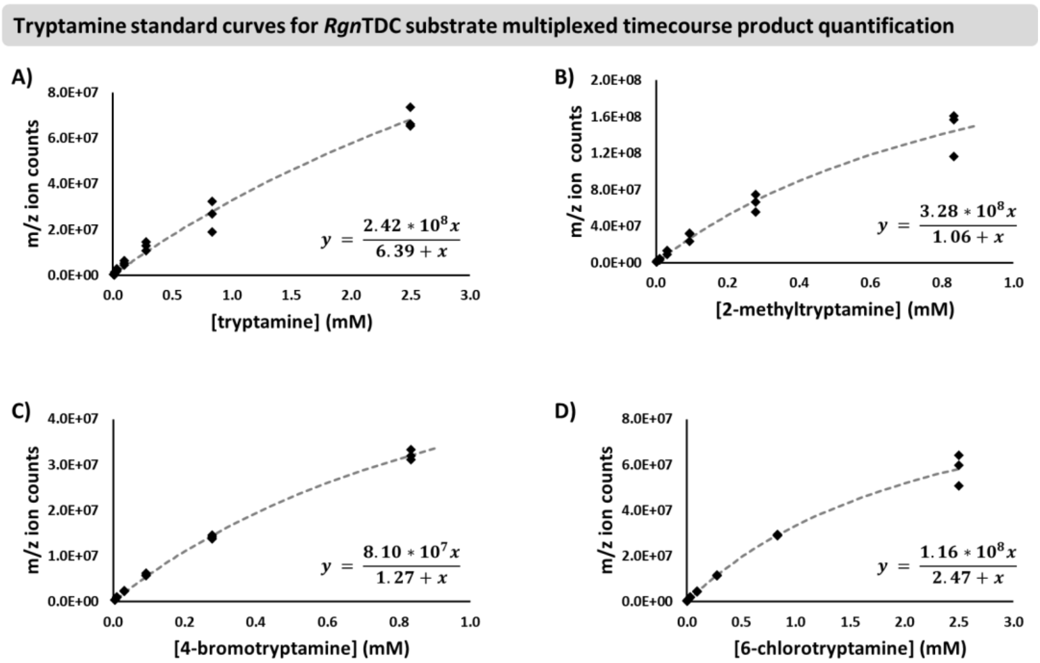

Supplementary Figure 16. Tryptamine standard curves for analysis of product abundance in the *Rgn*TDC substrate multiplexed timecourse reactions. A) Standard curve for tryptamine. **B)** Standard curve for 2-methyltryptamine. **C)** Standard curve for 4-bromotryptamine. **D)** Standard curve for 6-chlorotryptamine. *Data were fit to hyperbolic equations.

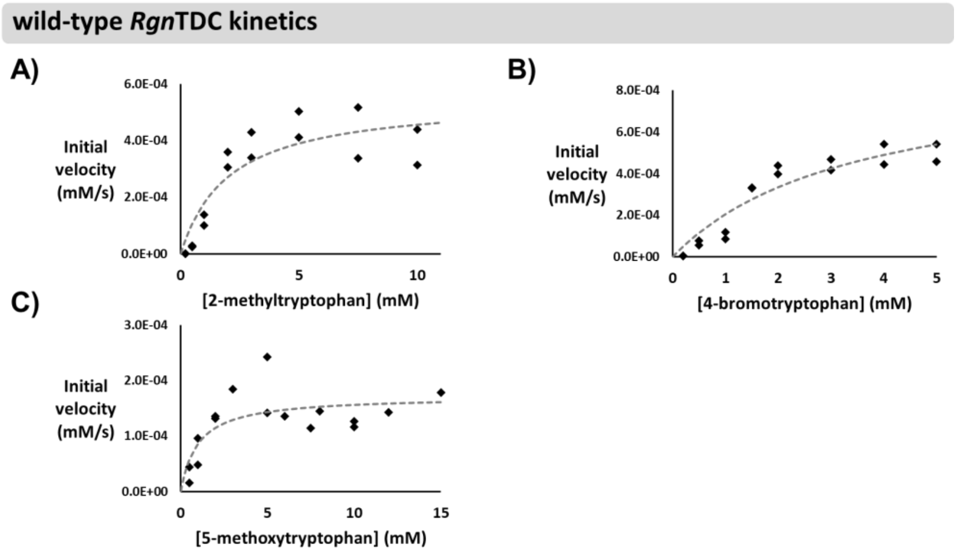

Supplementary Figure 17. Michaelis-Menten curves for wild-type *Rgn*TDC with 2-methyltryptophan, 4-bromotryptophan, and 5-methoxytryptophan. Relative enzyme concentration used are as follows: **A)** 10 µM *Rgn*TDC, **B)** 2.5 µM *Rgn*TDC, **C)** 10 µM *Rgn*TDC.

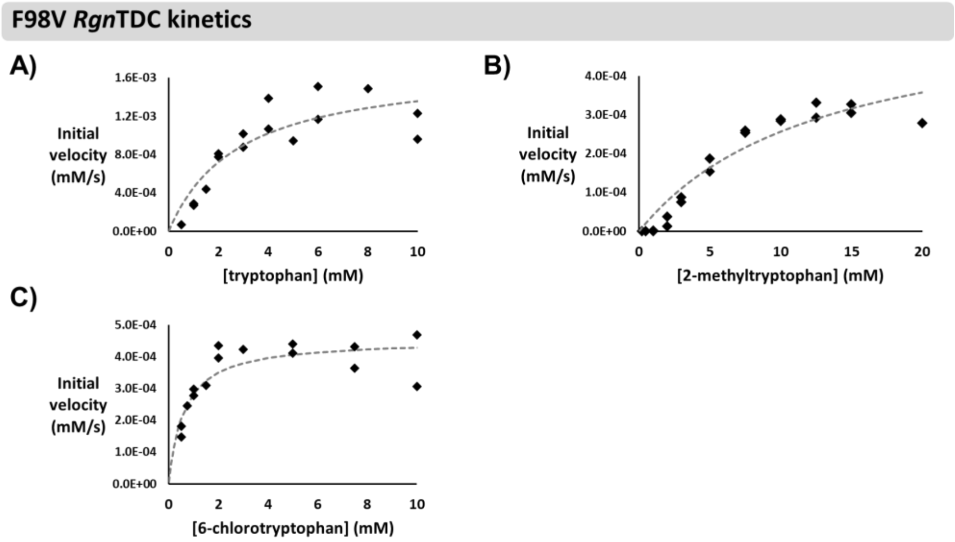

Supplementary Figure 18. Michaelis-Menten curves for F98V *Rgn*TDC with tryptophan, 2-methyltryptophan, and 6-chlorotryptophan. Relative enzyme concentration used are as follows: **A)** 0.2 μM *Rgn*TDC, **B)** 14 μM *Rgn*TDC, **C)** 0.5 μM *Rgn*TDC.

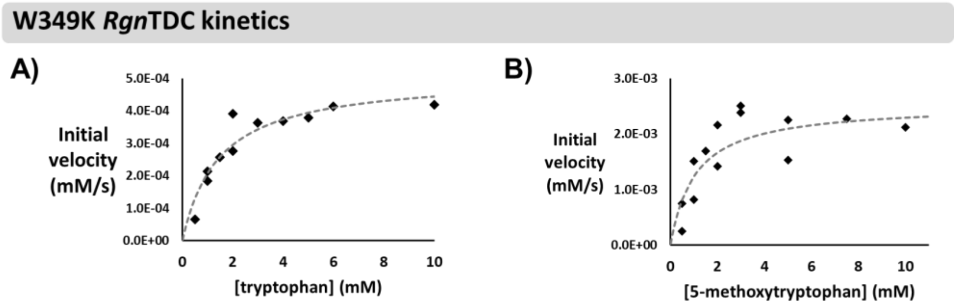

Supplementary Figure 19. Michaelis-Menten curves for W349K *Rgn*TDC with tryptophan and 5-methoxytryptophan. Relative enzyme concentration used are as follows: **A)** 0.5 μM *Rgn*TDC, **B)** 10 μM *Rgn*TDC.

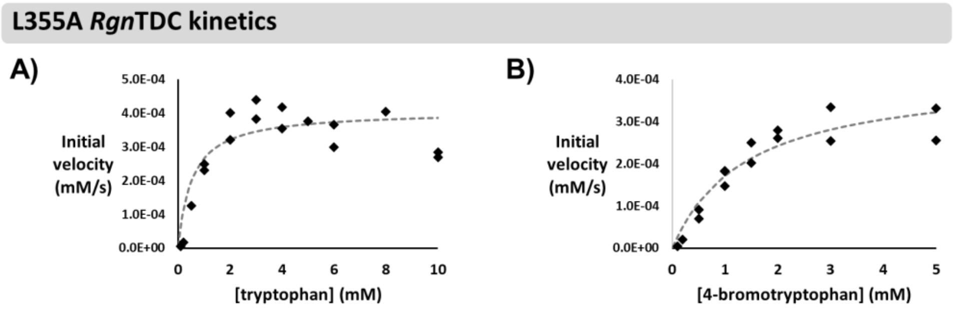

Supplementary Figure 20. Michaelis-Menten curves for L355A *Rgn*TDC with tryptophan and 4-bromotryptophan. Relative enzyme concentration used are as follows: **A)** 3 μM *Rgn*TDC, **B)** 0.5 μM *Rgn*TDC**.**

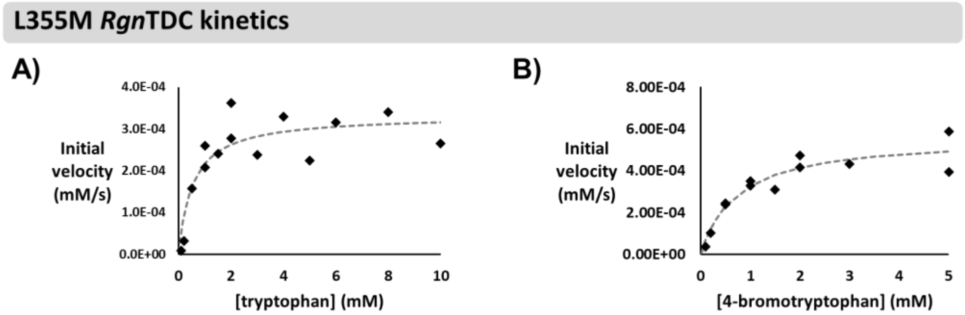

Supplementary Figure 21. Michaelis-Menten curves for L355M *Rgn*TDC with tryptophan and 4-bromotryptophan. Relative enzyme concentration used are as follows: **A)** 0.1 μM *Rgn*TDC, **B)** 0.5 μM *Rgn*TDC.

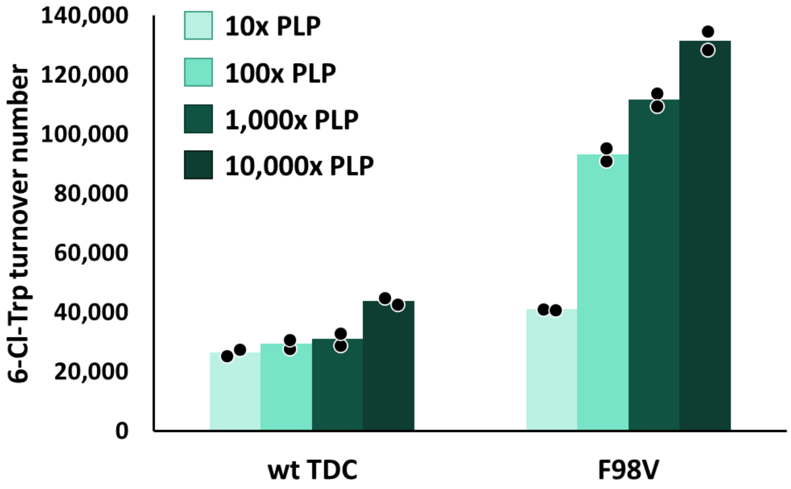

Supplementary Figure 22. Comparison of effect of additional pyridoxal phosphate (PLP) on conversion of 6-chlorotryptophan (6-Cl-Trp) to 6-chlorotryptamine. Amount of PLP shown is relative to the *Rgn*TDC concentration of 50 nM. Reactions were conducted in duplicate. F98V *Rgn*TDC showed a higher dependence on PLP than wild-type (wt) *Rgn*TDC, potentially indicating weaker cofactor binding or cofactor destruction.

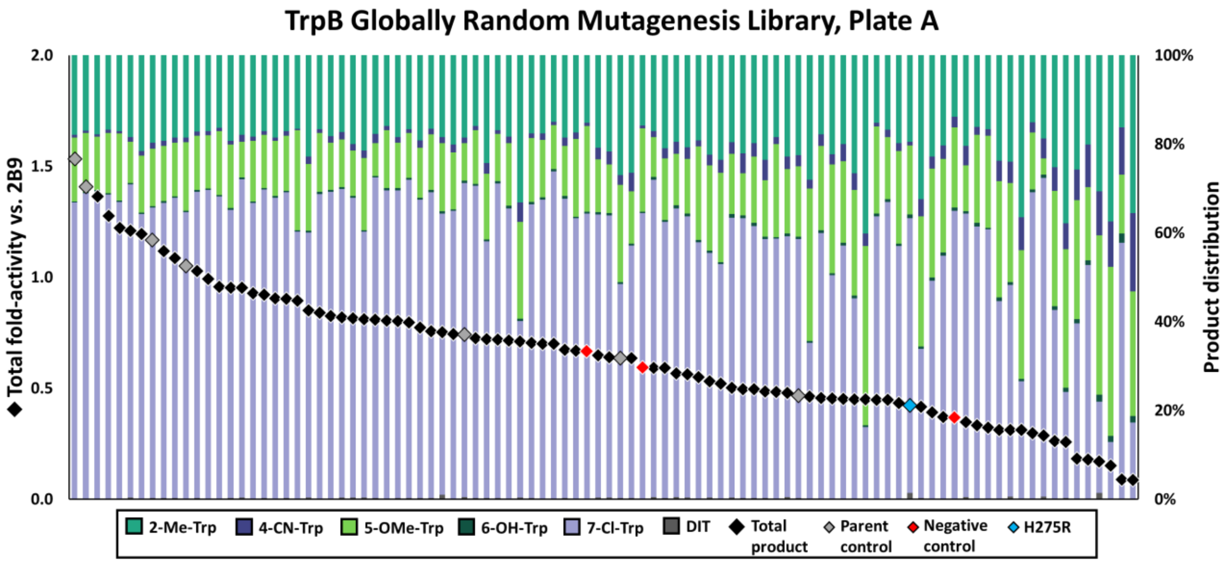

Supplementary Figure 23. SUMS results for plate A of the *Pf*TrpB globally random mutagenesis library, with 2B9 as the parent enzyme. Colored bars represent relative amounts of each product formed, and diamonds represent total product formed, based on ion count integrations of single-ion retention (SIR) channels. Parent (2B9) controls are denoted by grey diamonds, while negative controls are shown as red diamonds. Substrate panel: 5 mM each of 2-Me-indole, 4-CN-indole, 5-OMe-indole, 6-OH-indole, and indoline; and 2.5 mM 7-Cl-indole. The signal-to noise ratio throughout plate A was generally poor, but variant H275R was identified in this screen and subsequently validated in both substrate-multiplexed and single-substrate reactions.

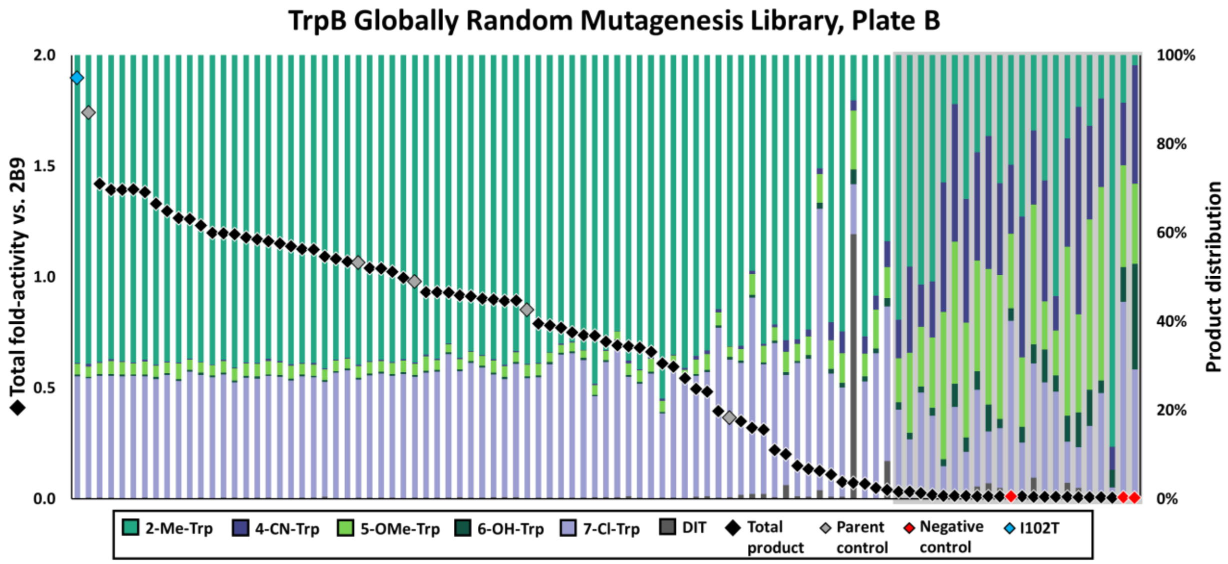

Supplementary Figure 24. SUMS results for plate B of the *Pf*TrpB globally random mutagenesis library, with 2B9 as the parent enzyme. Colored bars represent relative amounts of each product formed, and diamonds represent total product formed, based on ion count integrations of single-ion retention (SIR) channels. Parent (2B9) controls are denoted by grey diamonds, while negative controls are shown as red diamonds. Substrate panel: 5 mM each of 2-Me-indole, 4-CN-indole, 5-OMe-indole, 6-OH-indole, and indoline; and 2.5 mM 7-Cl-indole. Greyed-out section indicates wells where the quality of signal-to-noise was too low and the relative product distributions are dominated by noise and therefore no longer indicative of enzyme promiscuity.

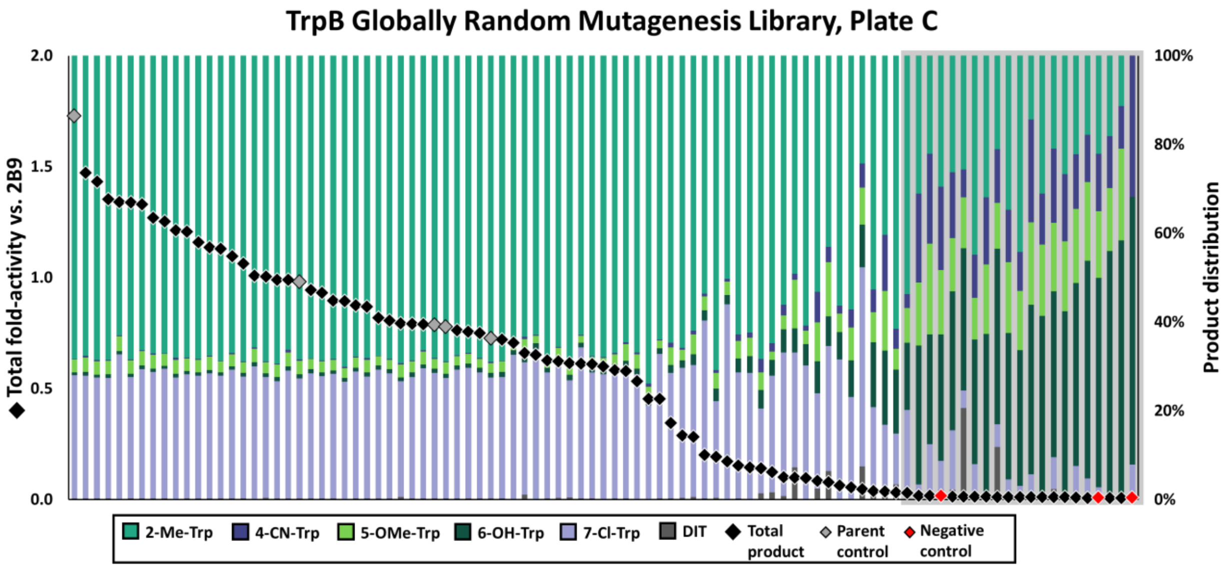

Supplementary Figure 25. SUMS results for plate C of the *Pf*TrpB globally random mutagenesis library, with 2B9 as the parent enzyme. Colored bars represent relative amounts of each product formed, and diamonds represent total product formed, based on ion count integrations of single-ion retention (SIR) channels. Parent (2B9) controls are denoted by grey diamonds, while negative controls are shown as red diamonds. Substrate panel: 5 mM each of 2-Me-indole, 4-CN-indole, 5-OMe-indole, 6-OH-indole, and indoline; and 2.5 mM 7-Cl-indole. Greyed-out section indicates wells where the quality of signal-to-noise was too low and the relative product distributions are dominated by noise and therefore no longer indicative of enzyme promiscuity.

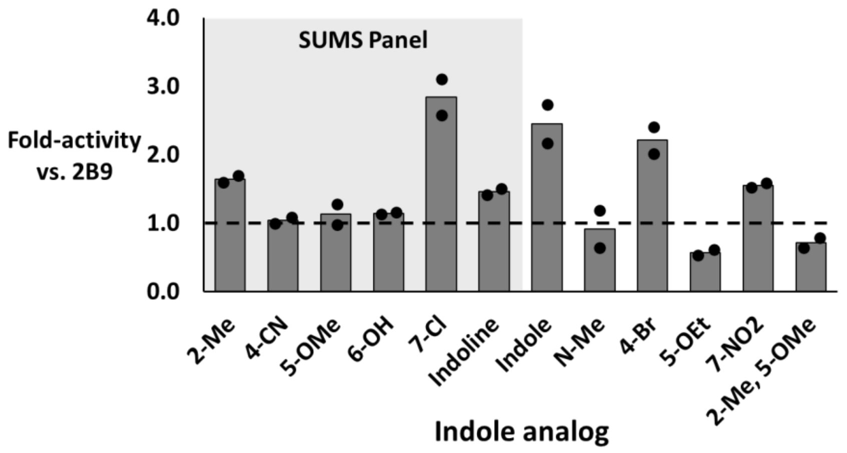

Supplementary Figure 26. *Pf*TrpB I102T single substrate activity. Activity of I102T in single substrate reactions is shown relative to 2B9 (black dashed line). Reactions consisted of 10 mM indole substrate, 30 mM Ser, and 2 µM *Pf*TrpB in 100 mM potassium phosphate buffer (pH = 8.0) and 10% DMSO. Reactions were run in duplicate at 37 °C.

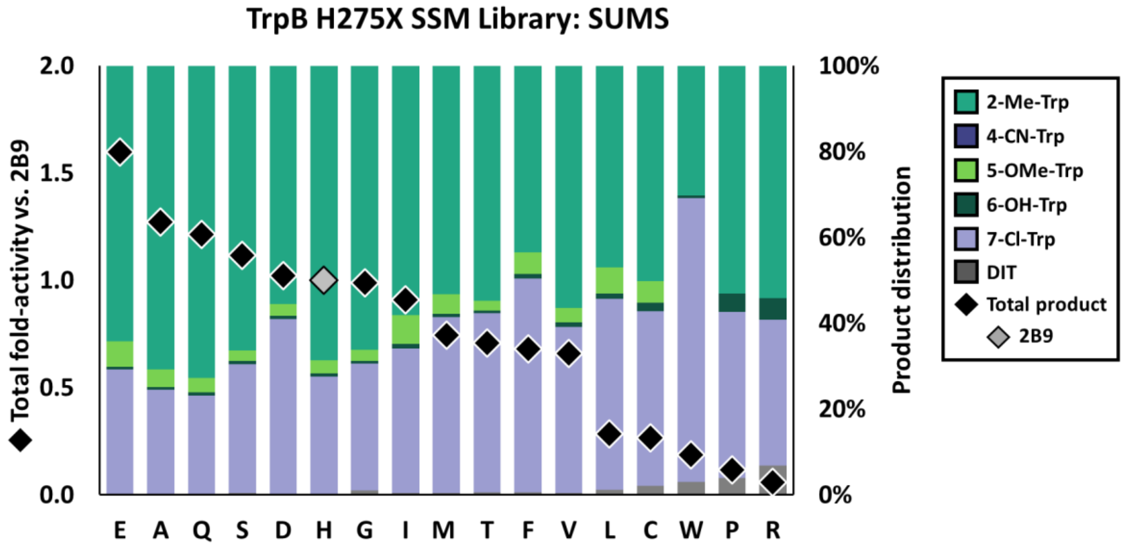

Supplementary Figure 27. SUMS results for the *Pf*TrpB H275X site-saturation mutagenesis library. Colored bars represent relative amounts of each product formed, and diamonds represent total product formed, based on ion count integrations of single-ion retention (SIR) channels. The parent (2B9) sequence is denoted by a grey diamond. Substrate panel: 5 mM each of 2-Me-indole, 4-CN-indole, 5-OMe-indole, 6-OH-indole, and indoline, and 2.5 mM 7-Cl-indole. No 4-CN-Trp product was detected. No lysine, asparagine, or tyrosine mutations were sequenced from this library plate. The sampling rate of each mutation is detailed in Supplementary Table 4.

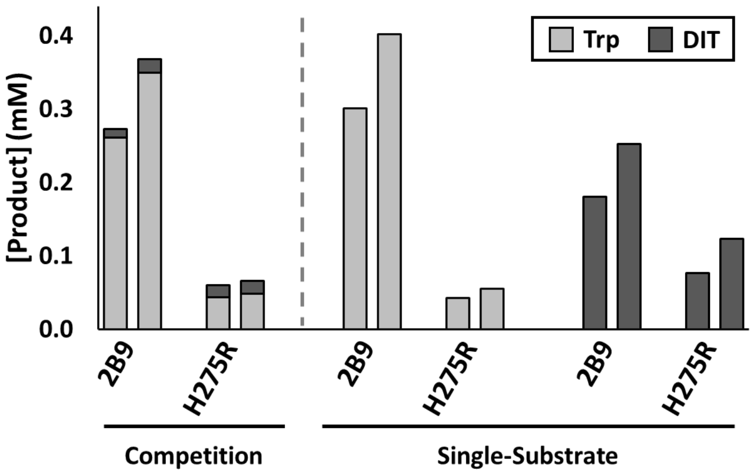

Supplementary Figure 28. Comparison of Trp and DIT production under competition and single-substrate reaction conditions. Reactions consisted of 1.5 mM indole and/or 15 mM indoline, 30 mM Ser, and 5 µM *Pf*TrpB in 100 mM potassium phosphate buffer (pH = 8.0) and 10% DMSO at 37 °C. Reactions were run in duplicate (both sets of results shown above.)

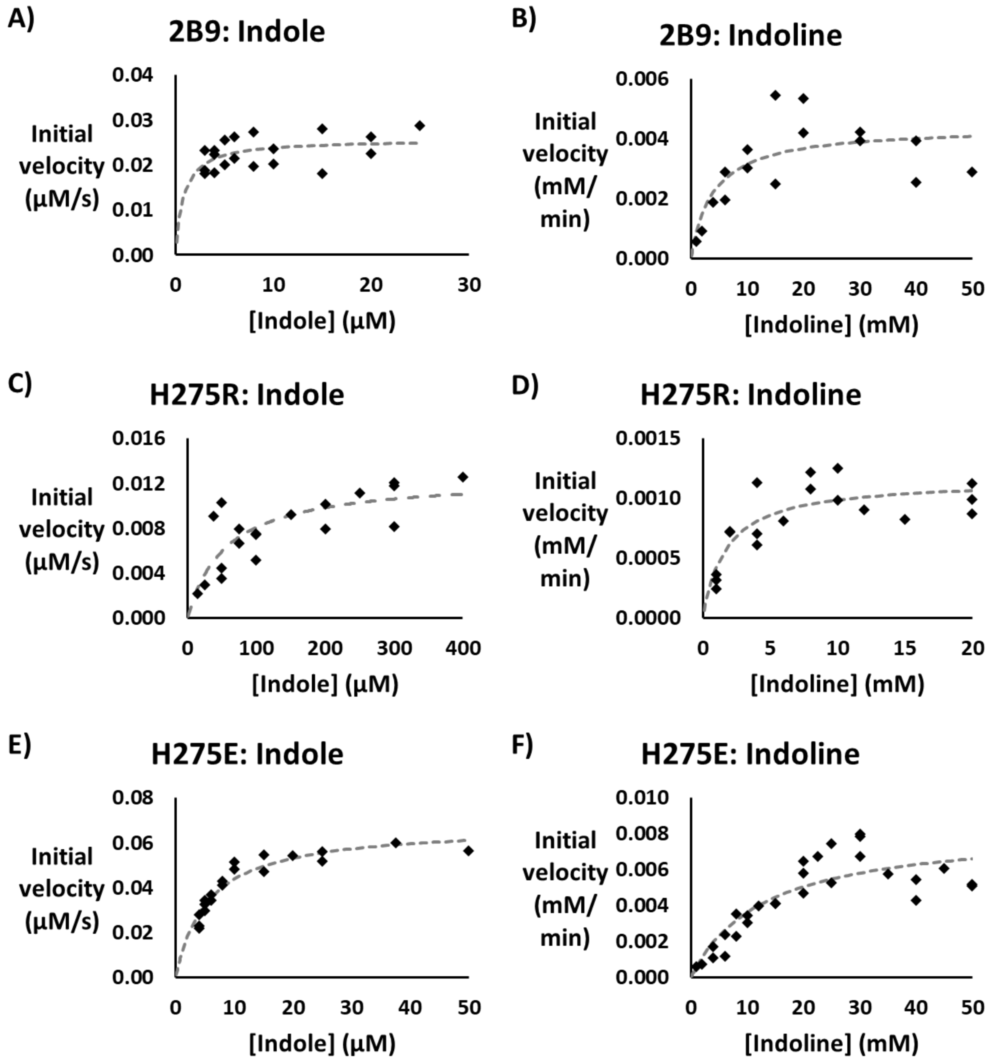

Nucleophile	Variant	k_{cat} (s^{-1})	K_M (mM)	k_{cat}/K_M ($M^{-1} s^{-1}$)
Indole	**2B9**	0.073 ± 0.005	< 0.005	> 15000
	**H275R**	0.0080 ± 0.0010	0.058 ± 0.001	144
	**H275E**	0.19 ± 0.01	< 0.010	> 19000
Indoline	**2B9**	0.042 ± 0.005	3.9 ± 0.5	11
	**H275R**	0.0061 ± 0.0005	1.7 ± 0.3	3.6
	**H275E**	0.081 ± 0.010	12.8 ± 0.3	6.4

Supplementary Figure 29. Single substrate kinetic characterization of *Pf*TrpB variants. Michaelis-Menten curves for 2B9 (**A, B**), H275R (**C, D**), and H275E (**E, F**) with indole or indoline. Enzyme concentrations used are as follows: **A)** 0.35 μM, **B)** 1.76 μM**, C)** 1.57 μM, **D)** 3.13 μM, **E)** 0.34 μM, and **F)** 1.70 μM. See SI methods for additional experimental details.

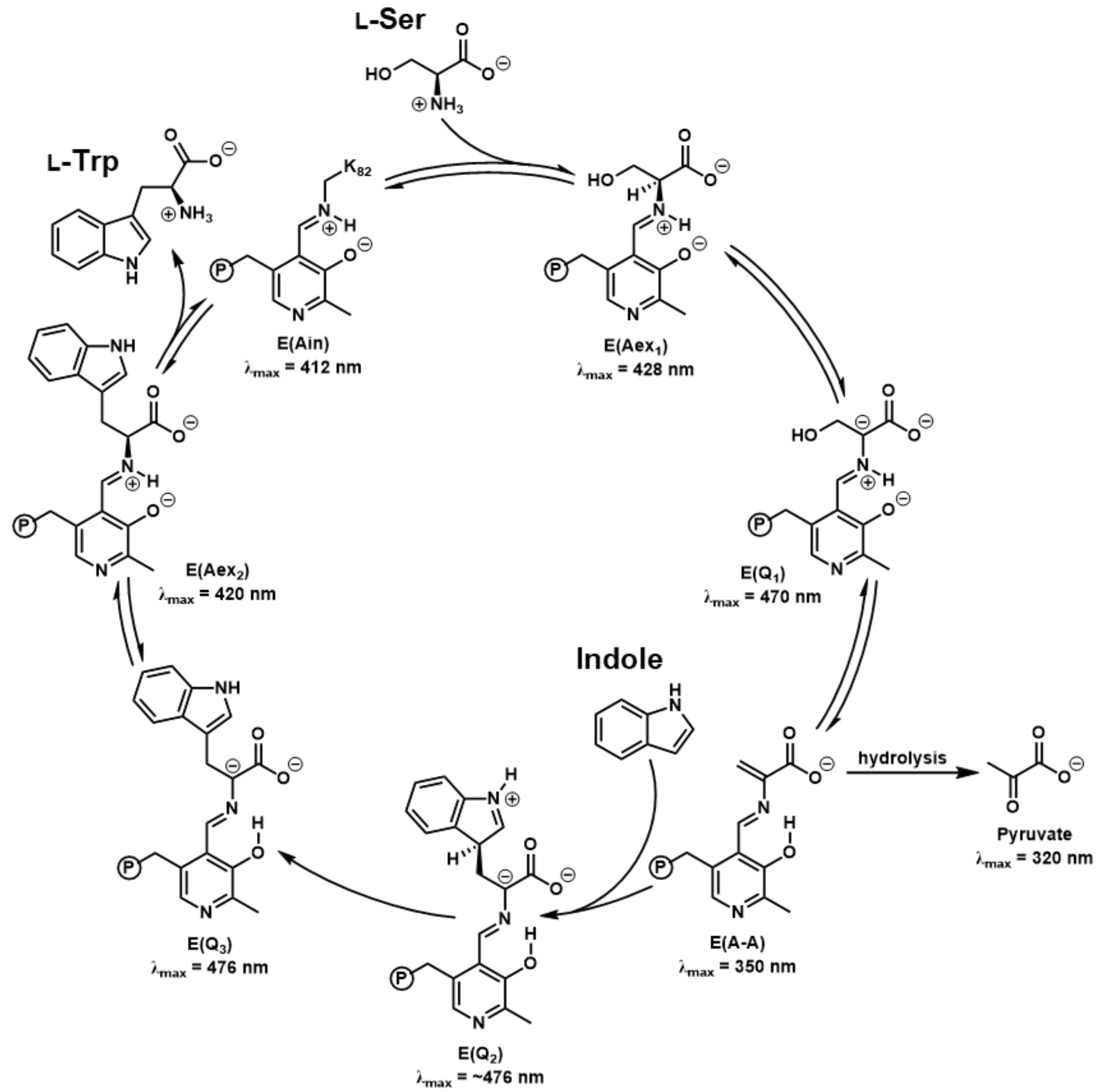

Supplementary Figure 30. Mechanism of the native reaction catalyzed by *Pf*TrpB. PLP-bound intermediates are labeled with names and λ_{max} values (from Buller et al.),[3] which enable analysis by UV-vis spectroscopy. The reactive amino-acrylate intermediate (E(A-A)) can be degraded by a hydrolysis shunt pathway that forms pyruvate.

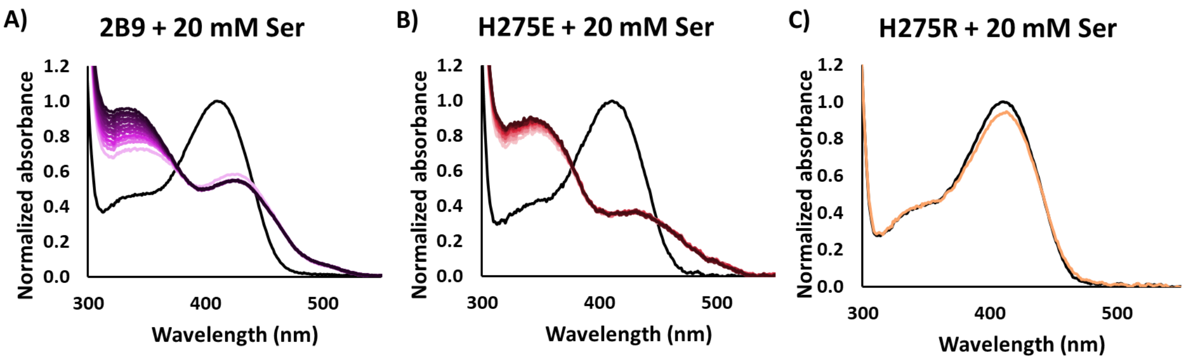

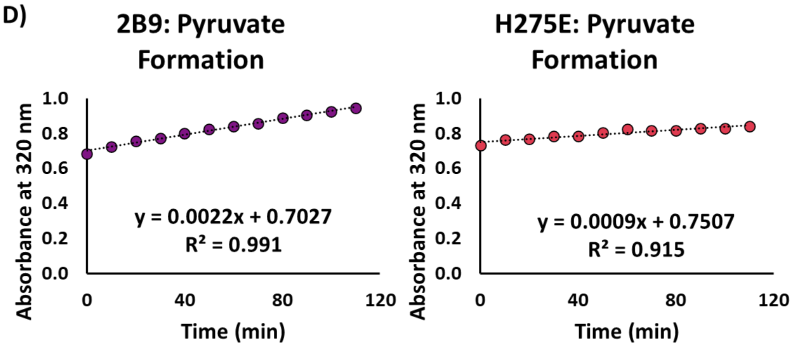

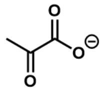

Pyruvate
ε = 20 $M^{-1}cm^{-1}$
at 320 nm

Variant	Rate of pyruvate formation (µM/s)
2B9	1.9
H275E	0.73

Supplementary Figure 31. UV-vis spectral changes upon addition of L-serine (Ser) to *Pf*TrpB variants. Enzyme only (20 µM) spectra are shown in black. **A)** For 2B9, addition of 20 mM Ser leads to formation of both external aldimine (428 nm) and amino-acrylate (350 nm) intermediates. Over time (light to dark traces), the amino-acrylate is hydrolyzed to form pyruvate (320 nm) via a shunt reaction. **B)** For H275E, addition of 20 mM Ser leads to formation of primarily amino-acrylate (350 nm). Over time (light to dark traces), the amino-acrylate intermediate is relatively stable compared to 2B9. **C)** For H275R, addition of 20 mM Ser results in only minimal formation of external aldimine (428 nm), orange trace. **D)** Comparison of rate of formation of pyruvate by 2B9 (left) and H275E (right)

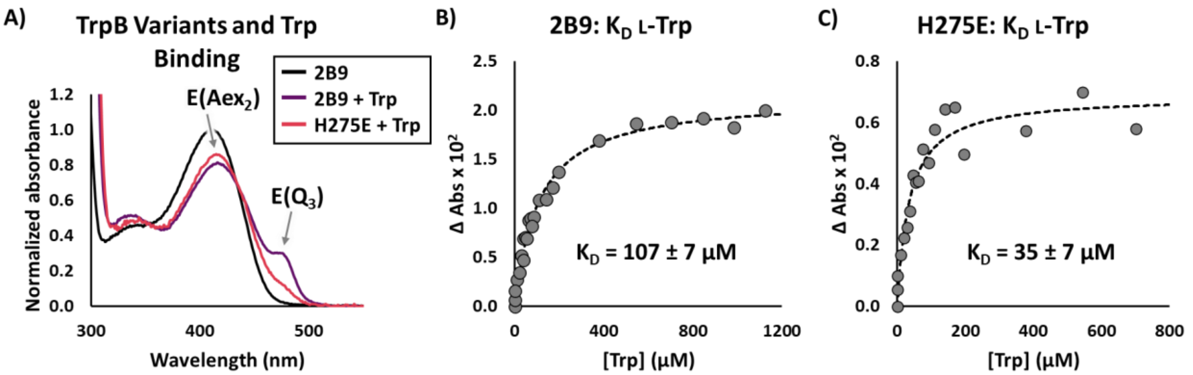

Supplementary Figure 32. Determination of K_D of L-Trp with 2B9 and H275E. A) A representative enzyme only (20 µM) spectrum is shown in black. Addition of 1 mM Trp to 2B9 (purple trace) results in formation of both external aldimine (420 nm) and quinonoid (476 nm) intermediates. Addition of 1 mM Trp to H275E (pink trace) results primarily in formation of an external aldimine Trp adduct. **B, C)** Absorbance at 475 nm, corresponding to a Trp quinonoid intermediate, was used to determine K_D with Trp. Trp was titrated into a solution of 20 µM enzyme, and resulting changes in absorbance were fit to a single-site binding model.

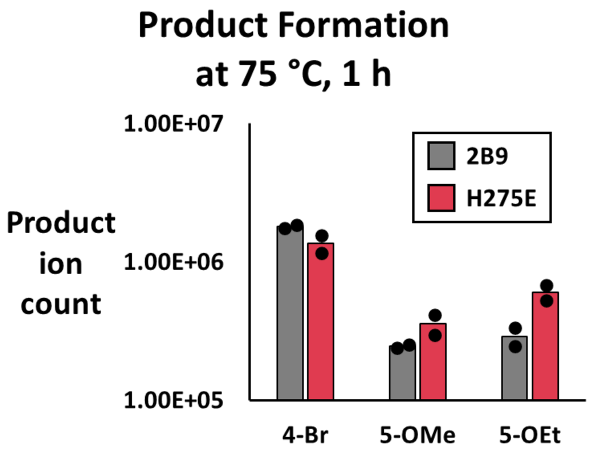

Supplementary Figure 33. The activity of H275E is generally maintained at 75 °C. Enzyme activity is reported based on ion count integrations of single-ion retention (SIR) channels (log scale). Reactions consisted of 10 mM indole substrate, 30 mM Ser, and 1 µM *Pf*TrpB in 100 mM potassium phosphate buffer (pH = 8.0) and 10% DMSO. Reactions were run in duplicate.

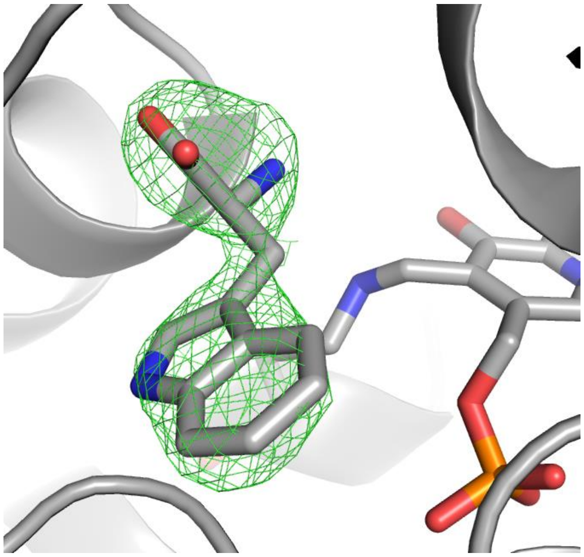

Supplementary Figure 34. Trp binding non-covalently to H275E. Fo-Fc omit map contoured at 3σ. PDB ID: 7ROF.

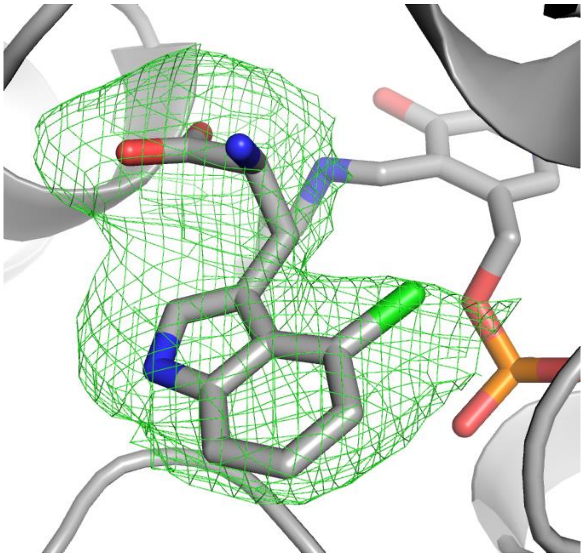

Supplementary Figure 35. 4-Cl-Trp binding non-covalently to H275E. Fo-Fc omit map contoured at 2σ. PDB ID: 7RNP.

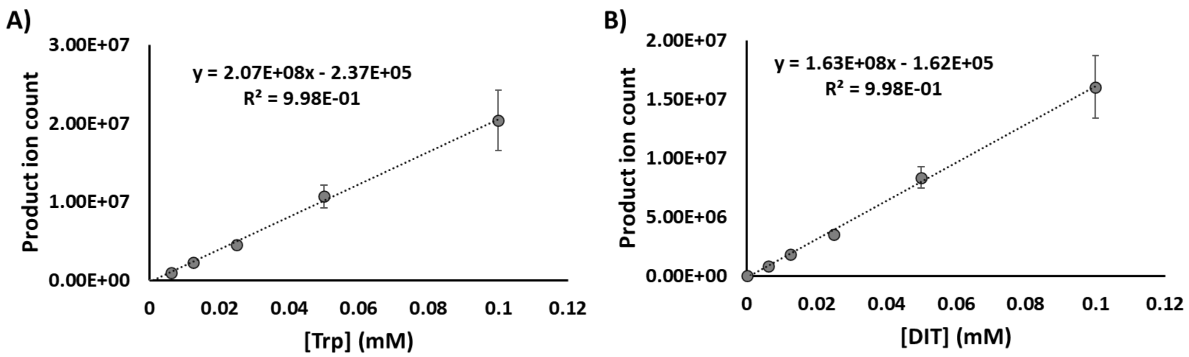

Supplementary Figure 36. Product calibration curves by mass spectrometry. Calibration curves were generated for quantification of L-tryptophan (Trp, **A**) and 2,3-dihydroisotryptophan (DIT, **B**) in multiplexed and single-substrate reactions (See Fig. 4c). Points on the standard curves represent the average of technical triplicate measurements; error bars depict the standard deviation.

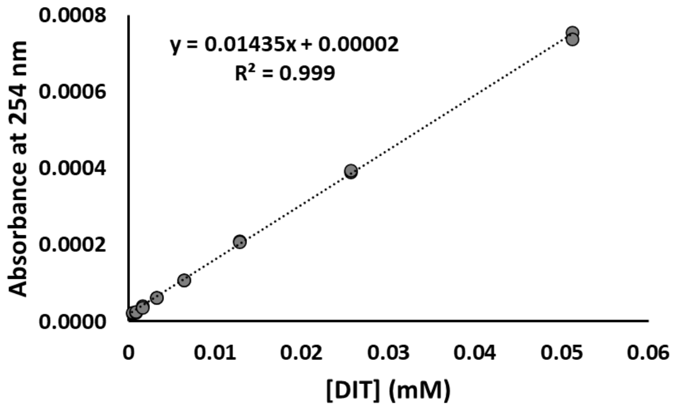

Supplementary Figure 37. Product calibration curve by absorbance at 254 nm. Calibration curve for quantification of 2,3-dihydroisotryptophan (DIT) to measure Michaelis-Menten parameters for conversion of indoline to DIT by *Pf*TrpB variants. Points on the standard curve represent the average of technical duplicate measurements.

Supplementary Tables

Supplementary Table 1. *Rgn*TDC variants kinetic parameters

Substrate	Variant	k_{cat} (s^{-1})	K_M (mM)	k_{cat}/K_M (M^{-1} s^{-1})
**Trp**	**wt TDC**	11 ± 1	1.6 ± 0.4	6900
	**F98V**	8.7 ± 1.4	2.8 ± 0.3	3100
	**W349K**	1.0 ± 0.1	1.4 ± 0.2	700
	**L355A**	0.14 ± 0.01	0.53 ± 0.45	260
	**L355M**	3.3 ± 0.3	0.54 ± 0.36	6200
**2-Me-Trp**	**wt TDC**	0.055 ± 0.008	2.0 ± 0.4	27
	**F98V**	0.042 ± 0.009	13 ± 0.3	3.3
**4-Br-Trp**	**wt TDC**	0.37 ± 0.08	3.4 ± 0.3	110
	**L355A**	0.82 ± 0.1	1.4 ± 0.2	590
	**L355M**	1.1 ± 0.1	0.7 ± 0.2	1600
**5-OMe-Trp**	**wt TDC**	0.017 ± 0.002	0.99 ± 0.48	17
	**W349K**	0.25 ± 0.03	1.1 ± 0.3	240
**6-Cl-Trp**	**wt TDC**	0.52 ± 0.02	0.14 ± 0.03	3700
	**F98V**	0.91 ± 0.06	0.60 ± 0.2	1500

Supplementary Table 2. *Rgn*TDC variant total turnover number with different substrates

	wt TDC	F98V	W349K	L355A	L355M
Trp	90,000 ± 21,000	210,000 ± 30,000	21,000 ± 3,000	3,000 ± 2,000	82,000 ± 19,000
7-I-Trp	250,000 ± 60,000	56,000 ± 11,000	19,000 ± 4,000	8,000 ± 900	160,000 ± 50,000
6-Cl-Trp	35,000 ± 9,000	120,000 ± 30,000	13,000 ± 4,000	1,000 ± 200	41,000 ± 14,000
6-NO2-Trp	10,000 ± 3,000	55,000 ± 15,000	18,000 ± 6,000	300 ± 100	14,000 ± 6,000
5-OMe-Trp	220 ± 20	160 ± 20	6,000 ± 300	60 ± 10	240 ± 20
5-OEt-Trp	90 ± 70	90 ± 10	7,000 ± 100	30 ± 5	90 ± 90
4-Br-Trp	2,200 ± 600	400 ± 300	100 ± 70	10,000 ± 2,000	20,000 ± 10,000
2-Me-Trp	1,000 ± 100	2,500 ± 700	7 ± 0	1,000 ± 100	1,000 ± 240

Supplementary Table 3. X-Ray crystallographic data collection and refinement statistics

PDB ID	7RNQ	7ROF	7RNP
Protein	*N*-His-*Pf*TrpB^{2B9}-H275E	*N*-His-*Pf*TrpB^{2B9}-H275E	*N*-His-*Pf*TrpB^{2B9}-H275E
Ligand	None	Trp	4-Cl-Trp
**Data Collection**			
Space group	$P2_12_12_1$	$P2_12_12_1$	$P2_12_12_1$
Cell dimensions (Å)	a,b,c = 57.0, 82.7, 322.6	a,b,c = 57.4, 82.7, 322.3	a,b,c = 56.2, 81.2, 321.0
Cell angles	α = β = γ = 90°	α = β = γ = 90°	α = β = γ = 90°
Wavelength (Å)	0.97618	1.033167	1.033167
Beamline	21ID-D	23ID-B	23ID-B
Resolution (Å)	40 – 2.1	40 – 2.39	40 – 2.25
Last bin (Å)	(2.14 – 2.10)	(2.45 – 2.39)	(2.30 – 2.25)
No. observations	1,199,094 (54,391)	823,988 (62,539)	935,385 (63,013)
Completeness (%)	99.5 (93.6)	99.5 (99.5)	98.8 (98.7)
R_{pim}	0.065 (0.684)	0.058 (1.548)	0.046 (0.825)
CC(1/2)	0.995 (0.818)	0.998 (0.488)	0.998 (0.703)
I/σI	11.7 (1.5)	8.4 (0.8)	8.8 (1.1)
Redundancy	13.4 (12.7)	13.4 (14.1)	13.4 (14.4)
**Refinement**			
Total no. of reflections	85150	58486	66413
Total no. of atoms	11732	11153	11224
TLS operators	40	40	40
Final bin (Å)	(2.16 – 2.10)	(2.45 – 2.39)	(2.31 – 2.25)
R_{work} (%)	21.4 (32.0)	22.7 (39.4)	22.7 (38.1)
R_{free} (%)	24.8 (36.2)	27.1 (39.7)	26.7 (39.8)
Average B factor (Å^2)	45.6	72.4	63.8
Ramachandran plot Favored, %	97.6	96.4	96.7
Allowed, %	99.7	99.8	99.7
Outliers, %	0.3	0.2	0.3

Values in parenthesis are for the highest resolution shell. R_{merge} is Σ|Io – I| / ΣIo , where Io is the intensity of an individual reflection, and I is the mean intensity for multiply recorded reflections. R_{work} is Σ||Fo – Fc|| / Fo, where Fo is an observed amplitude and Fc a calculated amplitude. R_{free} is the same statistic calculated with a 5% subset of the data that was excluded from refinement.

Supplementary Table 4. SSM library mutation sampling

	TDC Libraries						TrpB Library
**"X"**	**F98X**	**V99X**	**H120X**	**L339X**	**W349X**	**L355X**	**H275X**
**A**	1	2	0	4	5	4	3
**C**	5	2	4	3	6	8	2
**D**	4	3	1	3	2	3	3
**E**	3	4	6	1	0	3	1
**F**	**10**	3	0	2	5	3	2
**G**	3	5	5	4	3	4	3
**H**	4	3	**6**	1	1	2	**8**
**I**	1	3	3	9	2	3	3
**K**	4	5	3	2	1	3	0
**L**	6	4	2	**11**	4	**11**	6
**M**	3	8	5	5	4	3	4
**N**	6	1	4	2	0	1	0
**P**	0	5	1	2	0	0	5
**Q**	4	2	1	1	2	5	1
**R**	2	3	6	1	6	1	3
**S**	0	5	3	2	4	4	3
**T**	2	3	7	1	3	3	2
**V**	7	**10**	5	15	3	7	11
**W**	3	3	2	0	**5**	3	4
**Y**	3	2	2	2	3	4	0
**Excluded***	25	20	30	25	37	21	32
**Total**	96	96	96	96	96	96	96

*Excluded Sequences include negative and sterile controls, wells with poor sequencing reads, or (less commonly) wells with multiple mutations. Green wells with bolded numbers denote wild-type sequences.

Supplementary Table 5. DNA and primer sequences

Protein	DNA sequence (5’ to 3’)
C-His-*Pf*2B9	ATGTGGTTCGGTGAATTTGGTGGTCAGTACGTGCCAGAAACGCTG<br>GTTGGACCCCTGAAAGAGCTGGAAAAAGCTTACAAACGTTTCAAA<br>GATGACGAAGAATTCAATCGTCAGCTGAATTACTACCTGAAAACCT<br>GGGCAGGTCGTCCAACCCCACTGTACTACGCAAAACGCCTGACTG<br>AAAAAATCGGTGGTGCTAAAGTCTACCTGAAACGTGAAGACCTGG<br>TTCACGGTGGTGCACACAAGACCAACAACGCCATCGGTCAGGCAC<br>TGCTGGCAAAGCTCATGGGTAAAACTCGTCTGATCGCTGAGACCG<br>GTGCTGGTCAGCACGGCGTAGCGACTGCAATGGCTGGTGCACTGC<br>TGGGCATGAAAGTGGACATTTACATGGGTGCTGAGGACGTAGAA<br>CGTCAGAAAATGAACGTATTCCGTATGAAGCTGCTGGGTGCAAAC<br>GTAATTCCAGTTAACTCCGGTTCTCGCACCCTGAAAGACGCAATCA<br>ACGAGGCTCTGCGTGATTGGGTGGCTACTTTTGAATACACCCACTA<br>CCTAATCGGTTCCGTGGTCGGTCCACATCCGTATCCGACCATCGTT<br>CGTGATTTTCAGTCTGTTATCGGTCGTGAGGCTAAAGCGCAGATCC<br>TGGAGGCTGAAGGTCAGCTGCCAGATGTAATCGTTGCTTGTGTTG<br>GTGGTGGCTCTAACGCGATGGGTATCTTTTACCCGTTCGTGAACGA<br>CAAAAAAGTTAAGCTGGTTGGCGTTGAGGCTGGTGGTAAAGGCCT<br>GGAATCTGGTAAGCATTCCGCTAGCCTGAACGCAGGTCAGGTTGG<br>TGTGTCCCATGGCATGCTGTCCTACTTTCTGCAGGACGAAGAAGGT<br>CAGATCAAACCAAGCCACTCCATCGCACCAGGTCTGGATTATCCAG<br>GTGTTGGTCCAGAACACGCTTACCTGAAAAAAATTCAGCGTGCTG<br>AATACGTGGCTGTAACCGATGAAGAAGCACTGAAAGCGTTCCATG<br>AACTGAGCCGTACCGAAGGTATCATCCCAGCTCTGGAATCTGCGC<br>ATGCTGTGGCTTACGCTATGAAACTGGCTAAGGAAATGTCTCGTG<br>ATGAGATCATCATCGTAAACCTGTCTGGTCGTGGTGACAAAGACCT<br>GGATATTGTCCTGAAAGCGTCTGGCAACGTGCTCGAGCACCATCA<br>CCATCACCATTGA
C-His-*Rgn*TDC	ATGTCGCAGGTCATTAAGAAAAAACGCAATACGTTTATGATTGGAACGG<br>AGTACATCCTTAATTCGACACAGTTAGAGGAGGCAATTAAGTCTTTCGTG<br>CACGATTTTTGTGCGGAAAAACATGAGATCCATGATCAGCCCGTCGTTGT<br>TGAAGCCAAGGAGCACCAGGAAGATAAAATTAAGCAGATCAAGATCCCT<br>GAAAAAGGACGCCCAGTAAATGAGGTCGTGAGTGAGATGATGAATGAA<br>GTTTACCGCTATCGCGGAGATGCGAACCACCCCCGTTTCTTCTCCTTCGTT<br>CCGGGTCCAGCTTCGAGCGTCTCCTGGCTTGGAGACATCATGACGAGTG<br>CATATAATATCCATGCCGGAGGCAGTAAATTGGCTCCCATGGTAAACTGT<br>ATTGAGCAAGAAGTGCTGAAGTGGTTGGCAAAGCAAGTGGGATTTACTG<br>AAAATCCCGGCGGGGTGTTCGTCTCAGGTGGCTCGATGGCGAACATCAC<br>GGCGTTAACAGCAGCCCGTGACAATAAACTTACTGACATTAATTTGCATT<br>TAGGAACGGCGTATATCAGCGACCAAACACACTCCAGTGTAGCCAAGGG<br>GTTACGTATTATTGGCATCACCGACAGCCGTATTCGCCGTATTCCCACTAA

	TTCGCACTTCCAAATGGATACGACCAAGTTGGAGGAGGCCATTGAAACC<br>GATAAAAAGAGTGGCTATATCCCGTTTGTAGTGATCGGAACCGCTGGCA<br>CGACTAATACAGGATCCATTGACCCATTAACGGAAATTTCTGCATTATGT<br>AAAAAGCACGATATGTGGTTCCACATCGACGGTGCGTATGGTGCCTCCG<br>TATTGCTTAGTCCAAAATATAAGTCCCTTTTGACAGGAACAGGATTAGCA<br>GATAGTATTTCTTGGGATGCTCACAAATGGTTATTCCAGACGTATGGGTG<br>CGCCATGGTATTGGTGAAGGACATCCGCAACCTGTTCCATTCCTTTCACG<br>TTAACCCCGAATATCTGAAAGACCTTGAGAATGACATTGATAATGTCAAT<br>ACGTGGGATATTGGGATGGAGTTAACACGTCCGGCACGTGGGCTTAAAC<br>TTTGGCTGACCTTGCAGGTTCTTGGGTCCGACCTTATTGGGTCTGCAATT<br>GAGCACGGTTTCCAATTAGCGGTATGGGCGGAAGAAGCGCTGAATCCCA<br>AAAAAGATTGGGAAATTGTTAGCCCTGCCCAGATGGCGATGATTAATTTT<br>CGCTACGCGCCTAAGGATTTAACCAAAGAGGAGCAGGACATCCTTAATG<br>AAAAGATTTCGCATCGCATCTTGGAATCAGGCTATGCCGCTATTTTTACTA<br>CTGTGCTGAATGGTAAGACAGTGTTACGCATTTGCGCGATTCACCCTGAG<br>GCTACTCAAGAGGATATGCAGCACACCATTGATCTGTTGGACCAATACG<br>GTCGCGAGATCTATACTGAAATGAAAAAGGCTCTCGAGCACCATCACCAT<br>CACCATTGA
His275_XXX_F<br>(2B9)	GAA CGC AGG TCA GGT TGG TGT GTC CXX XGG CAT GCT GTC C
His275_R<br>(2B9)	CAC CAA CCT GAC CTG CGT TCA GGC TAG CGG
Phe98_XXX_F<br>(*Rgn*TDC)	GAA CCA CCC CCG TTT CTT CTC CXX XGT TCC GGG TCC AG
Phe98_R<br>(*Rgn*TDC)	GAA ACG GGG GTG GTT CGC ATC
His120_XXX_F<br>(*Rgn*TDC)	GAC ATC ATG ACG AGT GCA TAT AAT ATC NDT GCC GGA GGC AG
His120_R<br>(*Rgn*TDC)	GCA CTC GTC ATG ATG TCT CCA AGC C
Leu126_XXX_F<br>(*Rgn*TDC)	TAA TAT CCA TGC CGG AGG CAG TAA AXX XGC TCC CAT GGT AA
Leu126_R<br>(*Rgn*TDC)	GCC TCC GGC ATG GAT ATT ATA TGC AC
Leu336_XXX_F<br>(*Rgn*TDC)	CAT TCC TTT CAC GTT AAC CCC GAA TAT XXX AAA GAC CTT GAG
Leu336_R<br>(*Rgn*TDC)	CGG GGT TAA CGT GAA AGG AAT G
Leu339_XXX_F<br>(*Rgn*TDC)	ACC CCG AAT ATC TGA AAG ACX XXG AGA ATG ACA TTG
Leu339_R<br>(*Rgn*TDC)	CTT TCA GAT ATT CGG GGT TAA C
Trp349_XXX_F<br>(*Rgn*TDC)	GAC ATT GAT AAT GTC AAT ACG XXX GAT ATT GGG ATG GAG
Trp349_R	CGT ATT GAC ATT ATC AAT GTC ATT CTC AAG G

(*Rgn*TDC)	
Leu355_XXX_F (*Rgn*TDC)	GGA TAT TGG GAT GGA GXX XAC ACG TCC GGC AC
Leu355_R (*Rgn*TDC)	CAT CCC AAT ATC CCA CGT ATT GAC
Thr356_XXX_F (*Rgn*TDC)	GAT ATT GGG ATG GAG TTA XXX CGT CCG GCA CGT
Thr356_R (*Rgn*TDC)	CTC CAT CCC AAT ATC CCA CGT ATT GAC

All primers were purchased from Integrated DNA Technologies. XXX indicates a 22-codon library made as a mixture of 3 degenerate codon primers (NDT, VHG, TGG), as described by Kille, S. et al.[4]

DNA Isolation and Storage: DNA was purified via gel electrophoresis and isolated using a DNA gel extraction kit (Zymo Research). All isolated DNA was stored at -20 ^{o}C.

Supplementary Discussion

Introduction to substrate competition kinetics behind SUMS

We provide here a brief discussion on the underlying kinetics for multiplexed reactions as an initial guide for understanding substrate competition kinetics in the context of SUMS for protein engineering, particularly when increased activity on single substrates is desired. We note that Stanišić et al. 2019 provide an excellent discussion on substrate competition kinetics.[5] We also found Chou and Talalay 1977, Cornish-Bowden 1984, and Andrews 2016 to be particularly useful references.[6–8] While detailed understanding of substrate competition kinetics is not required to implement SUMS, we suggest that consideration of the impact of different kinetic effects on screening results will aid attempts to engineer synthetically useful enzymes.

1. **Substrate competition kinetic background**

Starting from the traditional Michaelis-Menten equation (Eq. 1):

$$v_0 = \frac{k_{cat} * [E_T] * [S]}{K_M + [S]} \quad , \qquad \textbf{Eq. 1}$$

we use the familiar definitions v_0 = initial velocity, $[E_T]$ = total enzyme concentration, $[S]$ = concentration of substrate, and K_M = Michaelis-Menten constant. For a unimolecular, irreversible reaction (Eq. 2), if $k_2 << k_1, k_{-1}$, we can approximate $k_{cat} = k_2$.

$$E + S \underset{k_{-1}}{\overset{k_1}{\rightleftharpoons}} ES \xrightarrow{k_2} E + P \qquad \textbf{Eq. 2}$$

In this simplified model, K_M can be approximated as a function of the rate constants involved in the formation/breakdown of the ES complex:

$$K_M = \frac{k_{-1} + k_2}{k_1} \qquad \textbf{Eq. 3}$$

In the case of single substrate reactions where $[S] >> K_M$, the term $k_{cat} * [E_T] = V_{max}$ becomes the main determinant of the rate of product formation. We show below that this familiar kinetic phenomenon no longer holds for reactions where multiple substrates are present.

We found it instructive to consider substrate competition through the lens of competitive inhibition. A competitive inhibitor decreases overall enzymatic activity by binding in the enzyme active site, decreasing the availability of enzyme active sites that can catalyze the given transformation on substrate *S* (Eq. 4).

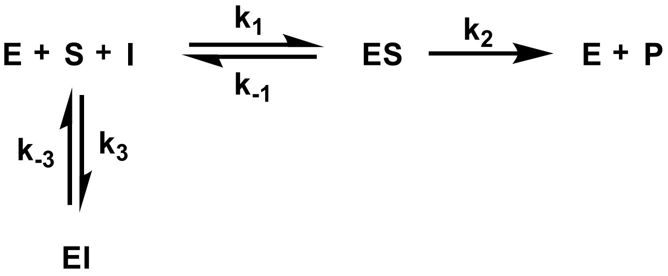

Eq. 4

The impact of inhibitor *I* with an inhibition constant K_I on the rate of an enzymatic reaction can be calculated as:

$$v_0 = \frac{k_{cat} * [E_T] * [S]}{K_M\left(1 + \frac{[I]}{[K_I]}\right) + [S]}$$

Eq. 5

where:

$$K_I = \frac{k_{-3}}{k_3}.$$

Eq. 6

In a multiplexed reaction, substrates will mutually inhibit each another by competing for active site binding in a fashion analogous to competitive inhibition. Given the simple model below of S_A and S_X competing to form products P_A and P_X (Eq. 7), respectively, the additional rate constant k_4 for the formation of P_X is the only distinction between this model and the previous competitive inhibition model (Eq. 4).

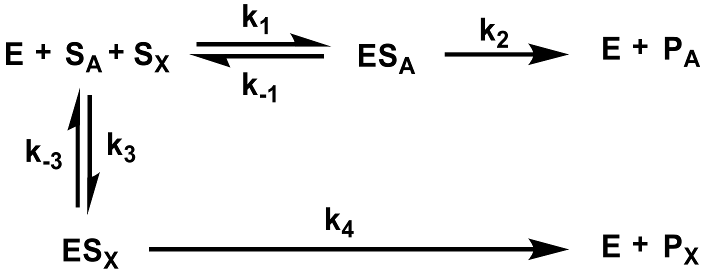

Eq. 7

S_X becomes a classical competitive inhibitor when $k_4 = 0$, where $K_{MX} = K_I$. This means K_{MX} can be defined as the Michaelis-Menten constant for a given S_X and describes the inhibition of S_X on S_A, as shown in Eq. 8.

$$v_{A0} = \frac{k_{catA} * [E_T] * [S_A]}{K_{MA}\left(1 + \frac{[S_X]}{[K_{MX}]}\right) + [S_A]}$$

Eq. 8

Therefore, for any given number of additional substrates, the initial velocity of formation of P_A can be described as:

$$v_{A0} = \frac{k_{catA} * [E_T] * [S_A]}{K_{MA}\left(1 + \frac{[S_B]}{[K_{MB}]} + \cdots \frac{[S_Z]}{[K_{MZ}]}\right) + [S_A]}$$

Eq. 9

where the sum of the ratios of $[S_X]$ and K_{MX} models the present substrate competition and inhibition in a substrate multiplexed reaction. Therefore, even when $[S_X] >> K_{MX}$ for all substrates, substrate K_M's still have a significant impact on relative product distribution.

2. Supporting data for substrate competition kinetic model

Many synthetic applications of enzymes use single-substrate reaction conditions. In the context of single-substrate reactions with high substrate concentrations, k_{cat} is an excellent predictor of relative activity, as measured by observed total turnover numbers for different chlorinated tryptophan analogs (Supplementary Fig. 38a, data from Ref 2). Catalytic efficiency, k_{cat}/K_M, is an ineffective predictor of single-substrate activity (Supplementary Fig. 38b).

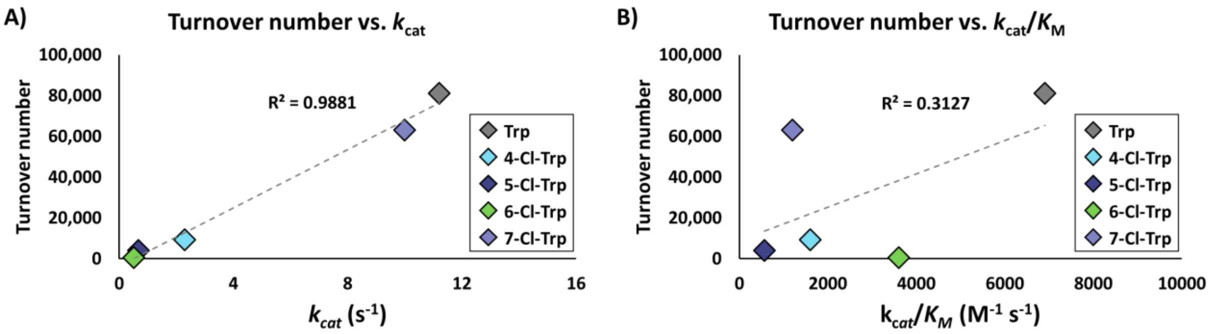

Supplementary Figure 38. Kinetic parameters and activity on single substrates. Comparison of single-substrate activity correlation with **(a)** k_{cat} or **(b)** k_{cat}/K_M. Turnover numbers and Michaelis-Menten parameters from McDonald et al. 2019.[2]

Conversely, in a reaction where substrates compete for an enzyme active site, the ratio of the substrates' k_{cat}/K_M values can be used to predict relative product abundance. From an example three-substrate multiplexed reaction of *Rgn*TDC, we show that the relative ratios of the three products do not correlate well to the substrates' relative k_{cat}'s (Supplementary Fig. 39a) but correlate very well with substrate k_{cat}/K_M's, as determined from single-substrate kinetic measurements (Supplementary Fig. 39b). Notably, the K_M values for Trp, 4-Br-Trp, and 6-Cl-Trp with *Rgn*TDC are 1.6 mM, 3.4 mM, and 0.14 mM, respectively. The reaction depicted in Supplementary Fig. 39b was conducted with 5 mM of each Trp analog, demonstrating that product profiles reflect the ratio of each substrate's catalytic efficiency, even at concentrations that exceed a substrate's K_M. SUMS methods are compatible with high substrate concentrations, which are typically desirable for synthetic applications.

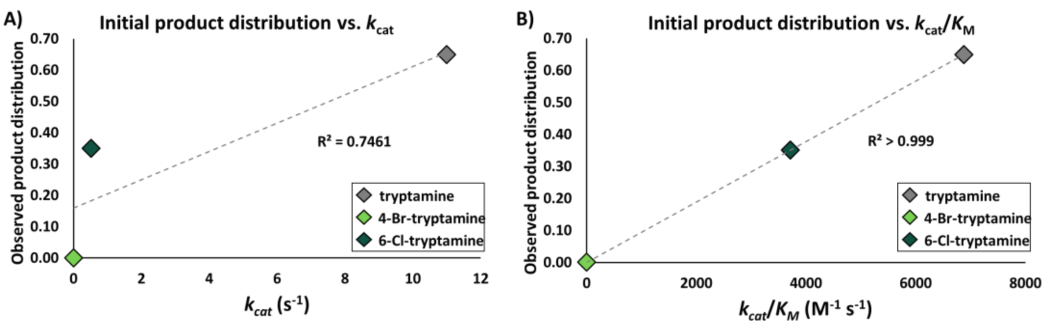

Supplementary Figure 39. Kinetic parameters and activity on competing substrates. Observed product percentage from a multiplexed reaction correlated with **(a)** k_{cat} or **(b)** k_{cat}/K_M. (Reaction conditions: 1 μM *Rgn*TDC, 10 μM pyridoxal phosphate, 5 mM each substrate: Trp, 6-Cl-Trp, 4-Br-Trp, 5 min at 37 °C.) Michaelis-Menten parameters from McDonald et al. 2019 and Supplementary Table 1.[2]

3. Repression of inhibition

During library screening, some variants may acquire mutations that increase the abundance of one or more products. Several distinct kinetic scenarios can provide this outcome. In the simplest case, the mutations may increase the k_{cat} with a substrate, leading to more product formed. Alternatively, mutations may leave k_{cat} minimally changed but selectively decrease the K_M with a substrate, making it a more effective competitive inhibitor in a multiplexed setting and leading to increased occupancy of the available active sites. Both kinetic changes provide a classically activated enzyme, where mutation increases the catalytic efficiency, k_{cat}/K_M.

However, there is an alternative scenario that may arise in substrate multiplexed settings where the relative abundance of a product increases, but there is either no change or a *decrease* in the catalytic efficiency of the enzyme. This phenomenon arises when two (or more) substrates in competition react with significantly different catalytic efficiencies. As has been described above, the substrate with the higher catalytic efficiency will dominate the enzyme active site. If a mutation decreases the catalytic efficiency for both substrates but is much more deleterious with the originally preferred substrate, then the occupancy of the active site in competition will shift relative to the parent enzyme to favor the less-reactive substrate. *In effect, the ability of a 'good' substrate to inhibit reaction with 'bad' substrates is diminished*. Consequently, the relative amount of product formed from a poorly active substrate can increase. We describe this phenomenon as activation through "repression of inhibition." Notably, the apparent increases in relative activity caused by repression of inhibition detected by a multiplexed screen do not translate to single substrate conditions, since there has been no improvement in activity with the poor substrate. This phenotype is reproducible, and not an indication of errors or false positives in the screening process.

To help illustrate this subtle concept, we have generated a hypothetical scenario in Supplementary Fig. 40. The hypothetical Parent enzyme strongly favors formation of product B in a multiplexed setting. For Variant 1, the K_M for substrate B has increased 100-fold, suppressing its ability to inhibit substrate A. Consequently, Variant 1 produces far more product A than the Parent in the multiplexed assay, as determined by the relative k_{cat}/K_M values for each substrate-enzyme pair. However, parent and variant 1 would form product A at the same rate in a single-substrate reaction, since both enzymes have the same $\underline{k_{cat}}$ and K_M for substrate A.

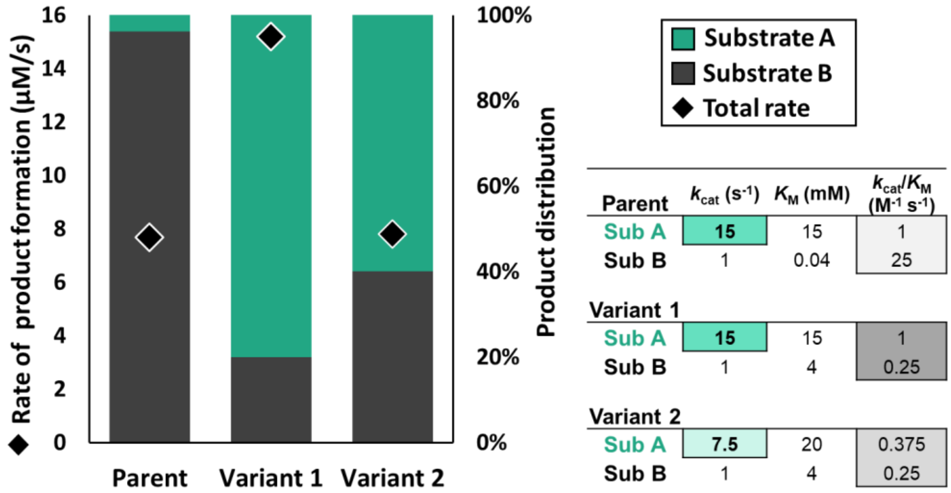

Parent	k_{cat} (s^{-1})	K_M (mM)	k_{cat}/K_M (M^{-1} s^{-1})
Sub A	15	15	1
Sub B	1	0.04	25
**Variant 1**			
Sub A	15	15	1
Sub B	1	4	0.25
**Variant 2**			
Sub A	7.5	20	0.375
Sub B	1	4	0.25

Supplementary Figure 40. Simulation of repression of inhibition. Simulated SUMS results are shown for a parent enzyme and two hypothetical variants (left). Simulation conditions were as follows: 3.3 mM each substrate and 7.5 µM enzyme. Relative bar heights represent rates of initial product formation as calculated by Equation 6. Corresponding simulated kinetic parameters are shown in the table (right). Product ratios in competition are governed by the ratio of the substrates' k_{cat}/K_M, and relative activity in single-substrate reactions is determined by k_{cat}.

In the more complex case of Variant 2, the K_M for substrate B has again increased 100-fold, suppressing its ability to inhibit substrate A. Along with this change, we considered a decrease in k_{cat} and increase in K_M for substrate A. In the multiplexed assay, Variant 2 will generate more product from substrate A. However, single-substrate reactions will reveal that Variant 2 actually has *decreased* catalytic efficiency with substrate A relative to Parent. At first glance, single-substrate and multiplexed results may appear to contradict one another, but we contend through the examples shown here that the repression of inhibition phenotype can be rationalized using standard kinetic analysis.

We have not yet determined a simple means for predicting whether improvements in activity identified by SUMS are due to an increase in catalytic efficiency or merely repression of inhibition. However, we observe this phenotype most frequently when

screening on substrates that have significant differences in their relative reactivity. During the assay design phase of SUMS (detailed below), one can choose substrates with similar reactivity to limit the impact of the repression of inhibition phenomenon. Ultimately, validation of potential hits is a core step in protein engineering, regardless of whether single-substrate screening or SUMS was used. We assert that variants that demonstrate a shift in product profile (such as the TrpB variant H275R, which showed a repression of inhibition phenotype) can point to mutations that influence substrate specificity and are therefore interesting candidates for further investigation.

Guide to optimization of SUMS method

We provide here a discussion on possible optimization routes for fine-tuning a substrate multiplexed screen. We acknowledge there are additional options for optimization but will highlight ones we felt had the greatest impact on our development of SUMS for protein engineering.

1. Choice of substrates

While there is no "right" or "wrong" panel of substrates, the substrates chosen for SUMS will directly impact the information gained from screening. This list is not intended to be comprehensive, and any substrate panels should be tested with parent enzyme to confirm that all desired products can be resolved as anticipated.

a. Number of substrates

In theory, any number of substrates can be added to a multiplexed substrate mixture. We chose <10 substrates for the reported screens for simplicity and to ease interpretation of the data. Additional substrates may be considered according to the time constraints of the researcher, the degree of mutual substrate inhibition with the selected substrates, and any potential instrumental limitations.

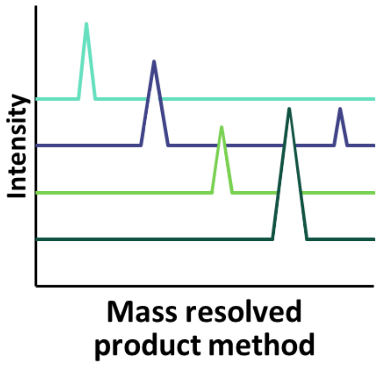

Supplementary Figure 41. SUMS output example with five substrates at four unique masses.

b. *Substrate/product masses*

The implementation of SUMS described here leveraged MS-based peak integration to quantitate distinct products. Although chromatographic resolution of products of the same mass could work in a SUMS setting (Supplementary Fig. 41), we chose substrates that will yield products with unique m/z's.

c. *Relative substrate activity*

Since highly active substrates can inhibit the formation of products from less active substrates, the addition of both highly active and inactive substrates to a substrate mixture may decrease the amount of information obtained from a screen. *In principle, a SUMS setup will have maximum sensitivity when each of the substrates is a relatively poor substrate for the native enzyme, such that none is a good competitive inhibitor and there is a large potential dynamic range of improved activities.* However, differences in baseline activity do not prevent successful application of SUMS. Substrates with which the parent enzyme has no detectable activity can be added to the screening mixture to monitor for variants that gain new activity. The presence of more reactive substrates may obscure low levels of activity that arise, but modulation of relative substrate concentrations can account for differences in activities. For both *Rgn*TDC and TrpB engineering, the concentration of the most highly active substrate(s) was reduced relative to the concentration of other substrates.

2. Screening time

For a single-substrate screen to be sensitive to improvements in activity, the parent enzyme must not reach full conversion of substrate to product. However, in a multiplexed screen, the reaction rate for different substrates may vary greatly. It is possible that some substrates may reach high or even full conversion while others show only trace activity. One may thus choose conditions under which all desired products are observed, possibly resulting in full consumption of certain substrates. As reactions depart the initial velocity regime, the relative product ratios will shift away from the ratios of substrates' k_{cat}/K_M values, as shown in Supplementary Fig. 42a. We therefore describe the distribution of products as the *SUMS promiscuity profile* rather than a specificity profile, since relative rates of product formation are not necessarily assessed. In this way, the screen can identify variants that both retain existing activity and improve activity on poor substrates.

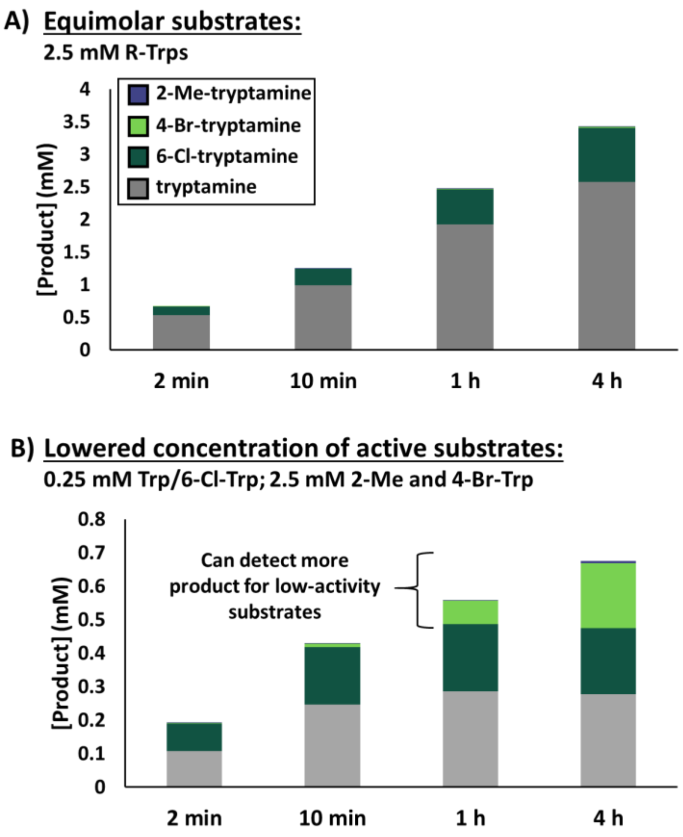

Supplementary Figure 42. Timecourse of a substrate multiplexed reaction of wild-type *Rgn*TDC with 2-methyltryptophan, 4-bromotryptophan, 6-chlorotryptophan, and tryptophan. Reactions run in triplicate, and graphed data indicates the average [product] between replicates. **a)** 2.5 mM each R-Trp, 0.25 μM TDC. **b)** 2.5 mM 2-Me-Trp and 4-Br-Trp, 0.25 mM Trp and 6-Cl-Trp, 0.25 μM TDC.

3. Relative substrate concentrations

a. Unimolecular reactions

To help offset the impact of mutual substrate inhibition, the relative concentrations of the various substrates can be adjusted. As shown in Supplementary Fig. 42b, the inhibitory capacity of highly reactive substrates can be reduced by decreasing their concentration. Such an approach increases the abundance of poorer products (Supplementary Fig. 42b, ~10-fold increase in total product formed for 4-Br-Trp and 2-Me-Trp) while maintaining an activity threshold for the substrates with high baseline activity.

b. Higher order reactions

For enzymes that catalyze bimolecular and higher order reactions, early timepoint product distributions can be ‘captured’ by limiting the stoichiometry of a non-multiplexed substrate Knorrscheidt et al. 2021 describe such an approach with limiting concentrations of H_2O_2 for their peroxygenase reactions.[9] We chose to use a limiting amount of L-serine (Ser) in the bimolecular reaction catalyzed by TrpB to ensure that tryptophan production stalled with respect to indole consumption. In this way, optimization of reaction time and catalyst loading was simplified. Alternatively, an excess of Ser could have been used to increase assay sensitivity to low abundance products.

4. Hit Identification

Unlike in single-substrate screens, where identifying the variant with the highest activity is the singular goal, *in SUMS there may be multiple classifications of a ‘hit’ or a variant that merits further investigation***.** Here, we will discuss briefly how we classified hits in our engineering campaigns of *Rgn*TDC and TrpB-2B9.

For *Rgn*TDC engineering, we looked primarily for two types hits from screening: **1) variants with boosts in total activity** and **2) variants with a boost in activity on at least one individual substrate in the screen**. As we did not find any generally activating mutations, the hits we investigated fell primarily under the second class of hits. Variants such as W349K and L355A showed enhanced activity with previously low-activity Trp analogs, and thus were further investigated.

We took a different approach to TrpB engineering, as we were no longer looking in the active site, where we could expect to easily observe drastic changes in activity and specificity. Therefore, along with the two previously described hit classifications, we looked for an additional type of hit: **3) variants with shifts in the product distribution** (regardless of whether activity for any substrate(s) increases or decreases). We surmised that such shifts would be indicative of mutations that are involved with substrate discrimination and therefore are likely influencing the enzyme active site. During screening, we found the generally activated I102T, which falls under the first hit classification, and H275R, which decreased total activity but shifted the product profile, falling under the third hit classification.

Supplementary Methods

General experimental methods

Chemicals and reagents were purchased from commercial suppliers (Sigma-Aldrich, VWR, Chem-Impex International, Alfa Aesar) and used without further purification. *E. coli* (BL21 (DE3)) cells were used for protein expression. An Eppendorf E-porator (2500 V) was used for transformations. New Brunswick I26R, 120 V/60 Hz shakers (Eppendorf) were used for cell growth. Cell disruption via sonication was performed with a Sonic Dismembrator 550 (Fisher Scientific) sonicator. Optical density measurements were collected on a UV-2600 Shimadzu spectrophotometer (Shimadzu). UPLC-MS data were collected on an Acquity UHPLC with an Acquity QDA MS detector (Waters). Column separations were performed on an Isolera One Flash Purification system (Biotage). NMR data were collected on a Bruker 500 MHz spectrometer. High resolution mass data were collected with a Q Extractive Plus Orbitrap (NIH 1S10OD020022-1) instrument with the samples ionized by ESI. Kinetic data were fit with PRISM 8 Graphpad software.

Cloning and expression of *Rgn*TDC site-saturation libraries

A codon-optimized copy of the *Ruminococcus gnavus* tryptophan decarboxylase (*Rgn*TDC) gene with a C-terminal His-tag was previously purchased[2] as a gBlock from Integrated DNA Technologies. This DNA fragment was inserted into a pET22b vector via Gibson assembly.[10] BL21 *E. coli* cells were subsequently transformed with the resulting cyclized DNA product *via* electroporation. After 30 min of recovery in Luria-Burtani (LB) media at 37 °C, cells were plated onto LB plates with 100 µg/mL ampicillin (AMP) and incubated overnight. Single colonies were used to inoculate 5 mL TB + 100 µg/mL AMP and grown overnight at 37 °C. pET22b-*Rgn*TDC plasmid was purified via the Zymo Research Plasmid Prep kit and eluted in 20 µL sterile Milli-Q H_2O. Plasmids were stored at -20 °C.

Based on a previously reported model of an *Rgn*TDC-Trp complex (PDB ID: 4OBV),[1,2] active site residues in the *Rgn*TDC active site were chosen for mutagenesis. The sites were: Phe98, Val99, His120, Leu126, Leu336, Leu339, Trp349, Leu355, and Thr356 (Supplementary Fig. 5). Primers were purchased from Integrated DNA Technologies. For each site of mutation, three primers encoding the degenerate codons NDT, VHG, and TGG at the codon of interest were mixed in a 12:9:1 ratio, respectively.[4]

Each gene library was amplified first as two separate fragments and then combined via polymerase chain assembly (PCA)[11] to form full-length *Rgn*TDC gene mutagenized at the site of interest. The corresponding genes were then inserted into a pET22b vector as described above and then transformed into BL21(DE3) *E. coli* cells and plated on LB + 100 µg/mL AMP agar plates.

Individual colonies were picked and used to inoculate wells containing 600 µl TB + 100 µg/mL AMP in a 96-well plate. One column of the plate was used as an internal control with five wild-type colonies, one sterile control, and two non-*Rgn*TDC pET22b negative controls. Plates were grown at 37 °C for 16 h at 200 RPMs. 20 µl of this starter culture plate was used to inoculate 630 µl TB + 100 µg/mL AMP. These expression plates were grown at 37 °C for 3 h until OD_{600} ~ 1.0. Plates were then placed on ice for 1 h. Expression was induced by addition of 50 µl TB + 100 µg/mL AMP + 14 mM IPTG + 7 mM indole. Plates were then grown at 23 °C for 16 h at 200 RPM and subsequently spun down at 4000 x*g* for 15 min and the supernatant discarded. Plates containing cell pellets were stored at -20 °C.

Screening of *Rgn*TDC site-saturation libraries

Cell pellets were thawed and then resuspended in lysis buffer: 50 mM potassium phosphate buffer (pH = 8.0), 1 mg/mL Hen Egg White Lysozyme (GoldBio), 0.2 mg/mL DNaseI (GoldBio), 1 mM $MgCl_2$, and 300 µM pyridoxal 5′-phosphate (PLP). A volume of 600 µL lysis buffer per well was used. After 45 min of shaking at 37 °C, the resulting lysate was then spun down at 4000 x*g* to pellet cell debris. Then, 180 µL of the resulting supernatant was added to 20 µL of a substrate mixture in a separate reaction plate. Final substrate concentrations are as follows: **W349X 5-substituted-Trp screen**: 2 mM each of 5-methoxytryptophan, 5-ethoxytryptophan, 2-methyl-5-methoxytryptophan, 5-carboxamidotryptophan, and 5-acetyltryptophan; **W349X single-substrate screen**: 2 mM 5-methoxytryptophan; **active-site site-saturation mutagenesis screens**: 2 mM each of 2-methyltryptophan, 4-bromotryptophan, 5-methoxytryptophan, and 6-chlorotryptophan; 0.2 mM 7-iodotryptophan and 0.2 mM tryptophan. Reactions were incubated at 37 °C for 4 h, quenched via addition of 150 µL 1:1 acetonitrile:1 M HCl, and centrifuged at 4000 x*g* for 10 min. 200 µL of the quenched reaction mixture supernatant was filtered into a 96-well plate for UPLC-MS analysis. Data were collected on an Acquity UHPLC with an Acquity QDA MS detector (Waters) using an Intrada Amino Acid column (Imtakt). Tryptamine product m/z ion counts were used to quantify product formation from the tryptophan reaction mixture from corresponding standard curves (Supplementary Fig. 15).

Cloning of *Pf*TrpB globally random mutagenesis libraries

The plasmid encoding an engineered β-subunit of tryptophan synthase from *Pyrococcus furiosus*, *Pf*TrpB^{2B9} (2B9), was obtained from the Arnold lab.[12] The mutations in this variant were previously shown to confer kinetic and spectroscopic properties that mimic native allosteric activation by the α-subunit of tryptophan synthase.[3] The 2B9-encoding plasmid was used as a template for global random mutagenesis to prepare error-prone libraries. PCR amplification of the 2B9 gene was conducted with the addition

of $MnCl_2$ (100 or 150 µM) to induce mutagenesis. The amplified products were purified by gel electrophoresis and extracted from the gel using a Zymoclean Gel DNA Recovery Kit. The resulting gene libraries were inserted into a pET22(b)+ plasmid via Gibson assembly[10] and transformed into *E. coli* BL21(DE3) cells via electroporation. Cells were recovered in TB media for 30 minutes at 37 °C and 220 RPM. Cells were plated onto LB + 100 µg/mL AMP agar plates and incubated overnight at 37 °C.

Cloning of *Pf*TrpB H275X site-saturation library

To construct a site-saturation mutagenesis library at the H275 site, three primers with the degenerate codons NDT, VHG, and TGG were purchased from IDT and mixed in a 12:9:1 ratio.[4] The 2B9 gene was amplified with this primer mixture, resulting in two gene fragments, which were combined via PCA to generate a full-length gene.[11] The gene was purified by gel electrophoresis and extracted using a Zymoclean Gel DNA Recovery Kit. The resulting gene library was inserted into a pET22(b)+ plasmid via Gibson assembly and transformed into *E. coli* BL21(DE3) cells via electroporation. Cells were recovered in TB media for 30 minutes at 37 °C and 220 RPM. Cells were plated onto LB + 100 µg/mL AMP agar plates and incubated at 37 °C overnight.

Screening of *Pf*TrpB libraries

Individual colonies were picked and used to inoculate wells containing 600 µl TB + 100 µg/mL AMP in a 96-well plate. One column of the plate was used as an internal control with five parent (2B9) colonies, one sterile control, and two non-*Pf*TrpB pET22b negative controls. Plates were grown at 37 °C for 16 h at 200 RPM. Expression cultures in a fresh 96-deep well plate containing TB + 100 µg/mL AMP (630 µL) were inoculated using starter culture (20 µL) and grown at 37 °C and 180 RPM until cultures reached an OD_{600} of at least 0.6 (approximately 3 hours). Expression cultures were chilled on ice for 30 minutes then inoculated with IPTG (1 mM final concentration). Expression plates were incubated at 23 °C for 16 h at 180 RPM then centrifuged at 4000 x*g* for 15 min and the supernatant discarded. Cell pellets were stored at -20 °C until lysed.

Cell pellets were thawed and then resuspended in lysis buffer: 50 mM potassium phosphate buffer (pH = 8.0), 1 mg/mL Hen Egg White Lysozyme (GoldBio), 0.2 mg/mL DNaseI (GoldBio), 1 mM $MgCl_2$, and 300 µM pyridoxal 5′-phosphate (PLP). A volume of 300 µL lysis buffer per well was used. Cells were lysed for 1 h at 37 °C then heat treated at 75 °C for 15 min. After cooling on ice, lysate was spun down at 4000 x*g* at 4 °C for 20 min.

A 96-well plate was loaded with 20 µL substrate mixture (final concentration of 5 mM each 2-methylindole, 4-cyanoindole, 5-methoxyindole, 6-hydroxyindole, and indoline, plus 2.5 mM 7-chloroindole). All indole stocks were prepared in DMSO. For globally random mutagenesis library plate A, potassium phosphate buffer (50 mM, pH = 8.0, 160 µL) containing L-serine (5 mM final concentration) was added, followed by heat-treated

lysate (20 µL). For subsequent plates, lysate volume was increased to 50 µL and buffer volume reduced to 130 µL. Reactions were set up such that the DMSO cosolvent comprised 10% of the final reaction volume (200 µL). Reactions were run at room temperature (25 °C) for 2.5 h and were quenched with 200 µL acetonitrile containing 0.1 M HCl and 1 mM tryptamine (as internal standard). Plates were spun down at 4000 x*g* at 4°C for 20 min. A 200 µL aliquot of each quenched reaction was filtered into a 96-well plate for analysis by UPLC-MS. Product formation was quantified by integration of peaks on single ion retention (SIR) channels corresponding to each expected product, normalized against the tryptamine internal standard.

Expression of *Rgn*TDC and *Pf*TrpB variants

Cells from wells containing putatively activated *Rgn*TDC or *Pf*TrpB variants were inoculated into 5 mL TB + AMP and grown for 16 h at 37 °C. Each variant strain culture was used to inoculate 500 mL TB + AMP, which was subsequently grown at 37 °C for 3 h, or until OD_{600} ~ 1.5. Cultures were chilled on ice for 30 min, and then induced with 1 mM IPTG. Cells expressing *Rgn*TDC were additionally supplemented with 0.5 mM indole, as this was found to boost cell growth. Cultures expressed for 16 h at 23 °C. The next morning, cells were pelleted via centrifugation at 4000 x*g* for 15 min. Supernatants were bleached and discarded and pellets were weighed and stored at -20 °C until lysis.

To purify enzyme variants, cell pellets were thawed at room temperature and then resuspended in lysis buffer: 50 mM potassium phosphate buffer (pH = 8.0), 1 mg/mL Hen Egg White Lysozyme (GoldBio), 0.2 mg/mL DNaseI (GoldBio), 1 mM $MgCl_2$, and 300 µM pyridoxal 5′-phosphate (PLP). A volume of 4 mL of lysis buffer per gram of wet cell pellet was used. After 30 min of shaking at 37 °C, *Rgn*TDC lysis suspensions were disrupted using sonication (5 min; 0.8 s on, 0.2 s off at a power setting of 5). *Pf*TrpB lysis suspensions were heat treated at 75 °C for 15 min as an alternative to sonication. The resulting lysate was spun down at 75,000 x*g* to pellet cell debris. Ni/NTA beads (GoldBio) were added to the supernatant and incubated on ice for 30 to 60 min prior to purification by Ni-affinity chromatography. The column was washed with 3 column volumes of wash buffer A (20 mM imidazole, 50 mM potassium phosphate buffer (pH = 8.0)) and with 2 column volumes of wash buffer B (40 mM imidazole, 50 mM potassium phosphate buffer (pH = 8.0); *Pf*TrpB variants only), and the proteins were eluted with an elution buffer (250 mM imidazole, 50 mM potassium phosphate buffer (pH = 8.0)). Elution of the desired protein product was monitored by the disappearance of its bright yellow color (resulting from the PLP cofactor) from the column. The protein product was dialyzed to < 1 µM imidazole, dripped into liquid nitrogen to flash freeze, and stored at -80 °C. The concentration of protein was determined by Bradford assay upon thawing. Generally, this procedure yielded 50 – 200 mg per L culture for *Rgn*TDC variants and 200 – 500 mg per L culture for *Pf*TrpB variants.

Turnover analysis of *Rgn*TDC variants

*Rgn*TDC variants were thawed on ice from storage at -80 °C and then centrifuged at 15,000 x*g* for 5 min to pellet aggregated protein. The supernatant was then diluted between 1:1 and 1:100 in 50 mM potassium phosphate buffer (pH = 8.0) (depending on enzyme concentration). Reactions were run in plastic 96-well plates with 10 mM Trp analog, either 33 nM or 1 µM TDC variant, and 1000 equivalents PLP relative to TDC with a total volume of 100 µL in 50 mM potassium phosphate buffer (pH = 8.0). Reactions were incubated for 16 h at 37 °C. Reactions were quenched with 200 µL acetonitrile and further diluted with 100 µL H_2O. Quenched reactions were centrifuged at 15,000 x*g* for 10 min prior to analysis by UPLC-MS. Enzymatic activity was quantified by integrating the substrate and product UV absorbance peaks at 280 nm. Reactions were run in triplicate, with replicate reactions run on different days with freshly thawed enzyme (Fig 2g).

[PLP] vs. activity for *Rgn*TDC variants

*Rgn*TDC variants were thawed and prepared as described above. 10 mM 6-chlorotryptophan was added to a solution with varying molar equivalents of PLP relative to TDC (500 nM, 5 µM, 50 µM, or 500 µM). TDC was added such that the final concentration was 50 nM and the solution was diluted up to 100 µl with 50 mM potassium phosphate buffer (pH = 8.0). Reactions were incubated for 16 h at 37 °C. Reactions were quenched with 200 µL acetonitrile and diluted with 100 µL H_2O. Quenched reactions were centrifuged at 15,000 x*g* for 10 min prior to UPLC-MS injection of the supernatant. Enzymatic activity was quantified by integrating the substrate and product UV absorbance peaks at 280 nm. Reactions were run in duplicate, with replicate reactions run on separate days with freshly thawed enzyme (Supplementary Fig. 22).

*Rgn*TDC substrate multiplexed timecourse reaction

*Rgn*TDC variants were thawed and prepared as described above. Reaction conditions included 0.25 µM *Rgn*TDC, 2.5 µM PLP, and one of two sets of substrate mixes: 2.5 mM tryptophan, 2-methyltryptophan, 4-bromotryptophan, and 6-chlorotryptophan; or 0.25 mM tryptophan and 6-chlorotryptophan, 2.5 mM 2-methyltryptophan and 4-bromo-tryptophan. Reactions were filled to 100 µl with 50 mM potassium phosphate pH = 8.0. Reactions were conducted in triplicate and incubated for 4 h at 37 °C. Reactions timepoints were taken by quenching 10 µl of the reaction solution in 190 µl of acetonitrile, followed by dilution with 200 µl H_2O. Quenched reactions were centrifuged at 15,000 x*g* for 10 min prior to UPLC-MS injection of the supernatant. Product abundance was quantified via single-ion retention and fit to product standard curves (Supplementary Fig. 16).

Michaelis-Menten analysis of *Rgn*TDC variants

*Rgn*TDC variants were thawed on ice from storage at -80 °C and then centrifuged at 15,000 x*g* for 5 min to pellet aggregated protein. The supernatant was then diluted between 1:1 and 1:100 in 50 mM potassium phosphate buffer (pH = 8.0), depending on enzyme concentration. Reaction conditions used 0.1 – 20 mM tryptophan analog with 10 equivalents

of PLP cofactor relative to TDC in 1.7 mL Eppendorf tubes with a total volume of 100 μL in 50 mM potassium phosphate buffer (pH = 8.0). The enzyme and substrate solutions were equilibrated to 37 °C prior to mixing. Final enzyme concentrations were 0.1 – 5 μM. At various time points after addition of *Rgn*TDC, 20 μL aliquots of the reaction solution were quenched in 180 μL acetonitrile and diluted with 200 μL H_2O. Quenched reactions were centrifuged at 15,000 x*g* for 10 min prior to UPLC-MS injection of the supernatant. Enzymatic activity was quantified by integrating the substrate and product UV absorbance peaks at 280 nm. All replicate reactions were run on different days with fresh enzyme. Initial velocity slopes were fit using the revised Michaelis-Menten equation as described by Johnson 2019.[13]

Single substrate activity analysis of *Pf*TrpB variants

*Pf*TrpB variants were thawed on ice from storage at -80 °C and then centrifuged at 15,000 x*g* for 5 min to pellet aggregated protein. Each variant was diluted in 50 mM potassium phosphate buffer (pH = 8.0) to a concentration of 40 μM. Single substrate reactions were prepared in duplicate for each *Pf*TrpB variant on a 96-well plate. Reaction conditions included 10 mM indole analog, 30 mM Ser, 2 μM *Pf*TrpB (5000 max TON), and 10% DMSO in 100 mM potassium phosphate buffer (pH = 8.0). Reactions were run at 37 °C for 1 h. Reaction aliquots (75 μL) were quenched with 75 μL acetonitrile + 0.1 M HCl + 1 mM tryptamine (as internal standard) and diluted with an additional 150 uL 1:1 acetonitrile:H_2O. Quenched reactions were centrifuged at 15,000 x*g* for 10 min then filtered into a 96-well plate for UPLC-MS analysis. Product formation was quantified by integration of peaks on single ion retention (SIR) channels corresponding to each expected product, normalized against the tryptamine internal standard.

Michaelis-Menten analysis of *Pf*TrpB variants

*Pf*TrpB variants were thawed on ice from storage at -80 °C and then centrifuged at 15,000 x*g* for 5 min to pellet aggregated protein. The supernatant was diluted in 100 – 200 mM potassium phosphate buffer (pH = 8.0) to obtain sufficiently dilute concentrations for initial velocity kinetic measurements.

Indole: Rate of formation of Trp from indole and Ser was measured at 290 nm, as described previously.[14] *Pf*TrpB variants (0.34 – 1.57 μM final concentration) in 200 mM potassium phosphate buffer (pH = 8.0) were pre-incubated in quartz cuvettes at 37 °C. Indole (3 – 400 μM final concentration) was added, followed by Ser (2.5 mM for 2B9 and H275E, 25 mM for H275R). Indole stocks were prepared using DMSO, such that the final reaction volume (400 μL) was comprised of 5% DMSO. Cuvettes were inverted to mix, and absorbance at 290 nm was collected for 2 min. All replicate reactions were run on two different days with fresh enzyme. Initial velocity slopes were calculated and fit using the revised Michaelis-Menten equation as described by Johnson 2019.[13]

Indoline: Formation of 2,3-dihydroisotryptophan (DIT) from indoline and Ser was measured by integration of UPLC peaks at 254 nm. A calibration curve was used to convert integration of the UPLC peak to a concentration of DIT (Supplementary Fig. 37). Reaction

conditions included 1 – 50 mM indoline, 10 mM Ser, 1 – 2 µM *Pf*TrpB, and 5% DMSO in 100 mM potassium phosphate buffer (pH = 8.0) in 1.7 mL epi-tubes. The enzyme and substrate solutions were equilibrated to 37 °C prior to mixing. Reaction aliquots (50 µL) were quenched in acetonitrile (125 µL) at various time points (5 – 60 min) and diluted with 75 µL water (1:5 dilution factor). Quenched samples were centrifuged (15,000 x*g*, 5 min) and analyzed by UPLC-MS. All replicate reactions were run on three different days with fresh enzyme. Initial velocity slopes were calculated and fit using the revised Michaelis-Menten equation as described by Johnson 2019.[13]

UV-Vis spectroscopy of *Pf*TrpB variants

*Pf*TrpB variants were thawed on ice from storage at -80 °C and then centrifuged at 15,000 x*g* for 5 min to pellet aggregated protein. The supernatant was diluted in 200 mM potassium phosphate buffer (pH = 8.0) to obtain a final concentration of 300 µM. A baseline spectrum (600-250 nm, 1 nm interval, fast scan) was collected on 200 mM potassium phosphate buffer (284 µL, pH = 8.0) at 25 °C. *Pf*TrpB (20 µL) was added to the cuvette for a final concentration of 20 µM enzyme and an enzyme-only spectrum was collected. To study Ser binding and rate of the shunt pathway, Ser (12.5 µL, 20 mM final concentration) was added, and the cuvette was inverted to mix. Spectra were collected every 10 minutes for 12 scans (110 minutes total) to track pyruvate formation (320 nm) over time. To study Trp binding, Trp was titrated into a sample of 20 µM *Pf*TrpB and a spectrum was collected at each concentration (0-1200 µM).

Protein crystallography of *Pf*TrpB-H275E

Crystals of the H275E *Pf*TrpB^{2B9} variant were grown in sitting drops against a 1-mL reservoir containing 13-21% PEG3350 and 0.1 M Na HEPES buffer (pH = 7.85), with mother liquor containing 2 µL of H275E (8.0 or 15 mg/mL) and 2 µL of well solution. Crystals grew over the course of several days and were stable in the dark over several weeks. Trp- and 4-Cl-Trp-bound crystals were prepared by addition of solid Trp or 4-Cl-Trp (approx. 1 mg) to pre-formed crystals. Crystals were cryoprotected by dredging through Fomblin-Y then through Paratone-N and flash frozen in liquid N_2. Data were collected remotely at the Argonne National Laboratory Advanced Photon Source on either beamline 21ID-D (PDB ID: 7RNQ) or beamline 23ID-B (PDB ID: 7RNP, 7ROF). Data were integrated and scaled using XDS and AIMLESS.[15,16] Structures were solved by molecular replacement with PHASER, using CCP4.[17,18] Search models for each structure were comprised of a single monomer of a previously solved *Pf*TrpB^{2B9} structure, where the H275 site was deleted, ligands and waters were removed, and the remaining model was subjected to geometric refinement in Refmac5. PDB ID: 6AM7 was used as the search model for the unbound H275E structure, and PDB ID: 6AM8 was used as the search model for the Trp- and 4-Cl-Trp-bound structures. Model building was performed in Coot and refinement was conducted using Refmac5. The TLS motion server was used to calculate TLS operators; we selected 10 operators for each chain in the asymmetric unit.[19] The MolProbity server was used to evaluate the structure prior to final refinement.[19,20] Crystallographic and refinement statistics are reported in Supplementary Table 3.

Tryptophan analog biosynthesis and purification

For each tryptophan analog, the corresponding indole derivative (~1 mmol) was added to a 100 mL pressure flask and dissolved in MeOH (5 mL). L-Serine (3 mmol) was added, and the resulting solution was diluted to just under 100 mL with 50 mM potassium phosphate buffer (pH = 8.0). PLP was added such that the final concentration was 300 μM. Then, 2B9 was added at 0.1% mol catalyst relative to the indole analog. The solution was incubated at 75 °C for 16 h. Following UPLC-MS analysis of conversion, the solution was heat-treated at 90 °C for 30 min. Solutions were evaporated down to 10 mL, filtered, and run over C18 via flash chromatography with H_2O/MeOH. Tryptophan products typically eluted between 10-35% MeOH. Product-containing fractions were combined and evaporated down to ~2-3 mL, where the solutions were transferred to pre-tared flasks, evaporated to dryness, resuspended in H_2O, flash-frozen, and lyophilized for 24 – 48 h. Resulting powders were weighed and submitted for ^{1}H-NMR analysis, with final reported yields taking hydration states into account (*all tryptophan analog products were isolated as hydrates*).

2-methyltryptophan

2-methyltryptophan was isolated as an off-white powder (81.8 mg) in 94% yield. ^{1}H NMR (500 MHz, MeOD) δ 7.64 – 7.58 (m, 1H), 7.31 – 7.25 (m, 1H), 7.06 (td, *J* = 7.1, 1.4 Hz, 1H), 7.02 (td, *J* = 7.5, 1.3 Hz, 1H), 3.86 (dd, *J* = 9.9, 4.2 Hz, 1H), 3.52 – 3.46 (m, 1H), 3.08 (dd, *J* = 15.2, 10.0 Hz, 1H), 2.44 (s, 3H). Spectrum matched previously reported spectra.[14]

2-methyl-5-methoxytryptophan

2-methyl-5-methoxytryptophan was isolated as a tan powder (164 mg) in 66% yield. ^{1}H NMR (500 MHz, MeOD) δ 7.18 (d, *J* = 2.4 Hz, 1H), 7.15 (d, *J* = 8.6 Hz, 1H), 6.69 (dd, *J* = 8.7, 2.4 Hz, 1H), 3.85 (s, 3H), 3.76 (dd, *J* = 9.8, 4.2 Hz, 1H), 3.38 (dd, *J* = 15.0, 4.2 Hz, 1H), 2.97 (dd, *J* = 14.9, 9.8 Hz, 1H), 2.41 (s, 3H). ^{13}C NMR (126 MHz, MeOD) δ 174.08, 153.91, 134.36, 131.06, 128.63, 110.68, 110.27, 104.24, 99.64, 55.86, 54.99, 26.93, 10.18.
MS/ESI m/z for $[M+H]^+$; C13H16N2O3; calculated 247.10882, observed 247.1087.

4-bromotryptophan

4-bromotryptophan was isolated as a white powder (66.9 mg) in 59% yield. ^{1}H NMR (500 MHz, MeOD) δ 7.38 (d, 1H), 7.28 (s, 1H), 7.23 (d, *J* = 7.4 Hz, 1H), 7.00 (t, *J* = 7.9 Hz, 1H), 4.07 (dd, *J* = 10.3, 4.6 Hz, 1H), 3.98 (dd, *J* = 15.1, 4.6 Hz, 1H), 3.13 (dd, *J* = 15.1, 10.3 Hz, 1H). Spectrum matched previously reported spectra.[21]

5-acetyltryptophan

5-acetyltryptophan was isolated as a white powder (162 mg) in 42% yield. ^{1}H NMR (500 MHz, MeOD) δ 8.54 (d, *J* = 1.6 Hz, 1H), 7.85 (dd, *J* = 8.6, 1.7 Hz, 1H), 7.44 (d, *J* = 8.6 Hz, 1H), 7.32 (s, 1H), 3.87 (dd, *J* = 8.7, 4.3 Hz, 1H), 3.54 (dd, *J* = 15.1, 4.3 Hz, 1H), 3.24 (dd, *J* = 15.2, 8.7 Hz, 1H), 2.71 (s, 3H). ^{13}C NMR (126 MHz, D_2O) δ 204.10, 174.67, 139.52, 128.34, 126.68, 126.36, 121.99, 121.84, 111.81, 109.80, 55.11, 26.35, 26.09.
MS/ESI m/z for $[M+H]^+$; C13H14N2O3; calculated 245.0932, observed 245.0930.

5-carboxamidotryptophan

5-carboxamidotryptophan was isolated as a white powder (163 mg) in 52% yield. ^{1}H NMR (500 MHz, MeOD) δ 8.32 (dd, 1H), 7.74 (dd, *J* = 8.6, 1.7 Hz, 1H), 7.44 (dd, *J* = 8.6, 0.7 Hz, 1H), 7.31 (s, 1H), 3.88 (dd, *J* = 9.1, 4.0 Hz, 1H), 3.55 (ddd, *J* = 15.1, 4.1, 1.0 Hz, 1H), 3.19 (dd, *J* = 15.3, 9.1 Hz, 1H). Spectrum matched previously reported spectra.[21]

5-ethoxytryptophan

5-ethoxytryptophan was isolated as a white powder (82.7 mg) in 59% yield. ^{1}H NMR (500 MHz, MeOD) δ 7.26 (d, *J* = 5.3 Hz, 1H), 7.25 (s, 1H), 7.17 (s, 1H), 6.79 (dd, *J* = 8.9, 2.2 Hz, 1H), 4.15 – 4.06 (m, 2H), 3.84 (dd, *J* = 9.3, 4.1 Hz, 1H), 3.14 – 3.05 (m, 1H), 1.42 (t, *J* = 6.9 Hz, 4H). ^{13}C NMR (126 MHz, D_2O) δ 151.88, 131.88, 127.20, 125.80, 112.78, 112.57, 108.23, 102.41, 65.51, 55.32, 27.44, 14.11.
MS/ESI m/z for $[M+H]^+$; C13H16N2O3; calculated 247.10882, observed 247.1087.

5-methoxytryptophan

5-methoxytryptophan was isolated as a brown powder (556 mg) in 48% yield. ^{1}H NMR (500 MHz, MeOD) δ 7.27 (d, *J* = 3.3 Hz, 1H), 7.25 (d, *J* = 2.8 Hz, 1H), 7.17 (s, 1H), 6.79 (dd, *J* = 8.8, 2.4 Hz, 1H), 3.87 (s, 3H), 3.85 (ddd, *J* = 9.5, 3.9, 1.9 Hz, 1H), 3.52 – 3.46 (m, 1H), 3.11 (dd, *J* = 15.2, 9.6 Hz, 1H). Spectrum matched previously reported spectra.[22]

6-chlorotryptophan

6-chlorotryptophan was isolated as a white powder (63.8 mg) in 67% yield. ^{1}H NMR (500 MHz, MeOD) δ 7.68 (d, *J* = 8.5 Hz, 1H), 7.38 (d, *J* = 1.9 Hz, 1H), 7.23 (s, 1H), 7.04 (dd, 1H), 3.83 (ddd, *J* = 9.4, 5.6, 4.2 Hz, 1H), 3.47 (dd, *J* = 15.2, 4.2 Hz, 1H), 3.15 (dd, 1H). Spectrum matched previously reported spectra.[21]

6-nitrotryptophan

6-nitrotryptophan was isolated as a yellow powder (245 mg) in 98% yield. ^{1}H NMR (500 MHz, MeOD) δ 8.36 (d, *J* = 2.0 Hz, 1H), 7.98 (dd, *J* = 8.8, 2.1 Hz, 1H), 7.86 (d, *J* = 8.8 Hz, 1H), 7.57 (s, 1H), 3.79 (dd, *J* = 8.2, 4.6 Hz, 1H), 3.47 – 3.41 (m, 1H), 3.26 – 3.19 (m, 1H). Spectrum matched previously reported spectra.[21]

7-iodotryptophan

7-iodotryptophan was isolated as a white powder (94.3 mg) in 79% yield. ^{1}H NMR (500 MHz, MeOD) δ 7.75 (dd, *J* = 7.9, 0.9 Hz, 1H), 7.54 (d, *J* = 7.4 Hz, 1H), 7.30 (s, 1H), 6.88 (t, *J* = 7.7 Hz, 1H), 3.86 (dd, *J* = 9.1, 4.2 Hz, 1H), 3.50 (dd, 1H), 3.16 (dd, 1H). Spectrum matched previously reported spectra.[21]

Cascade synthesis and isolation of tryptamines

4-6 mmol (1.4 mmol for 6-chloroindole) of the corresponding indole analog was added to a 1 L Erlenmeyer flask and dissolved in 20 mL MeOH. 12 mmol serine was added, and the resulting solution was diluted up to just under 500 mL with 50 mM potassium phosphate buffer pH = 8.0. PLP was added such that the final concentration was 300 µM. Then, H275E was added at 0.05% mol catalyst relative to the indole analog (0.2 mg/mL). The solution was incubated at 75 °C for 16 h. (H275E was found to be activating at 75 °C, Supplementary Fig. 33). Following UPLC-MS analysis of conversion, the solution was cooled to 37 °C, upon which *Rgn*TDC was added at 0.02 – 0.2% mol catalyst relative to the indole (0.09 – 0.9 mg/mL). The solutions were incubated at 37 °C for 24 h. Solutions were then evaporated down to 50 – 100 mL. To break emulsions, the solutions were acidified with 6 M HCl until pH < 1, 100 mL ethyl acetate (EtOAc) was added, and the resulting mixtures were centrifuged at 4000 x g for 10 min. These solutions were added to a separatory funnel, the aqueous layer was drained, and the organic layer removed. This was repeated twice more, with 2 mL 6 M HCl added in between extractions. Then, the aqueous layer was alkalized with 6 M NaOH until pH > 12.* Tryptamine products were then extracted 3x with 150 mL EtOAc, with 2 mL 6 M NaOH added in between extractions to the aqueous layer. Organic layers were pooled, dried with sodium sulfate, filtered, and evaporated down to 5-10 mL. Solutions were transferred to 20 mL scintillation vials, evaporated to near dryness (tryptamines were observed as liquids at 50 °C), and dried under vacuum overnight. Dried samples were weighed and submitted for ^{1}H and ^{13}C NMR analysis.

2-methyltryptamine

F98V *Rgn*TDC was used as 0.2% mol catalyst for the reaction with 2-methylindole. 2-methyltryptamine was isolated as a reddish oil (166 mg) in 19% yield. ^{1}H NMR (500 MHz, DMSO) δ 7.40 (dd, *J* = 7.6, 1.1 Hz, 1H), 7.22 (dt, *J* = 8.0, 1.0 Hz, 1H), 7.00 – 6.86 (m, 2H), 2.75 – 2.67 (m, 4H), 2.33 (s, 3H), 2.00 (s, 1H). Spectrum matched previously reported spectra.[2]

4-bromotryptamine

L355M *Rgn*TDC was used as 0.02% mol catalyst for the reaction with 4-bromoindole. 4-bromotryptamine was isolated as a light pink solid (995 mg) in 79% yield. ^{1}H NMR (500 MHz, DMSO) δ 11.16 (s, 1H), 7.36 (dd, *J* = 8.1, 0.9 Hz, 1H), 7.23 (s, 1H), 7.15 (dd, *J* = 7.5, 0.9 Hz, 1H), 6.95 (t, *J* = 7.8 Hz, 1H), 2.97 (dd, 2H), 2.84 (dd, *J* = 7.9, 6.5 Hz, 2H), 1.42 (s, 2H). Spectrum matched previously reported spectra.[2]

5-ethoxytryptamine

W349K *Rgn*TDC was used as 0.02% mol catalyst for the reaction with 5-ethoxyindole. 5-ethoxytryptamine was isolated as a tan solid (353 mg) in 27% yield. ^{1}H NMR (500 MHz, DMSO) δ 10.59 (s, 1H), 7.21 (d, *J* = 8.7 Hz, 1H), 7.07 (d, *J* = 2.3 Hz, 1H), 6.98 (d, *J* = 2.4 Hz, 1H), 6.70 (dd, *J* = 8.7, 2.4 Hz, 1H), 4.01 (q, *J* = 6.9 Hz, 2H), 2.80 (t, *J* = 6.9 Hz, 2H), 2.71 (t, *J* = 7.1 Hz, 2H), 1.42 (s, 2H), 1.34 (t, *J* = 7.0 Hz, 3H). ^{13}C NMR (126 MHz, DMSO) δ 152.49, 131.92, 128.15, 123.71, 112.81, 112.34, 111.86, 101.75, 63.84, 43.16, 30.08, 15.42.

MS/ESI m/z for $[M+H]^+$; C12H16N2O; calculated 205.1335, observed 205.1333.

5-methoxytryptamine

W349K *Rgn*TDC was used as 0.02% mol catalyst for the reaction with 5-methoxyindole. 5-methoxytryptamine was isolated as a dark brown solid (387 mg) in 30% yield, which co-purified with 70 mg 5-methoxytryptophan. ^{1}H NMR (500 MHz, DMSO) δ 10.60 (s, 1H), 7.22 (d, *J* = 8.7 Hz, 1H), 7.08 (d, *J* = 2.4 Hz, 1H), 6.99 (d, *J* = 2.4 Hz, 1H), 6.71 (dd, *J* = 8.7, 2.5 Hz, 1H), 3.76 (s, 3H), 2.81 (t, *J* = 7.2 Hz, 2H), 2.72 (t, *J* = 7.1 Hz, 2H), 1.68 (s, 2H). Spectrum matched previously reported spectra.[2]

6-chlorotryptamine

F98V *Rgn*TDC was used as 0.02% mol catalyst for the reaction with 6-chloroindole. 6-chlorotryptamine was isolated as an off-white solid (144 mg) in 50% yield. ^{1}H NMR (500 MHz, DMSO) δ 10.97 (s, 1H), 7.52 (d, *J* = 8.4 Hz, 1H), 7.37 (d, *J* = 1.9 Hz, 1H), 7.19 (d, *J* = 2.2 Hz, 1H), 6.98 (dd, *J* = 8.4, 1.9 Hz, 1H), 2.84 (t, *J* = 14.5 Hz, 2H), 2.77 (t, *J* = 7.1 Hz, 2H), 1.85 (s, 3H). Spectrum matched previously reported spectra.[2]

6-nitrotryptamine

F98V *Rgn*TDC was used as 0.02% mol catalyst for the reaction with 6-nitroindole. Purification was carried out as described above until the solution was basified. The bright yellow solution was turned bright red upon addition of NaOH, and a yellow precipitate was observed. The precipitate was found to be insoluble in both diethyl ether and EtOAc and was filtered from both the aqueous and organic layers. Upon washing with cold water and diethyl ether, a yellow solid was obtained. The remaining aqueous solution was extracted 3x with diethyl ether, resulting in a pale-yellow organic layer. The organic extraction was evaporated dry, and the resulting yellow solid was combined with the filtered solid via resuspension in water. The resuspension was flash frozen and lyophilization was carried out to remove water. 6-nitrotryptamine was isolated as an initially brown powder (which after 2 weeks turned yellow) (495.5 mg) in 47% yield. Interestingly, this product produces a deep red color when dissolved in solution, which seems to be indicative of the amine group being deprotonated. ^{1}H NMR (500 MHz, ACN) δ 8.32 (d, *J* = 2.2 Hz, 1H), 7.81 (dd, *J* = 8.8, 2.1 Hz, 1H), 7.57 (d, *J* = 8.8 Hz, 1H), 7.52 (s, 1H), 2.90 – 2.82 (m, 4H). Spectrum matched previously reported spectra.[2]

NMR spectra

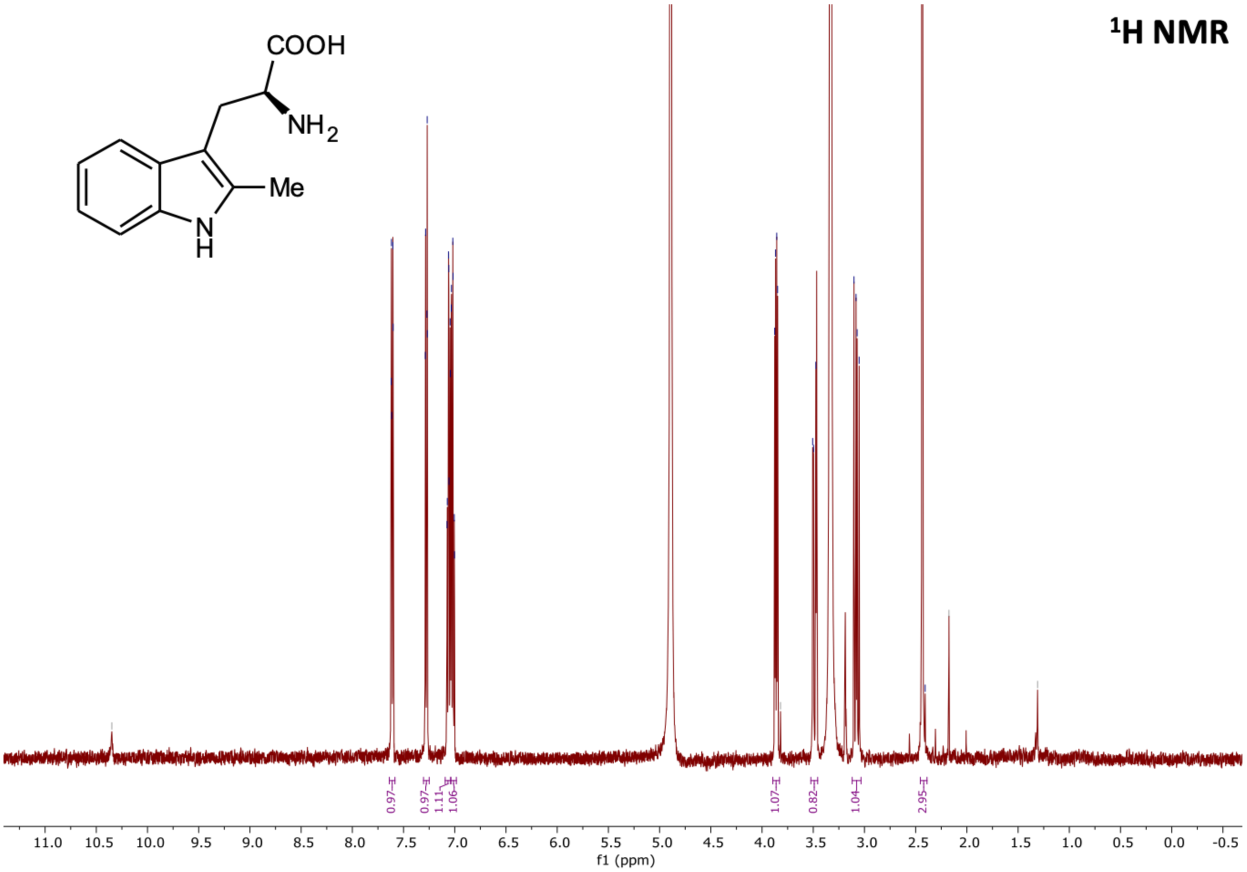

Supplementary Figure 43. ^{1}H NMR spectrum of (S)-2-amino-3-(2-methyl-1H-indol-3-yl)propanoic acid.

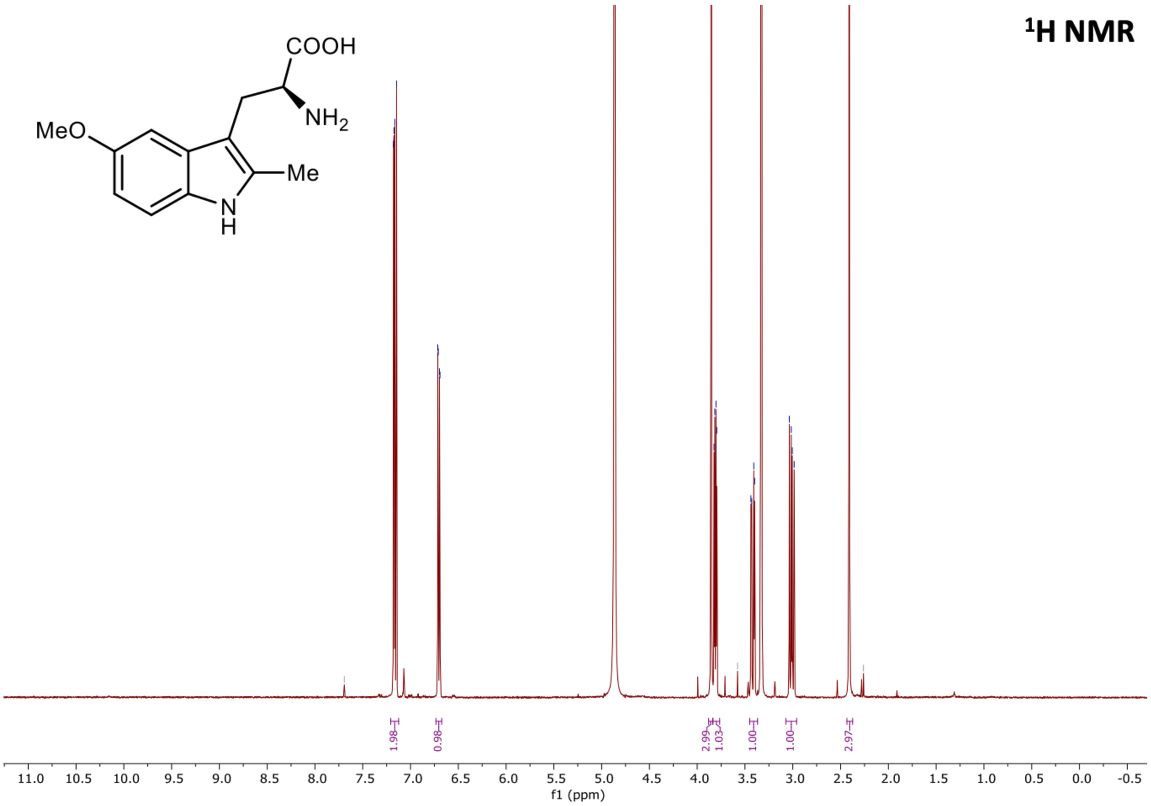

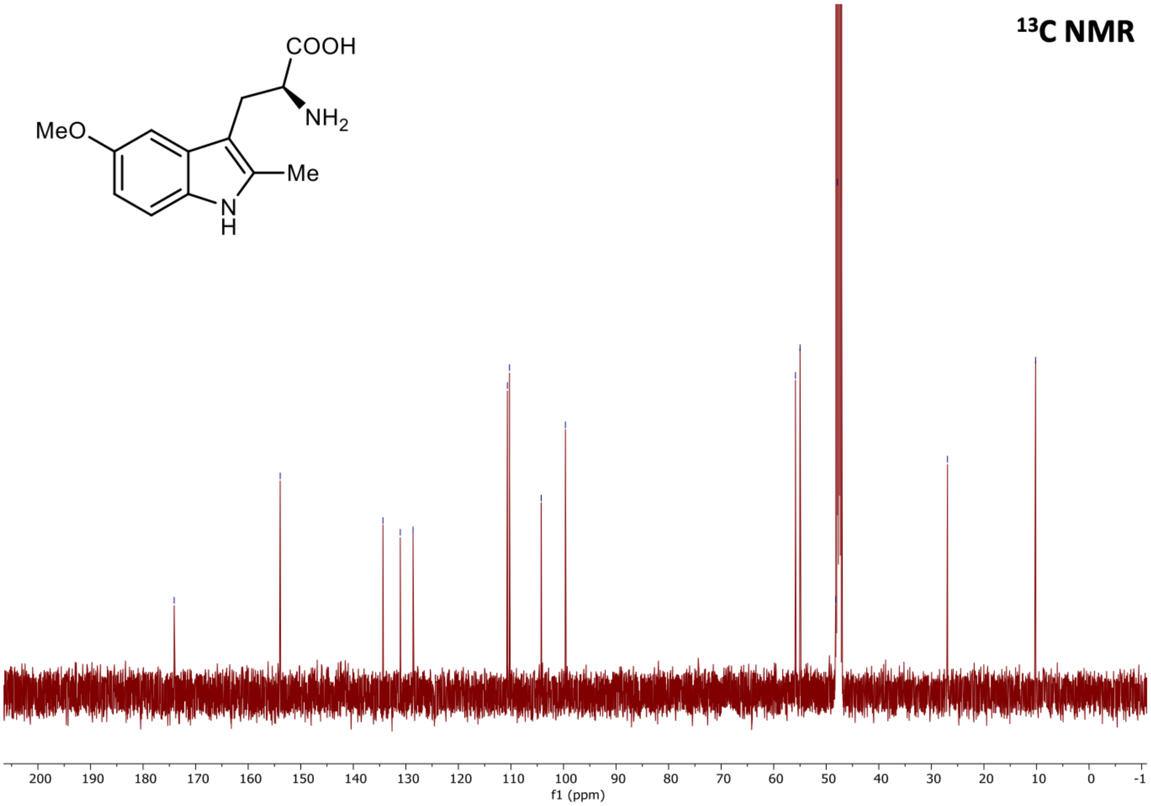

Supplementary Figure 44. ^{1}H and ^{13}C NMR spectra of (*S*)-2-amino-3-(5-methoxy-2-methyl-1H-indol-3-yl)propanoic acid.

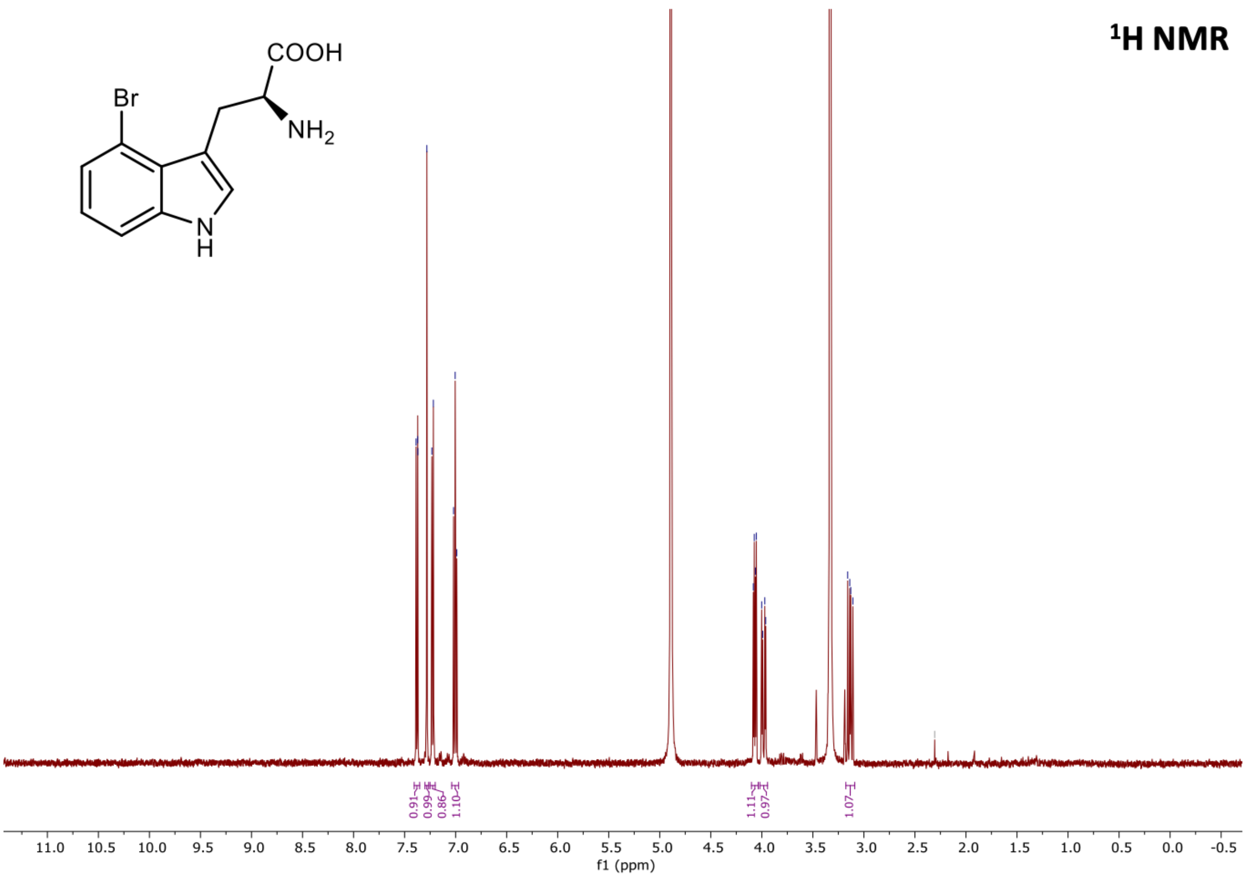

Supplementary Figure 45. ^{1}H NMR spectrum of (S)-2-amino-3-(4-bromo-1H-indol-3-yl)propanoic acid.

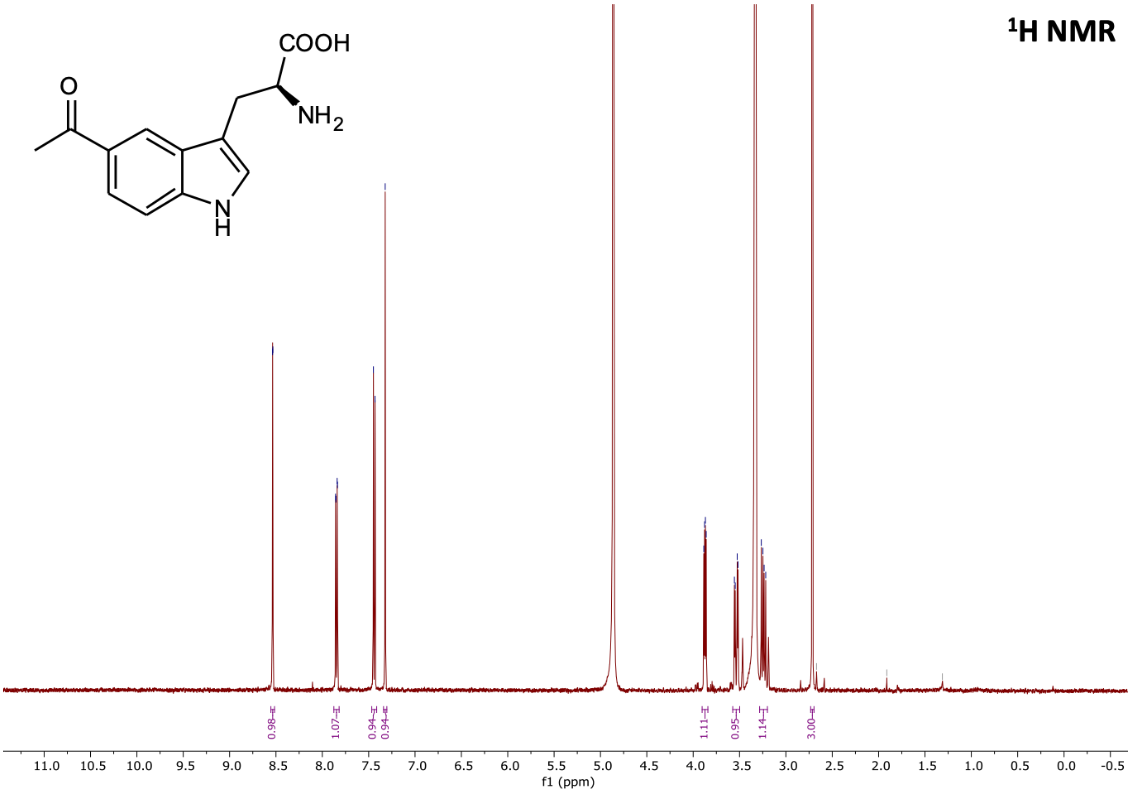

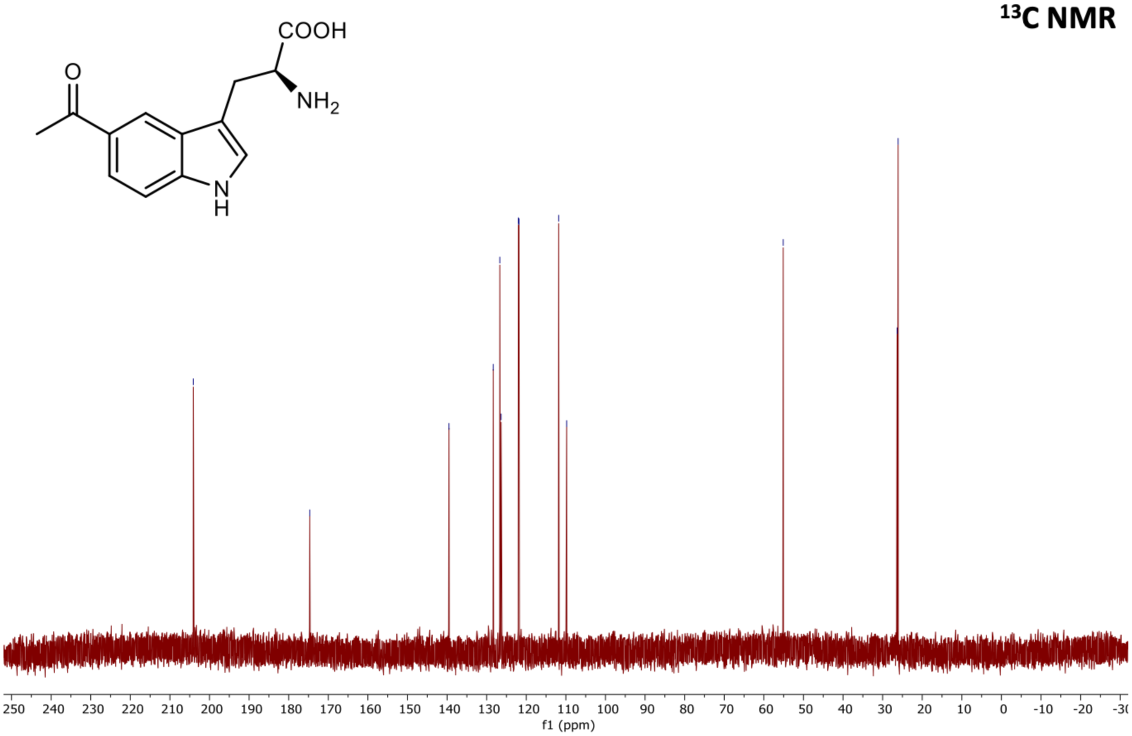

Supplementary Figure 46. ^{1}H and ^{13}C NMR spectra of (S)-3-(5-acetyl-1H-indol-3-yl)-2-aminopropanoic acid.

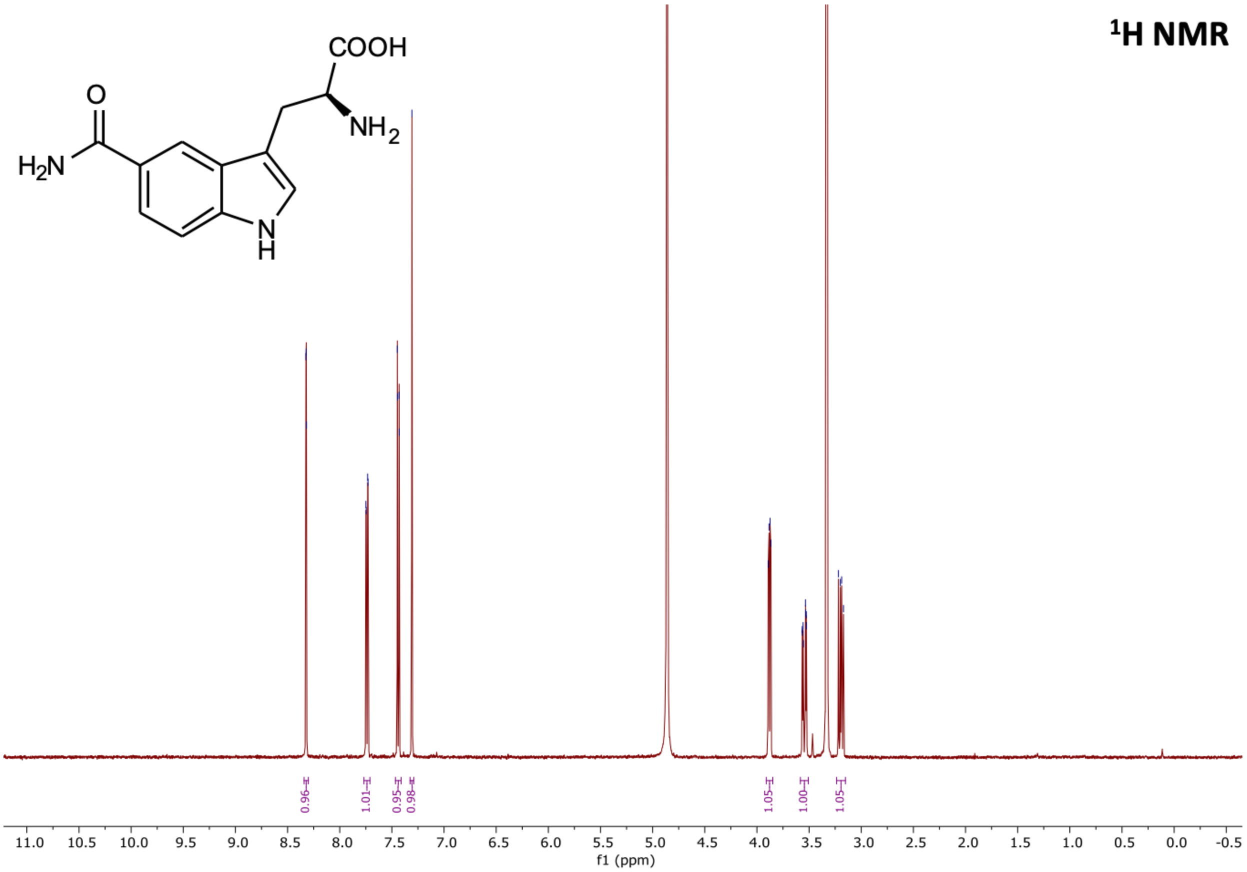

Supplementary Figure 47. ^{1}H NMR spectrum of (S)-2-amino-3-(5-carbamoyl-1H-indol-3-yl)propanoic acid.

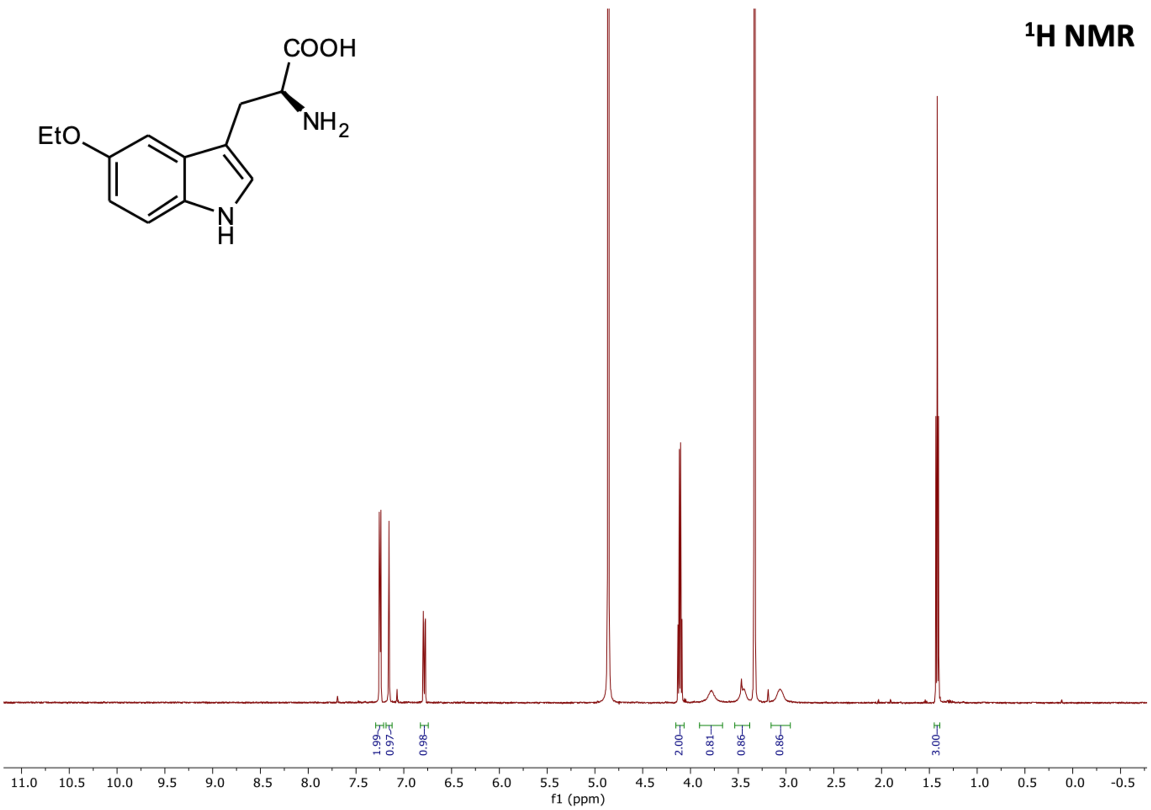

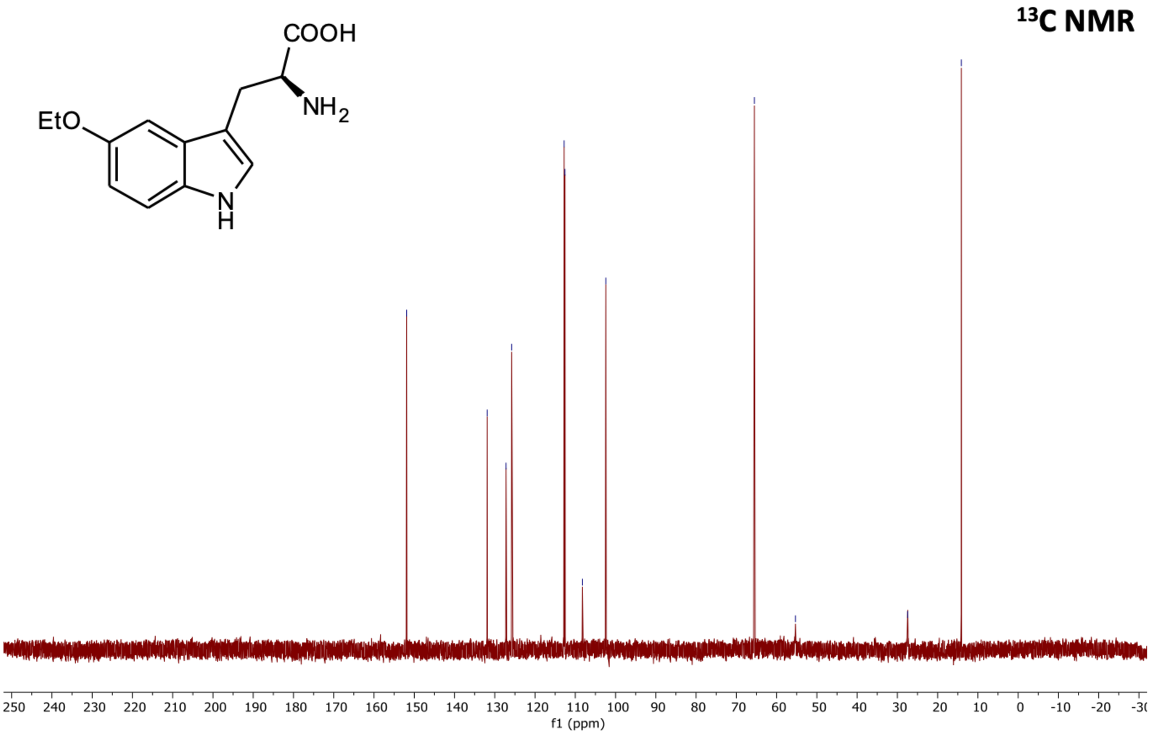

Supplementary Figure 48. ^{1}H and ^{13}C NMR spectra of (S)-2-amino-3-(5-ethoxy-1H-indol-3-yl)propanoic acid.

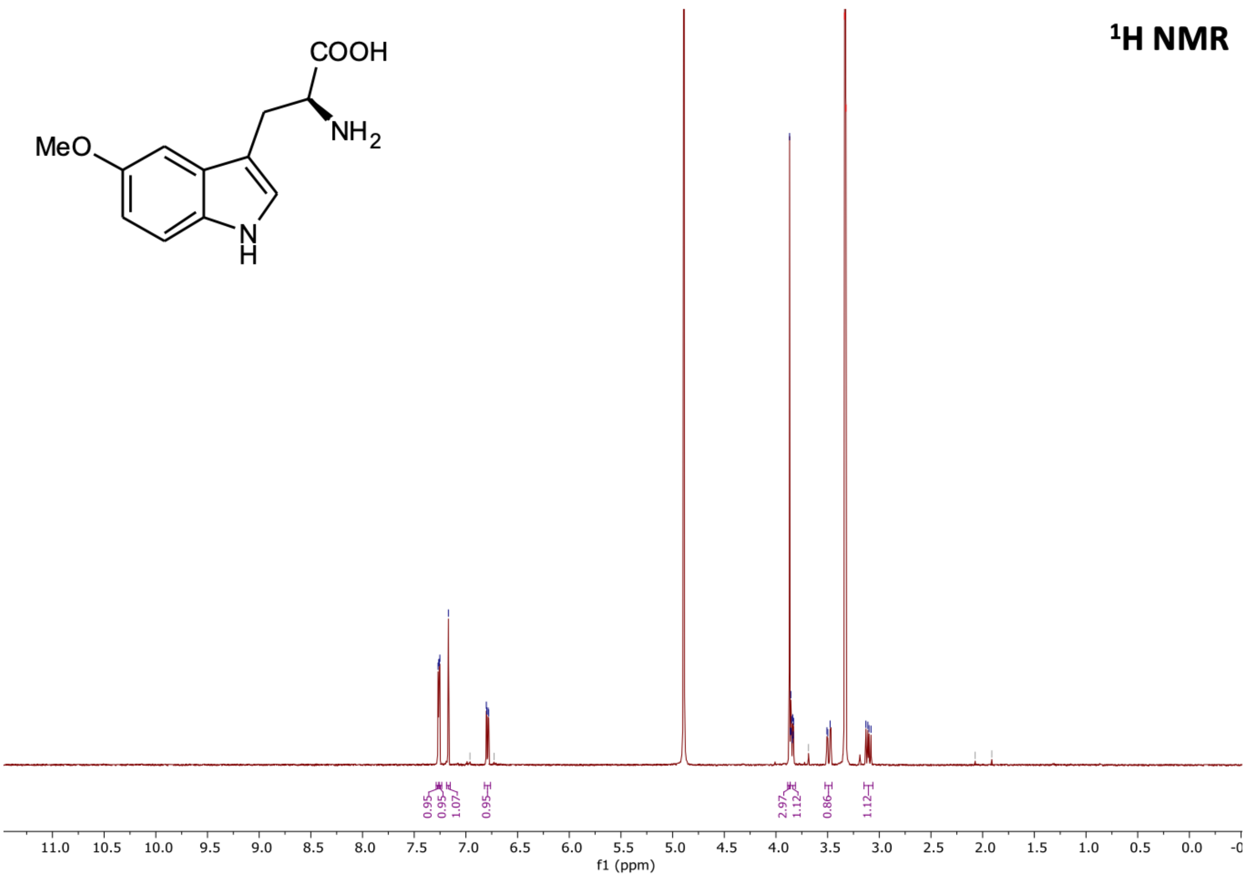

Supplementary Figure 49. ^{1}H NMR spectrum of (S)-2-amino-3-(5-methoxy-1H-indol-3-yl)propanoic acid.

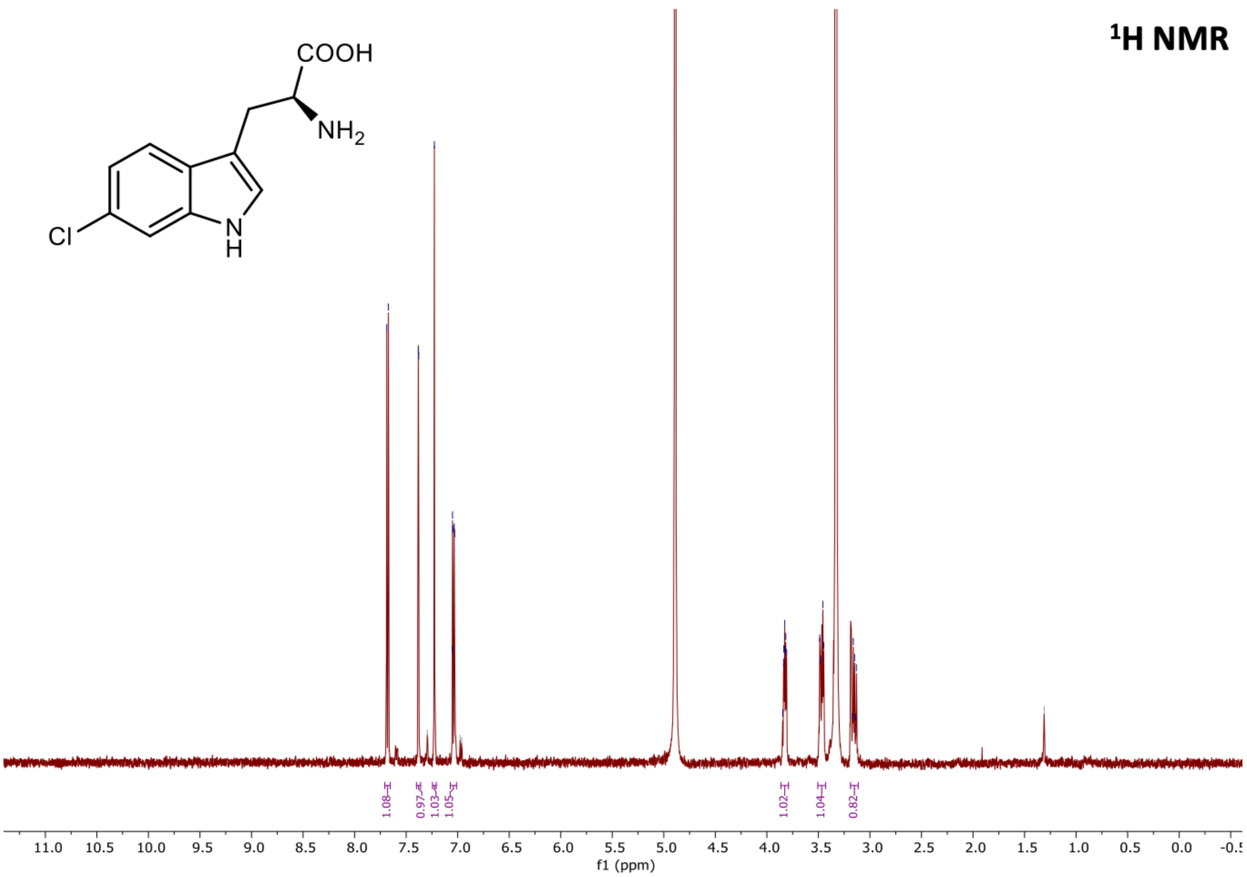

Supplementary Figure 50. ^{1}H NMR spectrum of (S)-2-amino-3-(6-chloro-1H-indol-3-yl)propanoic acid.

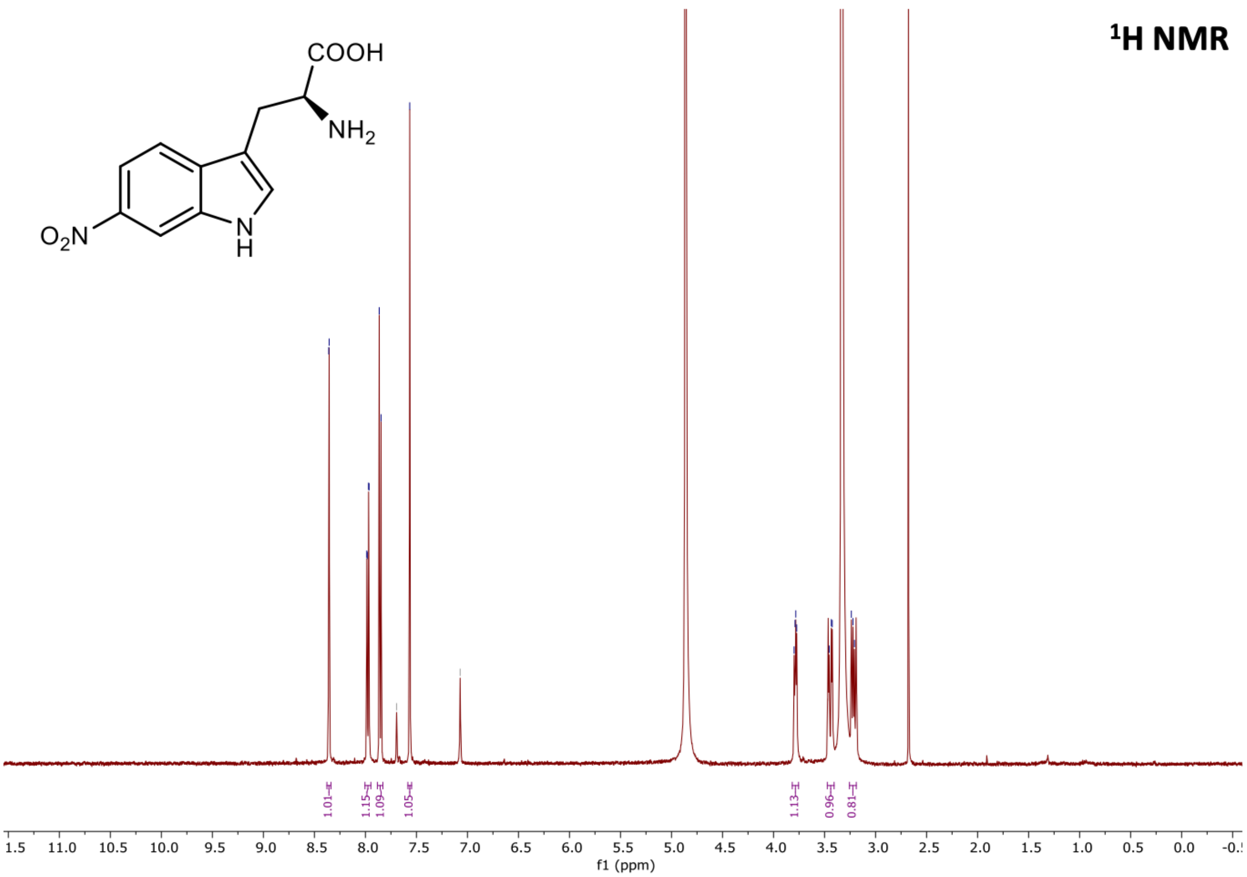

Supplementary Figure 51. ^{1}**H NMR spectrum of (S)-2-amino-3-(6-nitro-1H-indol-3-yl)propanoic acid.**

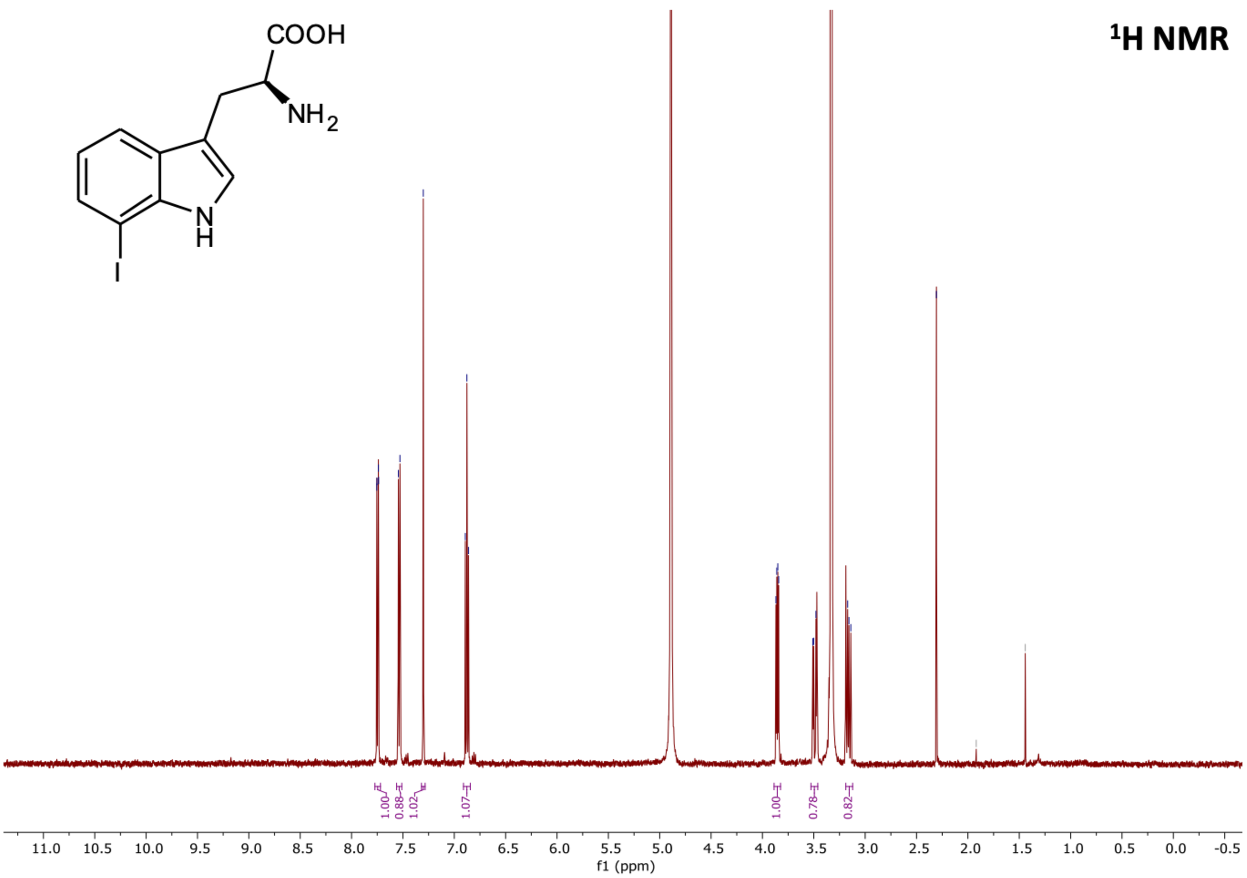

Supplementary Figure 52. ^{1}H NMR spectrum of (S)-2-amino-3-(7-iodo-1H-indol-3-yl)propanoic acid.

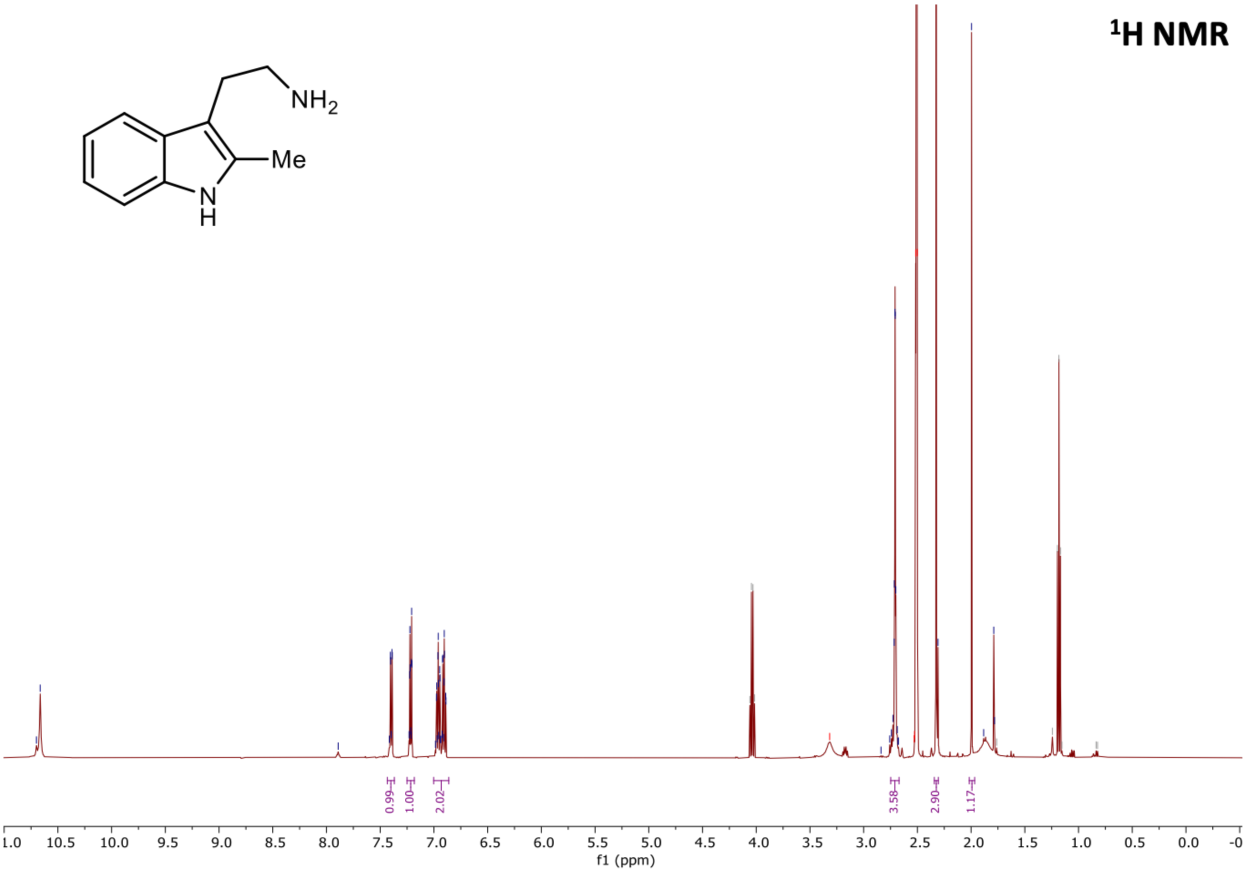

Supplementary Figure 53. ^{1}H NMR spectrum of 2-(2-methyl-1H-indol-3-yl)ethan-1-amine.

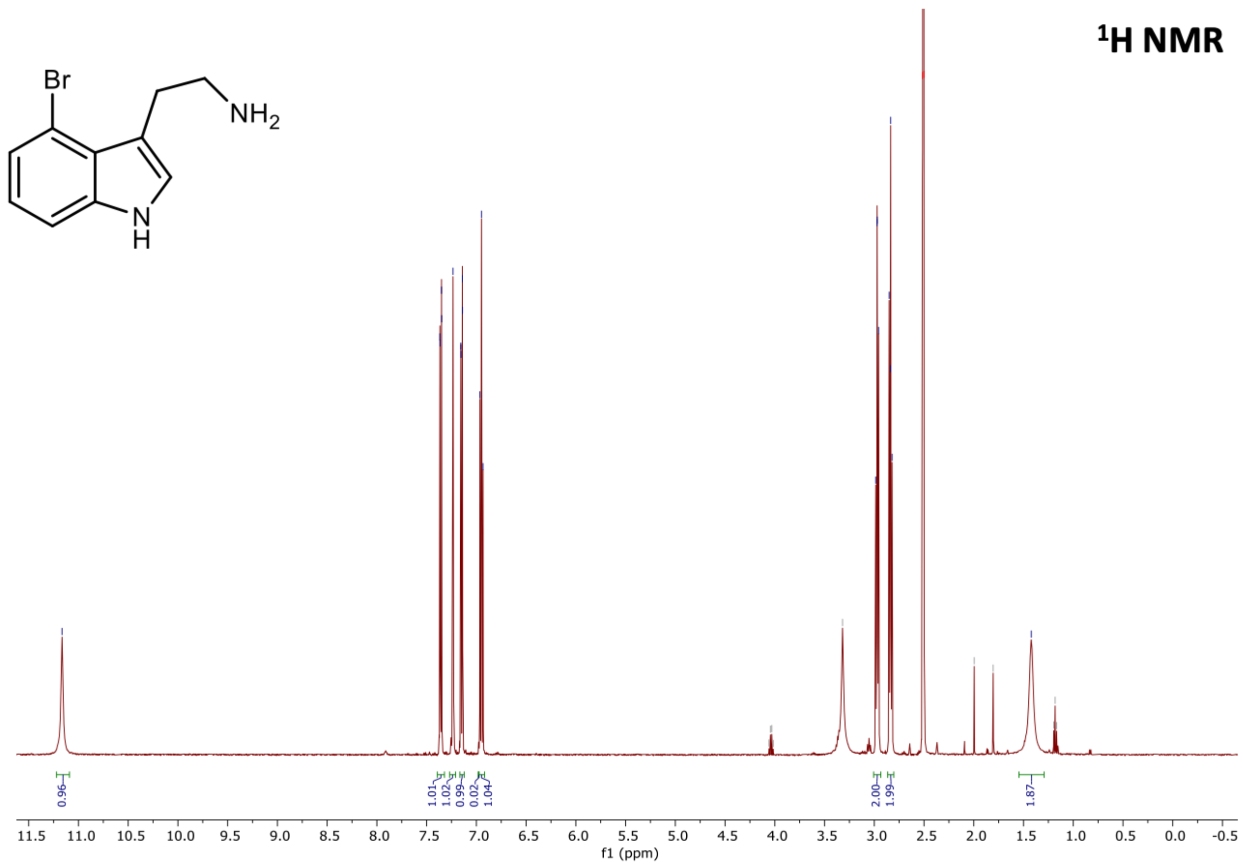

Supplementary Figure 54. ^{1}**H NMR spectrum of 2-(4-bromo-1H-indol-3-yl)ethanamine.**

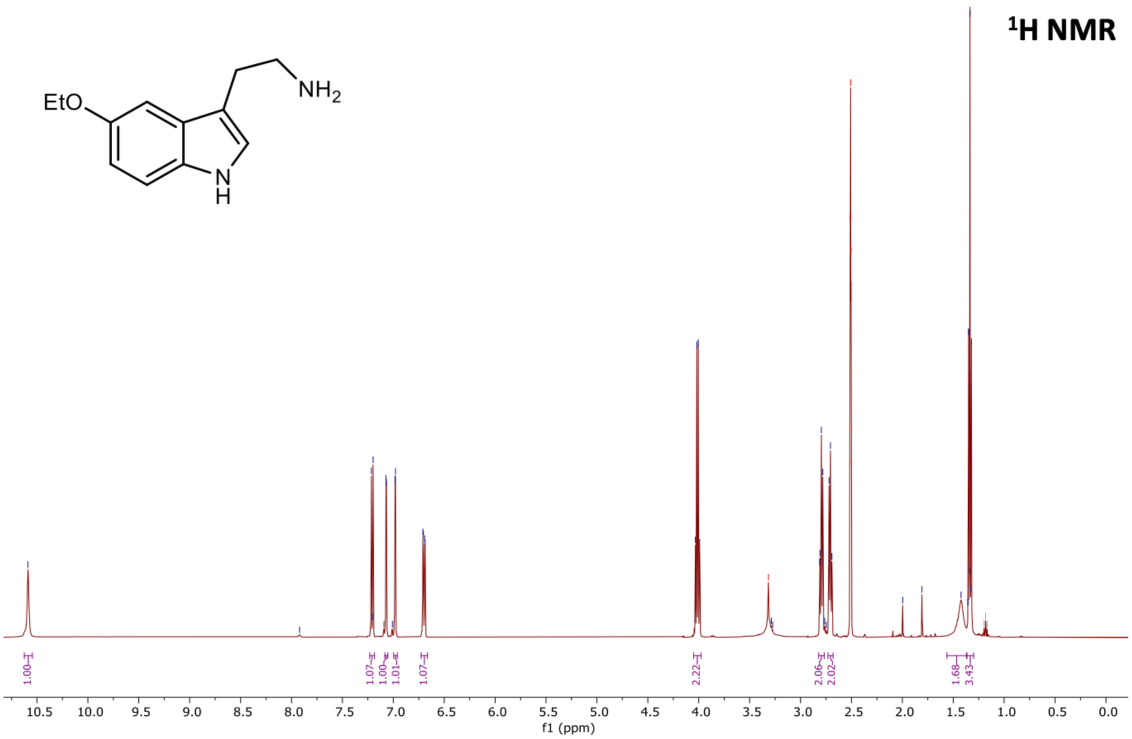

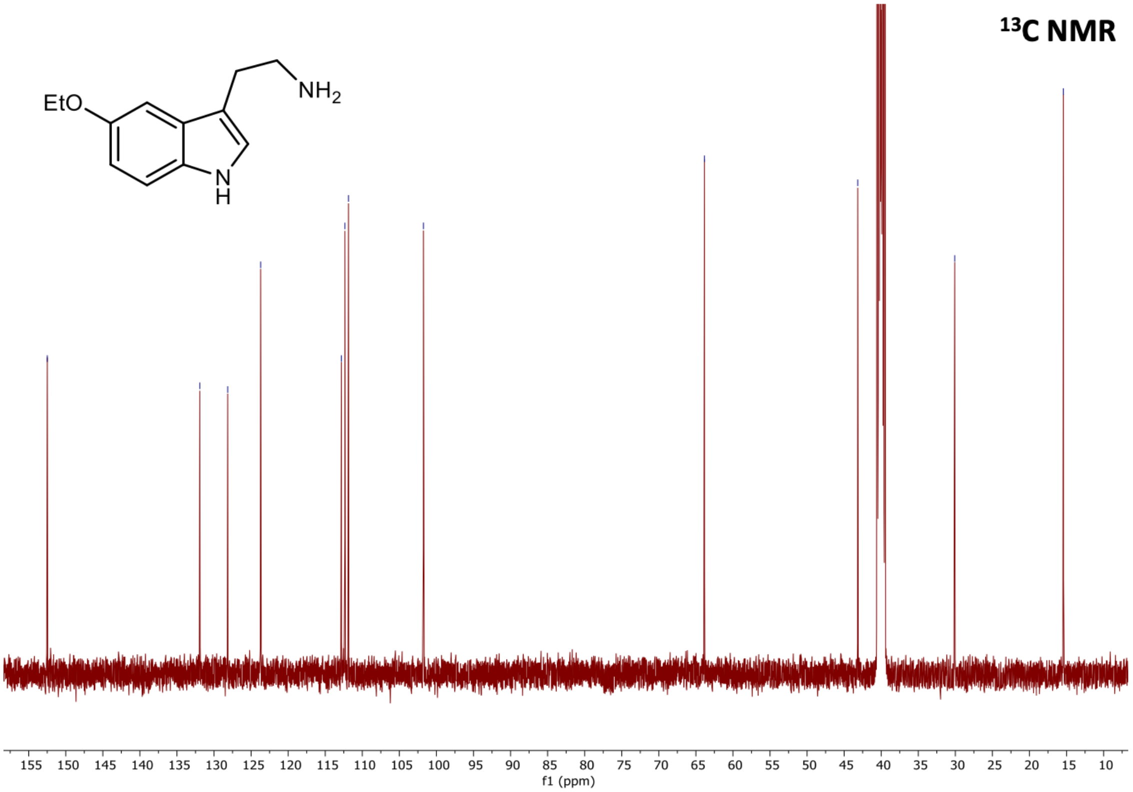

Supplementary Figure 55. ^{1}H and ^{13}C NMR spectra of 2-(5-ethoxy-1H-indol-3-yl)ethanamine.

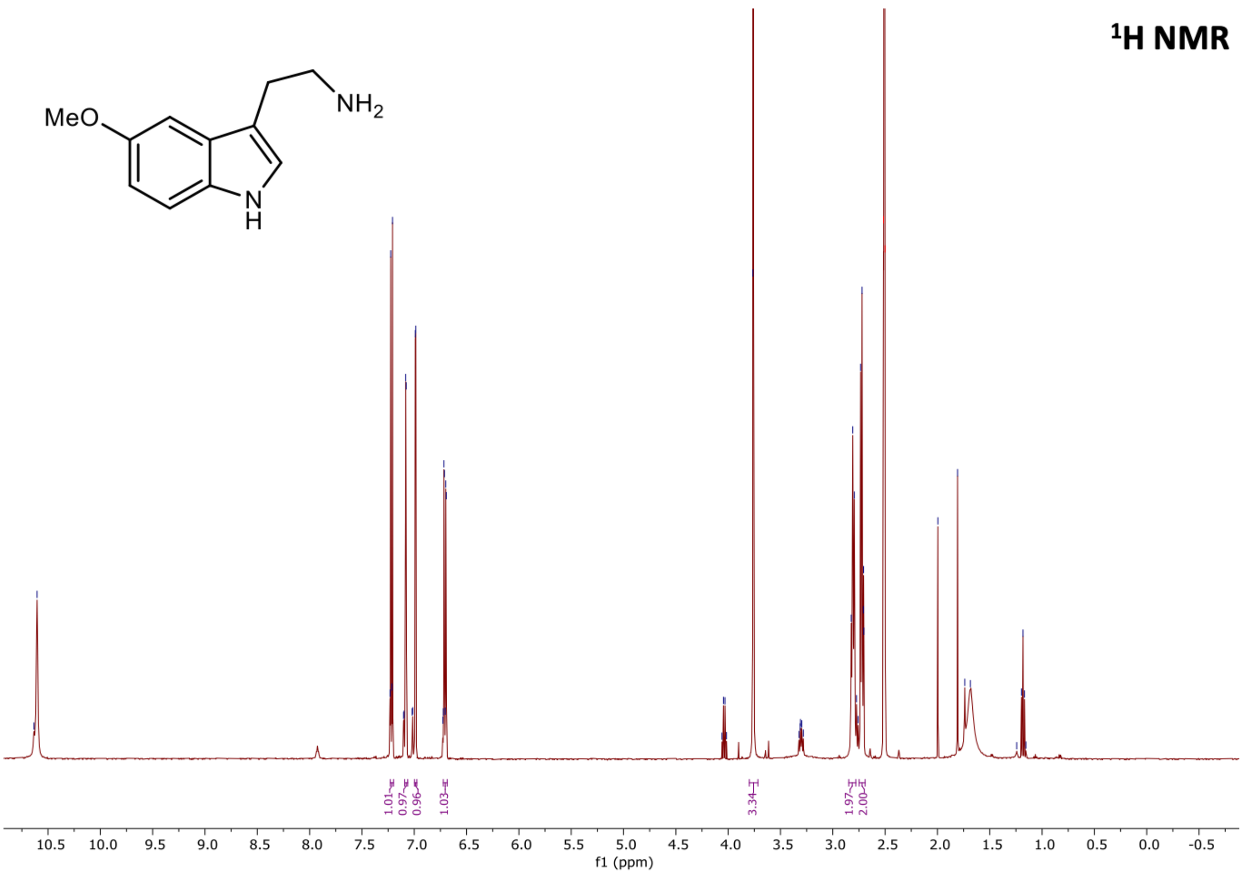

Supplementary Figure 56. ^{1}H NMR spectrum of 2-(5-methoxy-1H-indol-3-yl)ethanamine.

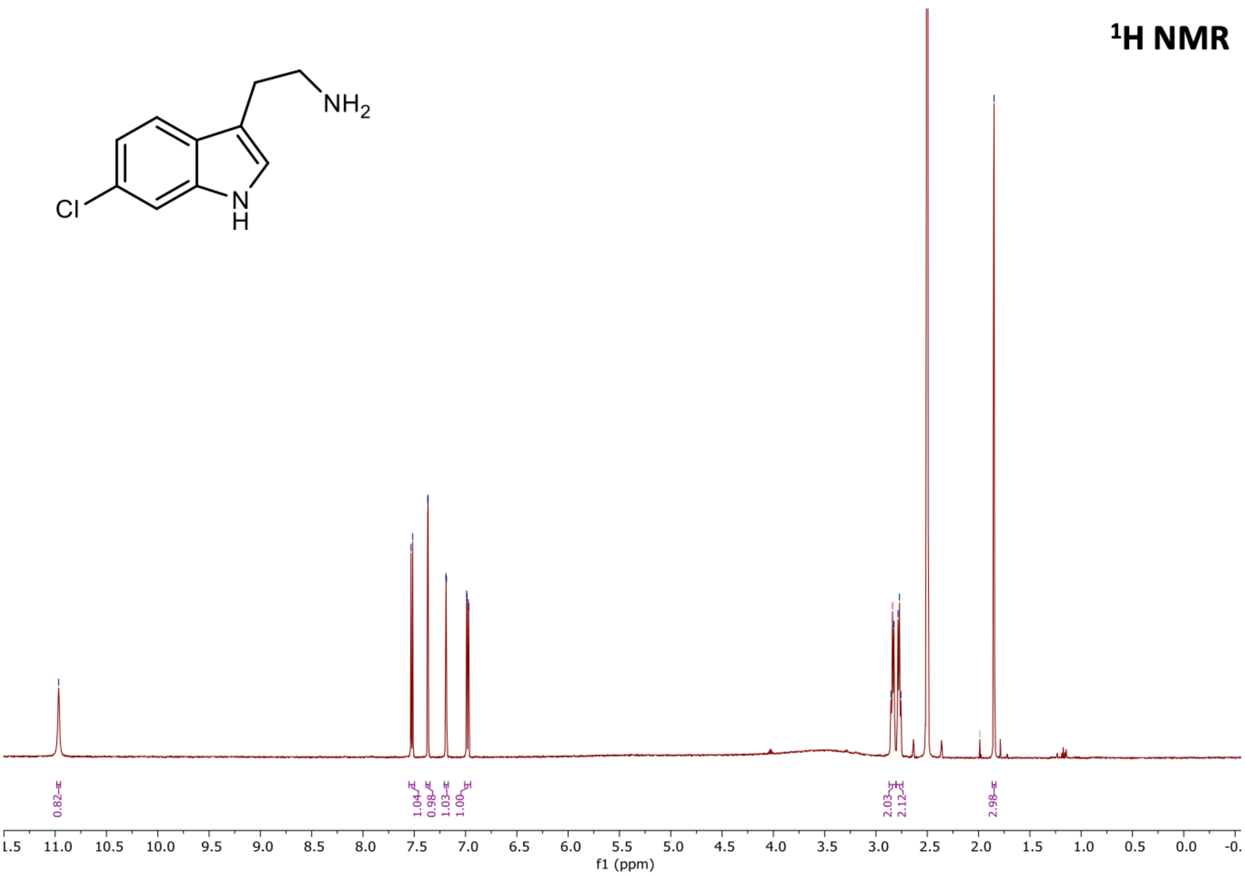

Supplementary Figure 57. ^{1}H NMR spectrum of 2-(6-chloro-1H-indol-3-yl)ethan-1-amine.

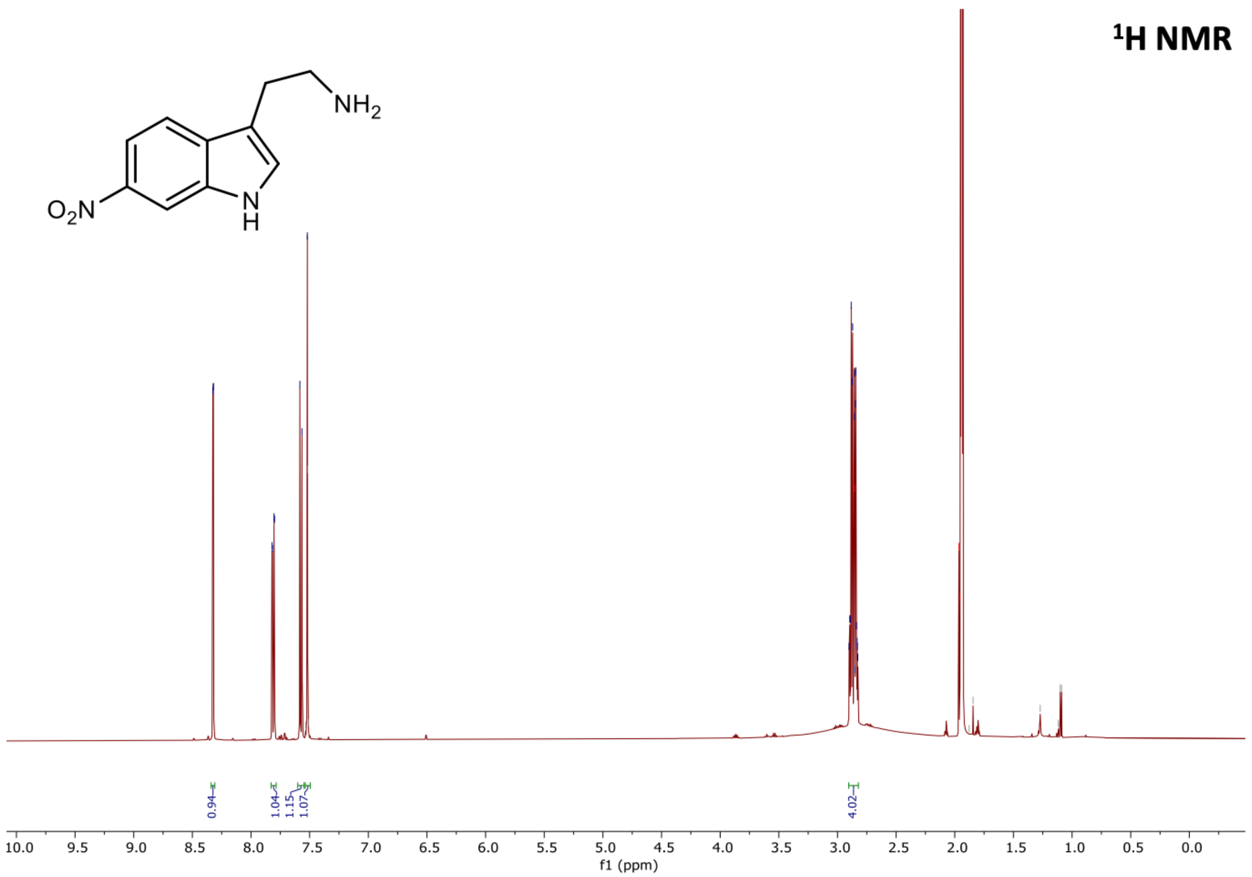

Supplementary Figure 58. **^{1}H NMR spectrum of 2-(6-nitro-1H-indol-3-yl)ethan-1-amine.**

Supplementary References

1. Williams, B. B. *et al.* Discovery and characterization of gut microbiota decarboxylases that can produce the neurotransmitter tryptamine. *Cell Host Microbe* **16**, 495–503 (2014).
2. McDonald, A. D., Perkins, L. J. & Buller, A. R. Facile in Vitro Biocatalytic Production of Diverse Tryptamines. *ChemBioChem* **20**, 1939–1944 (2019).
3. Buller, A. R. *et al.* Directed evolution mimics allosteric activation by stepwise tuning of the conformational ensemble. *J. Am. Chem. Soc.* **140**, 7256–7266 (2018).
4. Kille, S. *et al.* Reducing codon redundancy and screening effort of combinatorial protein libraries created by saturation mutagenesis. *ACS Synth. Biol.* **2**, 83–92 (2013).
5. Stanišić, A., Hüsken, A. & Kries, H. HAMA: A multiplexed LC-MS/MS assay for specificity profiling of adenylate-forming enzymes. *Chem. Sci.* **10**, 10395–10399 (2019).
6. Chou, T. C. & Talalay, P. A simple generalized equation for the analysis of multiple inhibitions of Michaelis-Menten kinetic systems. *J. Biol. Chem.* **252**, 6438–6442 (1977).
7. Cornish-Bowden, A. Enzyme Specificity : Its Meaning in the General Case. *J. theor. Biol.* **108**, 451–457 (1984).
8. Kuo, Y. M., Henry, R. A. & Andrews, A. J. Measuring specificity in multi-substrate/product systems as a tool to investigate selectivity in vivo. *Biochim. Biophys. Acta - Proteins Proteomics* **1864**, 70–76 (2016).
9. Knorrscheidt, A. *et al.* Simultaneous screening of multiple substrates with an unspecific peroxygenase enabled modified alkane and alkene oxyfunctionalisations. *Catal. Sci. Technol.* 6058–6064 (2021) doi:10.1039/d0cy02457k.
10. Gibson, D. G. *et al.* Enzymatic assembly of DNA molecules up to several hundred kilobases. *Nat. Methods* **6**, 343–345 (2009).
11. TerMaat, J. R., Pienaar, E., Whitney, S. E., Mamedov, T. G. & Subramanian, A. Gene synthesis by integrated polymerase chain assembly and PCR amplification using a high-speed thermocycler. *J. Microbiol. Methods* **79**, 295–300 (2009).
12. Herger, M. *et al.* Synthesis of β-Branched Tryptophan Analogues Using an Engineered Subunit of Tryptophan Synthase. *J. Am. Chem. Soc.* **138**, 8388–8391 (2016).
13. Johnson, K. A. New standards for collecting and fitting steady state kinetic data. *Beilstein J. Org. Chem.* **15**, 16–29 (2019).
14. Buller, A. R. *et al.* Directed evolution of the tryptophan synthase β-subunit for stand-alone function recapitulates allosteric activation. *Proc. Natl. Acad. Sci.* **112**, 14599–14604 (2015).
15. Kabsch, W. XDS. *Acta Crystallogr. Sect. D Biol. Crystallogr.* **66**, 125 (2010).
16. Evans, P. R. & Murshudov, G. N. How good are my data and what is the resolution? *urn:issn:0907-4449* **69**, 1204–1214 (2013).
17. McCoy, A. J. *et al.* Phaser crystallographic software. *urn:issn:0021-8898* **40**, 658–674 (2007).
18. Winn, M. D. *et al.* Overview of the CCP4 suite and current developments.

urn:issn:0907-4449 **67**, 235–242 (2011).
19. Winn, M. D., Isupov, M. N. & Murshudov, G. N. Use of TLS parameters to model anisotropic displacements in macromolecular refinement. *Acta Crystallogr. Sect. D Biol. Crystallogr.* **57**, 122–133 (2001).
20. Chen, V. B. *et al.* MolProbity: all-atom structure validation for macromolecular crystallography. *Acta Crystallogr. Sect. D Biol. Crystallogr.* **66**, 12 (2010).
21. Romney, D. K., Murciano-Calles, J., Wehrmüller, J. E. & Arnold, F. H. Unlocking Reactivity of TrpB: A General Biocatalytic Platform for Synthesis of Tryptophan Analogues. *J. Am. Chem. Soc.* **139**, 10769–10776 (2017).
22. Murciano-Calles, J., Romney, D. K., Brinkmann-Chen, S., Buller, A. R. & Arnold, F. H. A Panel of TrpB Biocatalysts Derived from Tryptophan Synthase through the Transfer of Mutations that Mimic Allosteric Activation. *Angew. Chemie - Int. Ed.* **55**, 11577–11581 (2016).
