# Supplementary material for: Substrate multiplexed protein engineering facilitates promiscuous biocatalytic synthesis
Source: Nat Commun. 2022 Sep 6;13:5242. doi: 10.1038/s41467-022-32789-w (PMC9448781; doi:10.1038/s41467-022-32789-w)
Supplement: Supplementary file 3 — Reporting Summary [file 41467_2022_32789_MOESM3_ESM.pdf]

nature portfolio

Corresponding author(s): Andrew R. Buller
Last updated by author(s): Aug 12, 2022

Reporting Summary

Nature Portfolio wishes to improve the reproducibility of the work that we publish. This form provides structure for consistency and transparency in reporting. For further information on Nature Portfolio policies, see our Editorial Policies and the Editorial Policy Checklist.

Statistics

For all statistical analyses, confirm that the following items are present in the figure legend, table legend, main text, or Methods section.

n/a	Confirmed	
☐	☒	The exact sample size (*n*) for each experimental group/condition, given as a discrete number and unit of measurement
☐	☒	A statement on whether measurements were taken from distinct samples or whether the same sample was measured repeatedly
☒	☐	The statistical test(s) used AND whether they are one- or two-sided<br>*Only common tests should be described solely by name; describe more complex techniques in the Methods section.*
☒	☐	A description of all covariates tested
☒	☐	A description of any assumptions or corrections, such as tests of normality and adjustment for multiple comparisons
☐	☒	A full description of the statistical parameters including central tendency (e.g. means) or other basic estimates (e.g. regression coefficient) AND variation (e.g. standard deviation) or associated estimates of uncertainty (e.g. confidence intervals)
☒	☐	For null hypothesis testing, the test statistic (e.g. *F*, *t*, *r*) with confidence intervals, effect sizes, degrees of freedom and *P* value noted<br>*Give P values as exact values whenever suitable.*
☒	☐	For Bayesian analysis, information on the choice of priors and Markov chain Monte Carlo settings
☒	☐	For hierarchical and complex designs, identification of the appropriate level for tests and full reporting of outcomes
☐	☒	Estimates of effect sizes (e.g. Cohen's *d*, Pearson's *r*), indicating how they were calculated

Our web collection on statistics for biologists contains articles on many of the points above.

Software and code

Policy information about availability of computer code

Data collection	Empower 3 Software (Waters) for analysis of LC-MS data.<br>X-ray crystallographic data was analyzed with the CCP4 software package.
Data analysis	Standard statistical averaging was performed in Excel from the Microsoft 365 MSO package; version 2207 Build 16.0.15427.20182.<br>Kinetic data were fit with PRISM, Graphpad software, version 8.4.0.<br>X-ray crystal structures were built in Coot, Marina Bay version 0.9.4.

For manuscripts utilizing custom algorithms or software that are central to the research but not yet described in published literature, software must be made available to editors and reviewers. We strongly encourage code deposition in a community repository (e.g. GitHub). See the Nature Portfolio guidelines for submitting code & software for further information.

Data

Policy information about availability of data

All manuscripts must include a data availability statement. This statement should provide the following information, where applicable:

- Accession codes, unique identifiers, or web links for publicly available datasets
- A description of any restrictions on data availability
- For clinical datasets or third party data, please ensure that the statement adheres to our policy

Structural data that support the findings of this study have been deposited in the Protein Data Bank with the PDB accession codes [7ROF, 7RNQ, 7RNP]. All other

data that support the findings of this study are available from the corresponding author upon request. DNA and primer sequence information is available in Supplementary Table 5. Supplementary data supporting the scientific claims, supplementary figures, as well as a supplemental discussion can be found in the Supplementary Information file.

Human research participants

Policy information about studies involving human research participants and Sex and Gender in Research.

Reporting on sex and gender	N/A
Population characteristics	N/A
Recruitment	N/A
Ethics oversight	N/A

Note that full information on the approval of the study protocol must also be provided in the manuscript.

Field-specific reporting

Please select the one below that is the best fit for your research. If you are not sure, read the appropriate sections before making your selection.

- [x] Life sciences
- [ ] Behavioural & social sciences
- [ ] Ecological, evolutionary & environmental sciences

For a reference copy of the document with all sections, see nature.com/documents/nr-reporting-summary-flat.pdf

Life sciences study design

All studies must disclose on these points even when the disclosure is negative.

Sample size	Sample size was determined by counting mutants from Sanger sequencing. Sample sizes are sufficiently large since most if not all possible mutations were observed.
Data exclusions	Data points from screened samples with no sequencing results were excluded from analysis, as we could not connect activity with sequence information.
Replication	Measurements of enzymatic activity were conducted in at least duplicate (for trends) or triplicate for statements about individual activity measurements. Activity measurements of samples from screening were often measured in biological replicate due to re-sampling of mutations. These replicate measurements are detailed in Supplementary Table 4.
Randomization	Sequence information was unknown at the time of screening for activity and was determined after screening was completed. Elsewhere, randomization was not applicable.
Blinding	Chemical safety requires the knowledge of chemical compounds used for these experiments.

Reporting for specific materials, systems and methods

We require information from authors about some types of materials, experimental systems and methods used in many studies. Here, indicate whether each material, system or method listed is relevant to your study. If you are not sure if a list item applies to your research, read the appropriate section before selecting a response.

Materials & experimental systems

n/a	Involved in the study	
x		Antibodies
x		Eukaryotic cell lines
x		Palaeontology and archaeology
x		Animals and other organisms
x		Clinical data
x		Dual use research of concern

Methods

n/a	Involved in the study	
x		ChIP-seq
x		Flow cytometry
x		MRI-based neuroimaging
